# A dual-action bacteriophage protein Ped1 degrades DNA and blocks replication in *Pseudomonas aeruginosa*

**DOI:** 10.1038/s44318-026-00924-8

**Published:** 2026-10-02

**Authors:** Tietao Wang, Wenbo Yan, Chenchen Wang, Longgang Sang, Yingxin Yang, Jingru Zhao, Chao Li, Lijun Liu, Yani Zhang, Shuai Le, Haihua Liang

**Affiliations:** 1 Key Laboratory of Resources Biology and Biotechnology in Western China, Ministry of Education, College of Life Sciences, Northwest University, Xi’an, China; 2 Department of Biochemistry, SUSTech Homeostatic Medicine Institute, School of Medicine, Southern University of Science and Technology, Shenzhen, China; 3 School of Basic Medical Science, Xi’an Medical University, Xi’an, China; 4 Department of Microbiology, Army Medical University, Chongqing, China

**Keywords:** DNA Replication, Recombination & Repair, Microbiology, Virology & Host Pathogen Interaction

## Abstract

To fully harness phage therapy against escalating antimicrobial resistance, it is imperative to elucidate the fundamental mechanisms by which viruses systematically dismantle host physiology. In this study, we identify Ped1, a protein from *Pseudomonas aeruginosa* phage PaoP5, which suppresses bacterial growth through two mechanisms: degradation of genomic DNA and direct inhibition of the replication machinery. Our 2.3-Å crystal structure and biochemical profiling reveal that Ped1 adopts a previously uncharacterized α/β-nuclease architecture, enabling the efficient cleavage of both double-stranded and single-stranded DNA. Furthermore, we demonstrate that Ped1 physically interacts with the host replicative helicase, DnaB, to suppress its activity during DNA replication. Reciprocally, DnaB binds Ped1 to neutralize its nuclease function, acting as an internal immunity partner. Evolutionary analysis indicates that Ped1 is a lineage-specific toxin strictly conserved among *Pseudomonas* phages. Collectively, this work identifies a novel, dual-function phage effector that coordinately disrupts genomic integrity and replication fidelity, thus advancing our understanding of the phage–host molecular arms race, and describing a promising scaffold for the development of next-generation antimicrobials against multidrug-resistant *P. aeruginosa*.

## Synopsis

Bacteriophage-encoded proteins that subvert host physiology provide insight into the molecular arms race between phages and their hosts. This study identifies Ped1, a protein from *Pseudomonas aeruginosa* phage PaoP5 that orchestrates host takeover via a two-pronged mechanism.

- Ped1 is a 74-amino-acid nuclease whose overexpression suppresses *P. aeruginosa* growth.
- The Ped1 crystal structure reveals a novel α/β-nuclease fold with a catalytic pocket that enables Mg²⁺-dependent cleavage of both dsDNA and ssDNA.
- Ped1 also directly binds and inhibits the host replicative helicase DnaB, with DnaB reciprocally antagonizing Ped1 nuclease activity.
- Deletion of *ped1* attenuates phage lytic activity and compromises its ability to serve as phage therapy against *P. aeruginosa* infection in vivo.

## Abstract (web summary)

An atypical phage nuclease inhibits the host replicative helicase and protects against *P. aeruginosa* infection in vivo.

## Graphical abstract

**Figure:**
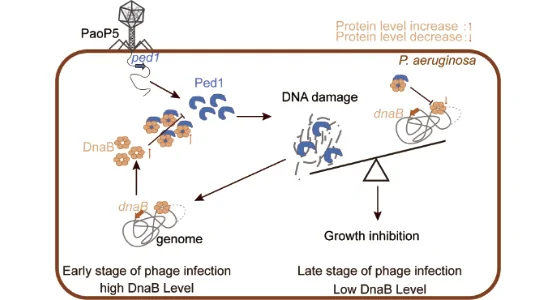

## Introduction

*Pseudomonas aeruginosa* is a leading opportunistic pathogen responsible for severe infections, particularly in immunocompromised individuals and patients with cystic fibrosis (CF), other forms of bronchiectasis, and severe chronic obstructive pulmonary disease (Rossi et al, 2021). Chronic infection and resulting pulmonary inflammation are primary contributors to mortality in patients with CF (Kohler et al, 2009; Rossi et al, 2021). The pathogen’s virulence is underpinned by a diverse arsenal of factors, including flagella, type IV pili, exopolysaccharide alginate, lipopolysaccharide, toxins, pyocyanin, proteases, and lipases (Balasubramanian et al, 2013). Compounding this intrinsic pathogenicity, *P. aeruginosa* exhibits formidable antibiotic resistance via mechanisms such as reduced membrane permeability, acquisition of antibiotic-modifying or inactivating enzymes, and biofilm formation (Qin et al, 2022). The rise of multidrug-resistant (MDR) strains, fueled by antibiotic overuse, underscores an urgent need for novel therapeutic strategies that circumvent traditional resistance pathways (Qin et al, 2022; Savoia, 2014). Bacteriophage therapy has emerged as a promising alternative against MDR pathogens. Its advantages include high specificity, low inherent immunogenicity, potent lytic activity, and a lack of cross-resistance with conventional antibiotics (Kortright et al, 2019; Roach et al, 2017). Phages exert their antibacterial effect by precisely targeting essential bacterial structures and processes, such as bacterial cell walls, energy metabolism, DNA, or other biomacromolecules (De Smet et al, 2021; Zhang et al, 2022).

The evolutionary arms race between bacteria and their viral predators has led to the characterization of a variety of phage-encoded proteins that subvert host physiology (Hampton et al, 2020). For example, the Sgl protein of ssRNA phage PP7 targets the lipid II export flippase MurJ, while the transmembrane YES complex [MraY, protein E, sensitivity to lysis D (SlyD)] of phage PhiX174 inhibits peptidoglycan biosynthesis of the cell wall; both actions result in phage-mediated cell lysis (Adler et al, 2023; Orta et al, 2023). In *P. aeruginosa*, the phage LUZ24 peptide Igy binds DNA gyrase GyrB to block replication, inhibiting bacterial growth (De Smet et al, 2021). Phage D3112 twitching inhibitory protein Tip inhibits bacterial type IV pili assembly/extension ATPase PilB, which results in decreased infection efficiency and reduced full virulence of *P. aeruginosa* (Chung et al, 2014). Similarly, DMS3 phage protein Aqs1 silences anti-phage defenses by interfering with both pilus assembly and quorum sensing (Shah et al, 2021). In response, bacteria have evolved sophisticated defense systems (Mayo-Munoz et al, 2022; Yirmiya et al, 2024). For example, the CRISPR system is widely known as a major anti-phage system, and its related regulatory mechanisms have been extensively studied (Arias et al, 2022; Hampton et al, 2020; Kim et al, 2022). The *P. aeruginosa* tail assembly blocker protein, Tab, interferes with the virion assembly pathway of invading phages (Patel et al, 2024). While in *E. coli*, the hybrid HigBAC-CmdTAC system offers a broad-spectrum anti-phage defense (Mets et al, 2024).

*Pseudomonas* phage PaoP5 is a virulent virus which was isolated from hospital sewage in Chongqing in 2016 by Shen et al (Shen et al, 2016). In a prior functional screen of PaoP5 early genes, we identified the hypothetical protein PaoP5_157 as a potent inhibitor of PAO1 growth (Le et al, 2024). Here, we characterize PaoP5_157, hereafter designated as Ped1 (phage-encoded DNase 1), as a novel dual-function phage effector. The 2.3-Å resolution crystal structure of Ped1 reveals a novel, unique α/β-nuclease fold and defines a catalytic pocket, which was validated through site-directed mutagenesis. Our results demonstrate that Ped1 expression triggers robust growth inhibition in *P. aeruginosa* by directly degrading host genomic DNA. Intriguingly, this nuclease activity is counteracted by the host replicative helicase, DnaB. However, this defense proves futile due to the dispensable role Ped1 plays during the phage infection; while DnaB neutralizes the DNase activity, Ped1 binding reciprocally impedes DnaB’s helicase function, thereby paralyzing the host replication machinery. Consistent with this dual-sabotage mechanism, global transcriptomic profiling reveals that Ped1 profoundly disrupts DNA metabolism and repair pathways, creating a transcriptional trap that hijacks host resources. Collectively, our work identifies Ped1 as a sophisticated, functionally redundant phage molecular weapon that coordinately undermines genomic integrity and replication, unveiling a new facet of the molecular arms race between bacteriophages and their bacterial hosts.

## Results

### Expression of bacteriophage *ped1* inhibits *P. aeruginosa* growth

**Figure 1:**
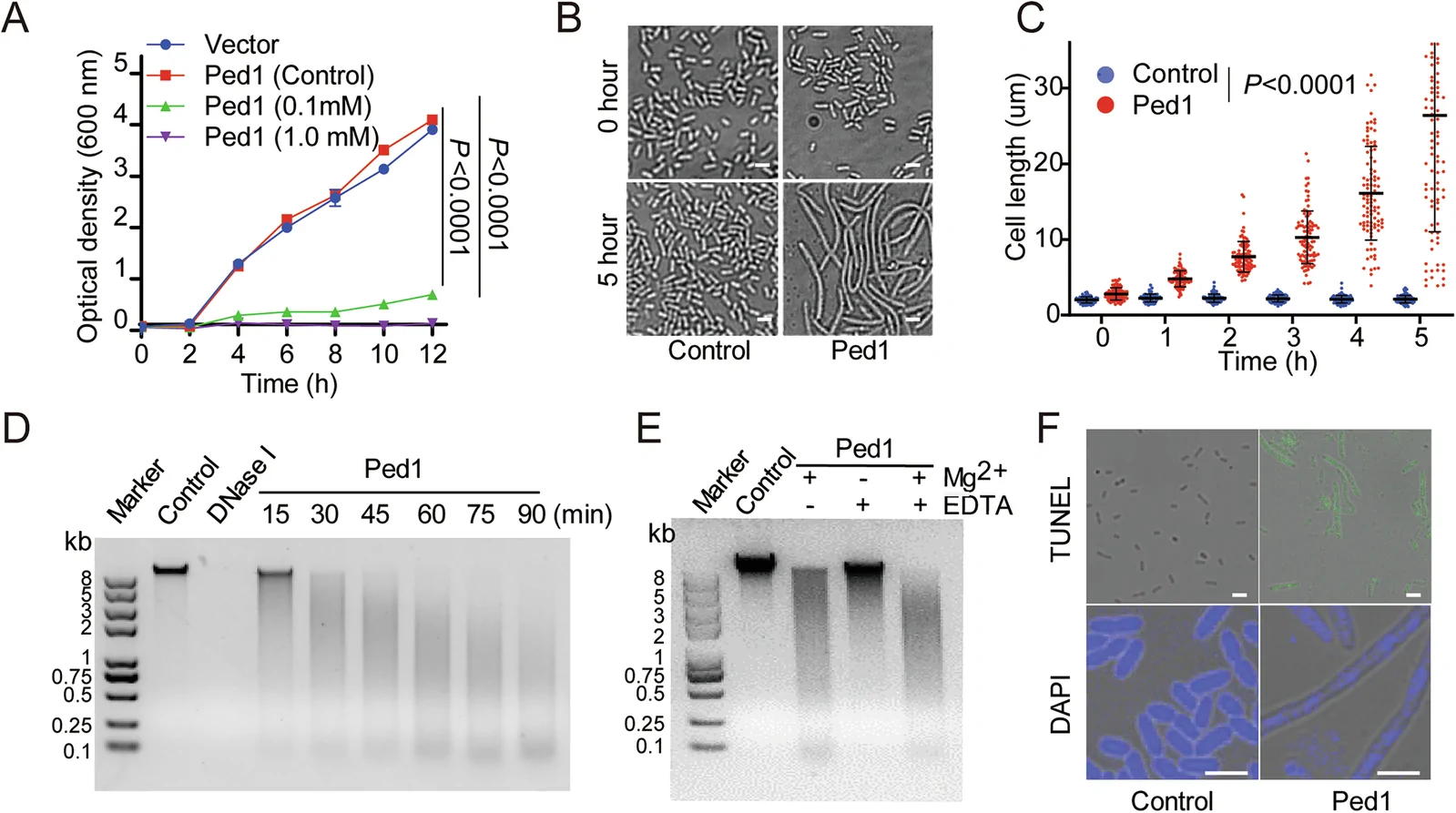
The phage effector Ped1 is a Mg^2+^-dependent DNase that inhibits the growth of *P. aeruginosa* PAO1. (**A**) Growth curve of PAO1 harboring an empty vector (Vector) or overexpressing Ped1 (Ped1) in LB liquid media supplemented with the indicated concentration of IPTG. (**B**, **C**) Ped1 induces filamentation of *P. aeruginosa* PAO1. (B) Representative micrographs of PAO1 cells expressing Ped1 at 0- and 5-h post-induction with IPTG. Scale bar, 2.5  μm. The complete time-course (0–5 h) is shown in Fig. EV1C. (**C**) Cell lengths of PAO1 producing Ped1 at the indicated time points in Fig. EV1C, *n* = 100. (**D**) In vitro DNase activity assay. *P. aeruginosa* genomic DNA was incubated with purified Ped1 in DNase I reaction buffer at 37 °C for the indicated time. Reaction products were resolved by agarose gel electrophoresis. DNase I served as a positive control. (**E**) Mg^2+^-dependence of Ped1 nuclease activity. *P. aeruginosa* genome DNA was co-incubated with purified Ped1 in DNase I reaction buffer with or without 1 mM EDTA or MgCl_2_ at 37 °C for 30 min. Products were analyzed by agarose gel electrophoresis. (**F**) In vivo detection of DNA degradation. PAO1 cells containing the indicated constructs were induced with IPTG for 4 h and subjected to DAPI staining (blue) and TUNEL assay (green; FITC-labeled). Representative merged fluorescence micrographs are shown; multichannel images are provided in Fig. EV3. Scale bar, 2.5 μm. Data are from three independent experiments (**A**–**F**). Error bars: mean ± SD. Statistical significance was determined by two-way ANOVA with Dunnett’s test (**A**, **C**). Source data are available online for this figure.

**Figure EV1:**
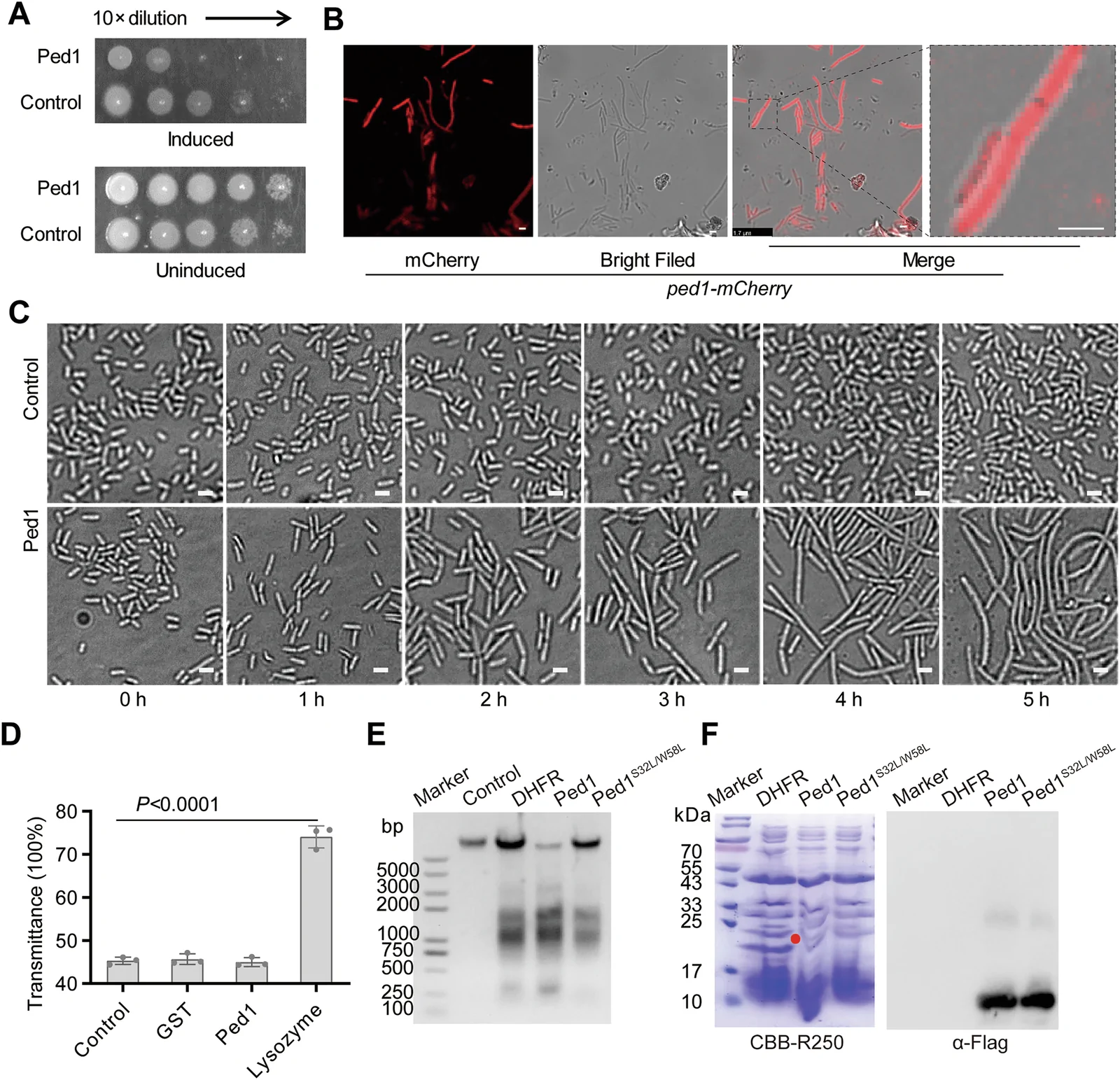
Localization and phenotypic characterization of Ped1-expressing cells. (**A**) Growth of *P. aeruginosa* PAO1 on LB agar plates. PAO1 cells harboring an empty vector (Control) or pME6032-ped1 (Ped1) were spotted as tenfold serial dilutions on plates with (Induced) or without (Uninduced) 1 mM IPTG. (**B**) Subcellular localization of Ped1-mCherry. Representative fluorescence, bright field, and merged micrographs of PAO1 cells expressing a Ped1-mCherry fusion protein. Ped1-mCherry is homogeneously distributed within the cytoplasm of filamentous cells. Scale bar, 2.5 μm. (**C**) Time-course microscopy of cell filamentation. Micrographs of PAO1 harboring an empty vector (Control) or expressing Ped1 taken at 1-h intervals (0–5 h) post-induction with IPTG. Scale bar, 2.5 μm. Images at 0 h and 5 h correspond to those presented in Fig. 1B. (**D**) Lysozyme activity assay. The transmittance (100%) of cell wall suspensions was measured after incubation with Control buffer, GST, purified Ped1, or Lysozyme (positive control). Ped1 exhibited no detectable lysozyme activity. (**E**) In vitro DNase activity assay. λ phage genomic DNA was incubated with the indicated in vitro synthesized Ped1 or Ped1^S32L/W58L^ in DNase I buffer containing 1 mM MgCl₂ at 37 °C for 60 min. DHFR served as a negative control for the DNase assay. (**F**) CBB-250 staining or western blot analysis of in vitro synthesized proteins. In all, 25-µl reactions containing 250 ng template DNA and 20 units RNase Inhibitor were incubated at 37 °C for 2 h. Two microliters of each reaction were analyzed by SDS–PAGE using a 12% Tris-glycine gel followed by CBB-250 staining or western blot. The red dot indicates the protein of interest. Data are representative of three independent experiments (**A**–**C**, **E**, **F**). Data are represented as mean ± SD from three independent experiments. Statistical significance was determined by two-way ANOVA (**D**). Source data are available online for this figure.

Heterologous expression of Ped1 (PaoP5_157) was previously shown to significantly inhibit the growth of *P. aeruginosa* PAO1 (Le et al, 2024). To further characterize the inhibition of Ped1, the cytoplasmic expression plasmid pME6032-*ped1* was transformed into wild-type *P. aeruginosa* PAO1 and induced with a gradient of IPTG concentrations in LB broth. Compared with the empty vector and non-induced control, induced Ped1 expression inhibits the PAO1 growth significantly (Figs. 1A and EV1A). BLAST and the conserved domain (CD) searches revealed that Ped1 lacks any detectable similarity to known functional proteins and contains no identifiable conserved domains. Beyond these initial observations, the molecular function of Ped1 remained unknown. To elucidate the molecular mechanism of Ped1, we first determined its subcellular localization by constructing a Ped1-mCherry fusion expression plasmid. Fluorescence microscopy image showed that Ped1-mCherry was homogeneously distributed within the cells and, notably, that cells expressing Ped1-mCherry became filamentous (Fig. EV1B). Next, we examined the cell morphology of Ped1-producing cells by microscopy and found that, unlike the empty vector control, PAO1 cells expressing Ped1 became filamentous (Fig. 1B). Quantification analysis revealed Ped1-expressing cells elongated to more than 15 times the length of vector control cells after 5 h of induction (Figs. 1C and EV1C). These results implied that Ped1 is a growth inhibitor that disturbs bacterial cell division in *P. aeruginosa*.

### Ped1 is a phage-encoded DNase

**Figure EV2:**
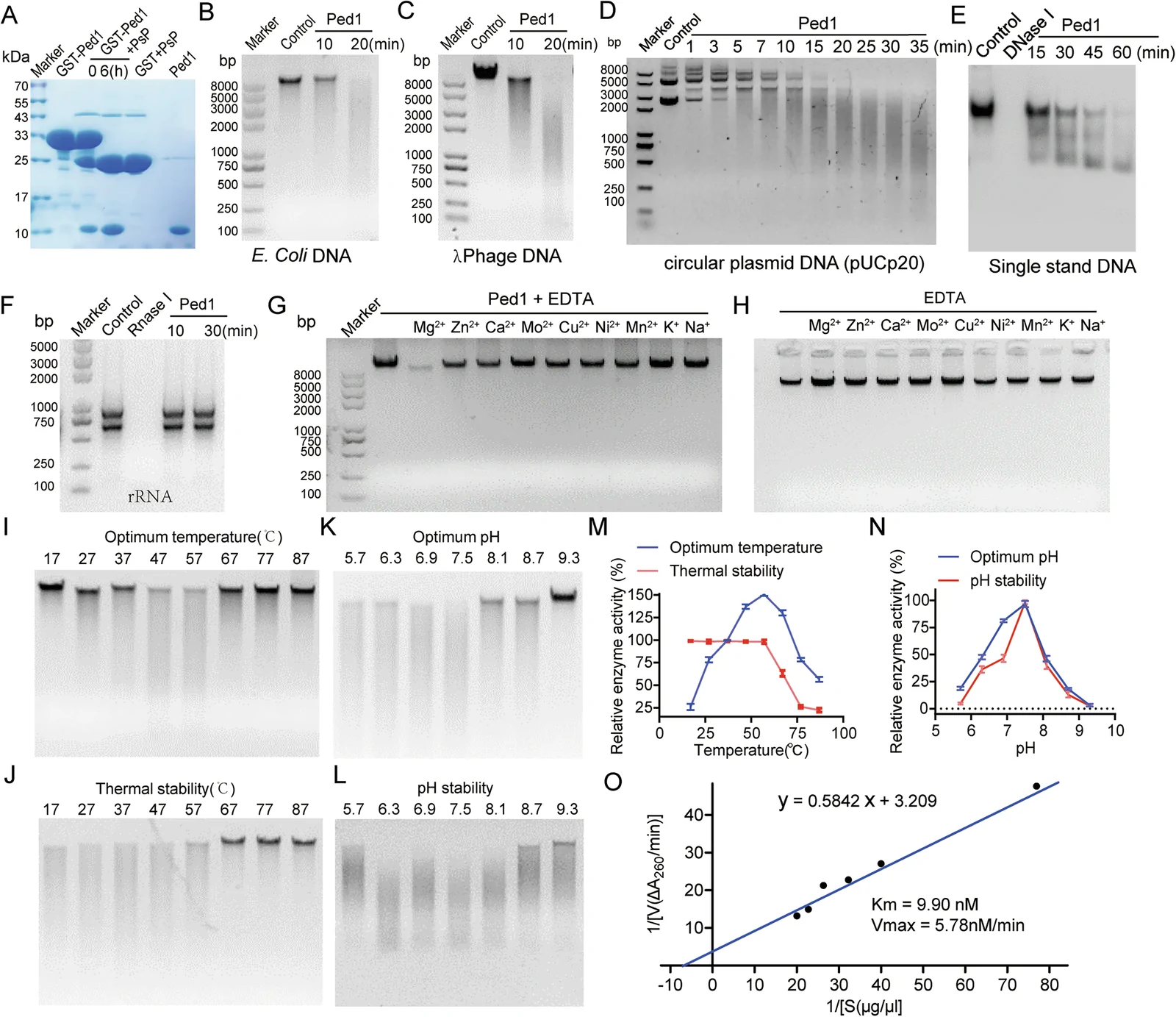
Biochemical characterization of Ped1 DNase activity. (**A**) Purification and GST-tag removal of GST-Ped1. (**B**, **C**) *E. coli* (**B**) or λ phage genomic DNA (**C**) was incubated with Ped1 in DNase I buffer containing 1 mM MgCl₂ at 37 °C for 20 min. Products were resolved by agarose gel electrophoresis. (**D**, **E**) Activity of Ped1 against circular plasmid DNA (pUCP20) (**D**) and single‑stranded DNA (**E**). DNase I served as a positive control. (**F**) RNA hydrolysis assay. Total RNA was incubated with Ped1 and analyzed by agarose gel electrophoresis. (**G**, **H**) Metal ion-dependency of Ped1 nuclease activity. Λ DNA was incubated in DNase I buffer containing a final concentration of 2 mM indicated metal ions for 10 min with (**G**) or without (**H**) Ped1. (**I**–**L**) Effects of temperature (**I**, **J**) and pH (**K**) on Ped1 nuclease activity against λDNA or its thermal stability. λDNA was incubated with Ped1 for 10 min at the indicated temperatures (**I**). Ped1 was pre‑incubated at the indicated temperatures for 10 min, then assayed for activity at 37 °C for 10 min (**K**). λDNA was incubated with Ped1 for 10 min at the indicated pH values (**K**). Products were analyzed using agarose gel electrophoresis. (**L**) Ped1 was pre‑incubated in buffers of the indicated pH for 10 min, then assayed at 37 °C for 10 min. (**M**) Optimal temperature and thermal stability of Ped1. (**N**) Optimal pH and pH stability of Ped1. (**O**) Lineweaver‑Burk plots for Ped1 activity with λDNA as substrate. Data are representative of three independent experiments (**A**–**I**). (**M**, **N**) Each point represents the mean ± SD of three independent experiments. Source data are available online for this figure.

Bacterial cell filamentation also indicated cell division inhibition, which can be caused by DNA damage, suppression of cell wall peptidoglycan synthesis, or interference with other associated systems (Chassaing and Cascales, 2018; Meir et al, 2023; Rothfield, 2003; Vedyaykin et al, 2019). First, the nucleic acids and peptidoglycan-degrading activity of the purified Ped1 were analyzed using *P. aeruginosa* genomic DNA as substrates and the lysozyme Assay Kit, respectively. Unexpectedly, Ped1 could degrade DNA, but did not exhibit lysozyme activity (Figs. 1D and Fig. EV1D). This result suggested that Ped1 has a potential nuclease activity. Ped1 nuclease activity also relies on the existence of Mg^2+^, as EDTA inhibited DNase activity and supplementation with excess Mg^2+^ could restore it (Fig. 1E). To eliminate the risk of nuclease contamination from bacterial hosts, we synthesized Ped1 using the PURExpress^®^ In Vitro Protein Synthesis system (NEB). The in vitro-synthesized protein exhibited DNase activity, confirming that the degradation activity is an intrinsic property of Ped1 (Fig. EV1E,F). Furthermore, the *E. coli* genomic DNA, λ phage DNA, circular plasmid pUCP20, and synthetic single-stranded DNA fragments were also degraded as substrates (Fig. EV2A–E). However, Ped1 did not exhibit detectable RNase activity in vitro (Fig. EV2F). These data demonstrate that Ped1 is a Mg^2+^-dependent endonuclease that cleaves DNA but not RNA. Furthermore, the enzyme properties of Ped1 were analyzed using λ phage DNA under different temperatures, pH levels, and metal ion conditions (Fig. EV2G–K). The results showed that the optimum temperature of Ped1 activity is 57 °C and that it is stable below 57 °C (Fig. EV2M). The optimum pH for both activity and stability was 7.5 (Fig. EV2N). Meanwhile, the *Km* for the purified Ped1 was estimated to be 9.90 nM with a *Vmax* of 5.78 nM/min (Fig. EV2O).

The in vivo DNase activity of Ped1 was further confirmed by terminal deoxynucleotidyl transferase dUTP nick-end labeling (TUNEL) and 4’,6-diamidino-2-phenylindole (DAPI) staining assay. The TUNEL-positive (FITC-labeled) cells were observed in PAO1 cells producing Ped1 (Fig. 1F; Appendix Fig. S1A,B). Furthermore, the DAPI staining showed that the DNA content in Ped1-producing cells was remarkably lower than that in vector control cells (Fig. 1F; Appendix Fig. S1C,D). Quantification showed that the DNA content in Ped1-expressing PAO1 cells was approximately 2-fold lower than that in vector control cells (Appendix Fig. S1D). These results clearly demonstrate that Ped1 is a bona fide DNase, suggesting that the abnormal cell growth and length were the possible result of DNA damage by Ped1 in vivo DNase activity.

### Crystal structure of Ped1 reveals a novel α/β-nuclease fold and defines its catalytic pocket

**Figure EV3:**
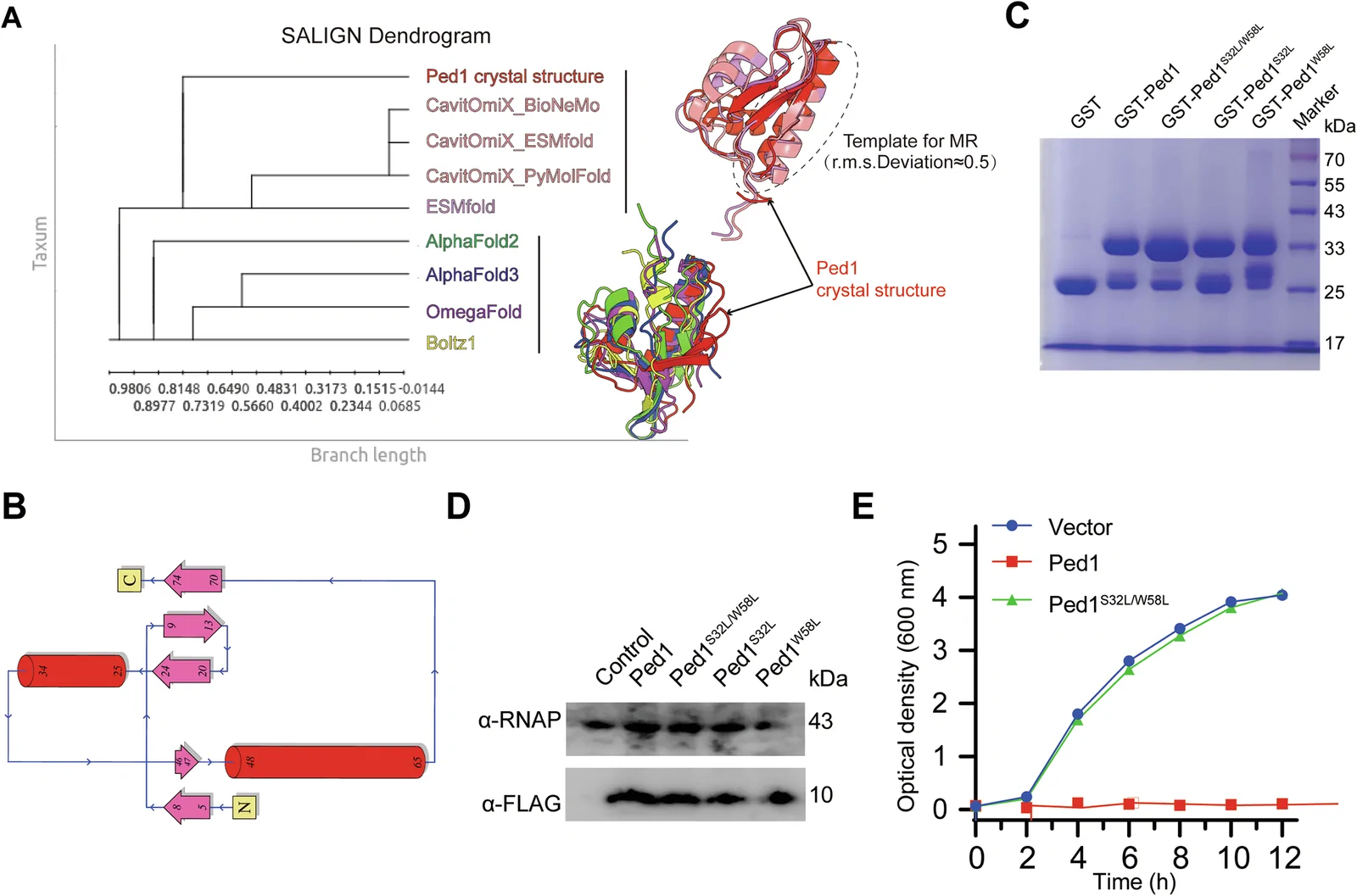
Structural comparison and mutational analysis of Ped1. (**A**) Comparison of experimentally determined and predicted Ped1 structures. A dendrogram (left) and superimposed structures (right) show the relationship between the experimental Ped1 crystal structure and various computational models. Despite structural predictions from tools like AlphaFold, critical backbone deviations necessitated the use of PyMol plugin CavitOmiX-generated fragments as search templates for molecular replacement. (**B**) Topological diagram of Ped1. This schematic illustrates the unique α/β-nuclease fold, characterized by a central three-stranded antiparallel β-sheet flanked by two α helices. (**C**) Purification of Ped1 variants. SDS–PAGE analysis confirms the successful purification of GST-tagged wild-type Ped1 and indicated variants (S32L/W58L, S32L, and W58L) used for downstream functional assays. (**D**) Western blot analysis of Ped1 expression. Immunoblotting with α-Flag confirms equivalent expression levels of wild-type Ped1 and its variants in PAO1 cells. α-RNAP was utilized as a loading control. (**E**) Growth dynamics of PAO1 expressing Ped1 variants. A growth curve monitored by OD_600_ demonstrates that while wild-type Ped1 causes severe growth inhibition, the catalytically inactive Ped1^S32L/W58L^ variant lacks physiological toxicity. Data representative of three independent experiments (**C**, **D**). Data are represented as mean ± SD from three independent experiments (**E**). Source data are available online for this figure.

To further verify whether the observed cell filamentation and physiological toxicity are directly triggered by nuclease activity, we first determined the high-resolution crystal structure of Ped1 for directed-mutation site design. Despite the advanced capabilities of modern protein structure prediction tools such as AlphaFold 2/3, the predicted models proved unsuitable as direct search templates for molecular replacement (MR) in solving the Ped1 structure. Attempts using seemingly conserved fragments extracted from AlphaFold predictions also failed, likely due to prominent and critical backbone deviations between the predicted and experimentally determined structures (Fig. EV3A). The initial phase problem was ultimately solved using partial fragments from the top-ranked model generated by the PyMOL plugin CavitOmiX.

**Figure 2:**
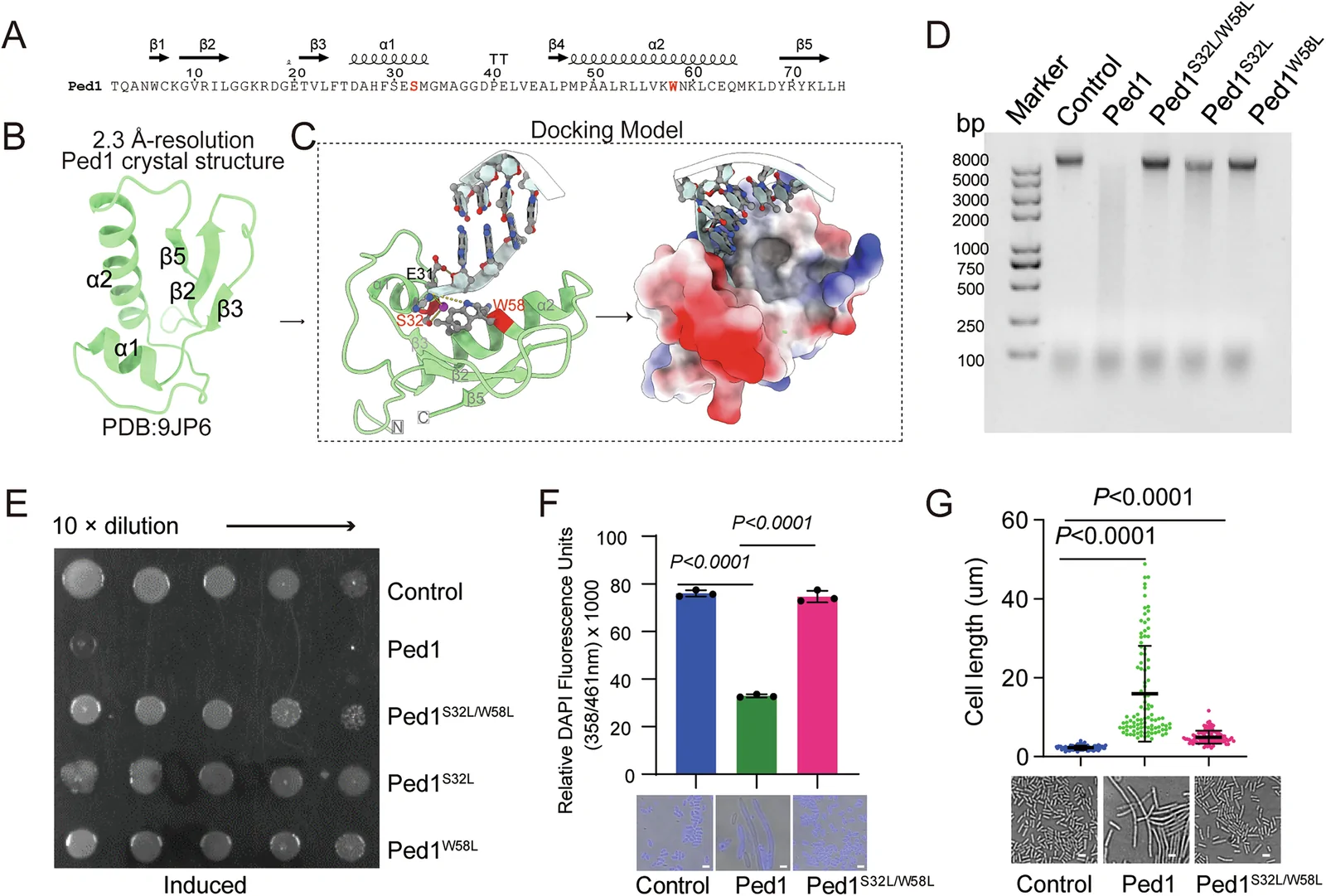
Crystal structure of Ped1 and identification of key residues for physiological toxicity. (**A**) Secondary structure of Ped1 defined by the DSSP algorithm (ENDscript server). Residues implicated in catalysis are highlighted in red. (**B**) Cartoon representation of the Ped1 crystal structure, with secondary structural elements labeled. (**C**) Ped1-dsDNA complex structure model (left) and its corresponding electrostatic surface potential (right). Interface-proximal residues are shown as ball-and-stick models, with key residues labeled in red. (**D**–**F**) S32 and W58 are essential for Ped1 function. (**D**) In vitro DNase activity assay. *P. aeruginosa* genomic DNA was incubated with purified wild-type Ped1 or the indicated mutants in DNase I reaction buffer at 37 °C for 30 min. Reaction products were analyzed by agarose gel electrophoresis. (**E**) Growth of *P. aeruginosa* PAO1 harboring empty vector (Control), wild-type Ped1, or the indicated variants on LB agar plates containing IPTG at 37 °C for 24 h. (**F**) Quantification of intracellular DNA content. Relative DAPI fluorescence units and corresponding 60x micrographs indicate that cells overexpressing the Ped1^S32L/W58L^ mutant maintain DNA levels comparable to cells in the control group, significantly higher than the depleted content in wild-type Ped1 cells (*P* < 0.0001). (**G**) The cell length quantification of PAO1 producing Ped1 or indicated variants at the indicated time points, *n* = 100 (upper). Representative micrographs acquired at 4 h post-induction (lower). Scale bar, 2.5 μm. Data shown are representative of three independent experiments (**D**–**G**). Error bars: mean ± SD. Statistical significance was determined by one-way ANOVA with Dunnett’s multiple (**F**, **G**). Source data are available online for this figure.

Structural similarity searches were carried out using Foldseek Search Server, with an E-value cutoff of 1e-3 and an RMSD (root mean square deviation) threshold of 5 Å. The result indicated that Ped1 has no experimentally determined structural homologs in current databases, thus classifying it as an orphan nuclease, which is consistent with DALI analysis (Dataset EV1). Its 2.3-Å resolution single crystal structure reveals a novel α/β fold (Figs. 2A,B and  EV3B). Structural analysis identified a prominent pocket, formed by a three-stranded antiparallel β-sheet (β2, β3, β5) flanked by α1 and α2 helices, as the putative active site (Fig. 2C). Molecular docking performed with the HADDOCK server positioned a catalytically essential Mg^2+^ ion within this pocket, which may be bound directly or indirectly by the side chains of S32 and W58.

To validate this prediction structural model, we purified Ped1 variants carrying single- or double-point mutations (Fig. EV3C). In vitro DNase activity assays demonstrated that the nuclease activities of all mutants (S32L, W58L, and S32L/W58L) were nearly abolished (Fig. 2D). Despite being expressed at levels comparable to the wild-type, these catalytically inactive variants lost the ability to trigger the bacterial toxicity observed upon Ped1 overexpression in *P. aeruginosa* PAO1 (Figs. 2E and  EV3D,E). Consistently, quantification of intracellular DNA showed that cells overexpressing the Ped1^S32L/W58L^ mutant retained DNA levels comparable to the control group, both of which were significantly higher than the depleted DNA content observed in cells overexpressing wild-type Ped1 (Fig. 2F). Furthermore, while wild-type Ped1 induced significant cell elongation, the Ped1 mutant proteins largely attenuated this phenotype (Fig. 2G). Taken together, our integrated structural and functional study of the orphan protein Ped1 reveals its novel α/β-nuclease fold, identifies the catalytic pocket and essential residues (S32 and W58), and establishes its DNase activity as one of the direct causes of physiological toxicity in *P. aeruginosa*.

### Ped1 interacts with the replicative DNA helicase DnaB to inhibit bacterial growth

**Figure 3:**
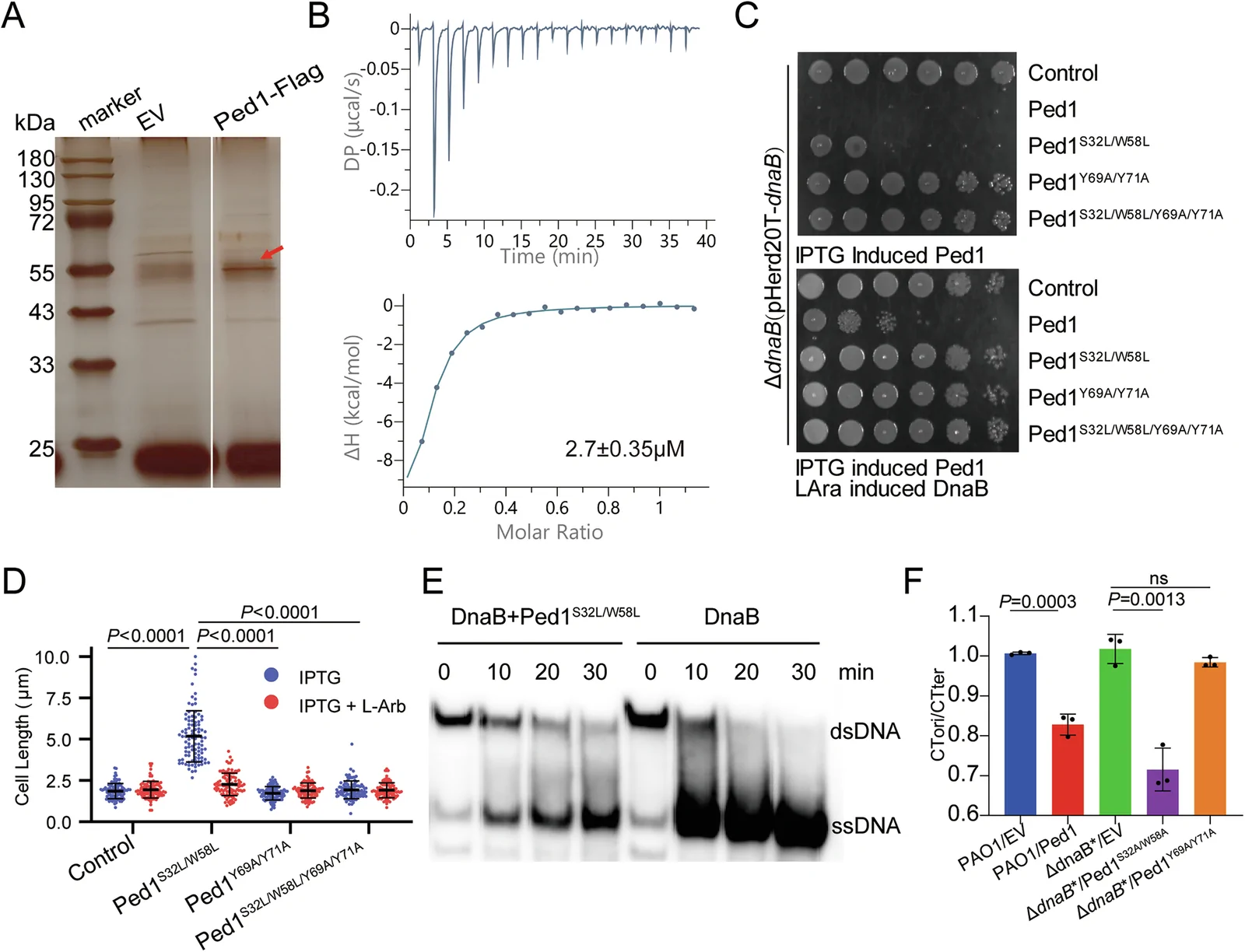
Ped1 directly interacts with the replicative helicase DnaB and inhibits its unwinding activity. (**A**) Identification of DnaB as a Ped1-binding protein. Silver-stained SDS–PAGE gel showing proteins co-purified with Ped1-Flag from PAO1 cell lysates. The specific band (red arrow) was identified by mass spectrometry as DnaB. (**B**) ITC analysis of the DnaB-Ped1 interaction. The thermogram shows raw heat signals (upper) and integrated binding isotherm (lower) from titrating Ped1 into DnaB. Data were fitted using a one-site binding model. (**C**) Growth of *P. aeruginosa* Δ*dnaB*(pHerd20T-*dnaB*) strains harboring empty vector (Control) or plasmids expressing the indicated Ped1 variants on LB agar with: IPTG (induce Ped1) or IPTG + arabinose (induce Ped1 and DnaB). Plates were incubated at 37 °C for 24 h. (**D**) Quantification of cell lengths from the strains overexpressing empty vector or indicated Ped1 mutants shown in Fig. EV4E, *n* = 100. The filamentation induced by wild-type Ped1 is abolished in inactive Ped1^Y69A/Y71A^ and Ped1^S32L/W58L/Y69A/Y71A^ mutants lacking DnaB-binding capacity, but not nuclease-inactive mutant Ped1^S32L/W58L^ with DnaB-binding capacity. (**E**) In vitro inhibition of DnaB helicase activity by Ped1. Helicase unwinding assays performed with purified DnaB in the presence or absence of the nuclease-inactive Ped1^S32L/W58L^ variant. Ped1 binding significantly inhibits the ability of DnaB to unwind double-stranded DNA (dsDNA) into single-stranded DNA (ssDNA) over time. (**F**) Replication activity assessed by *ter/ori* ratio. The CT(*ori/ter*) ratio was determined by qPCR in the indicated *P. aeruginosa* PAO1 or Δ*dnaB*(pHerd20T-*dnaB*) (ΔdnaB*) strains. Data shown are representative of three independent experiments (**B**, **C**, **E**, **F**). Error bars: mean ± SD. Statistical significance was determined by ordinary one-way ANOVA with Dunnett’s multiple comparisons test (**D**, **F**). Source data are available online for this figure.

Despite a marked decrease in cell length compared to cells overexpressing wild-type Ped1, cells overexpressing the catalytically inactive mutant (Ped1^S32L/W58L^) still exhibited a slightly elongated phenotype and the same DNA content level relative to the wild-type control, which implies the existence of an additional inhibition mechanism of Ped1 presumably by interacting with host protein, referring to replication or growth (Fig. 2D,F,G). To verify this hypothesis, the Ped1-targeted proteins were specifically enriched using co-immunoprecipitation. We constructed a C-terminal Flag-tagged Ped1 fusion protein expression plasmid pMMB67HE-*ped1-Flag*, which was transformed into *P. aeruginosa* PAO1. Cells were induced with a subinhibitory concentration of IPTG to express Ped1-Flag, and the cell lysates were purified with anti-Flag M2 magnetic beads. Ped1 retained on beads was detected by silver staining after sodium dodecyl sulfate-polyacrylamide gel electrophoresis (SDS–PAGE) (Fig. 3A). Mass spectrometry identified a specific ~52 kDa band as the replicative DNA helicase DnaB (PA4931), which was absent in the vector control (Appendix Fig. S2A). DnaB is a replicative DNA helicase that unwinds forked-duplex DNA substrate into single-strand DNA (Bailey et al, 2007; Kaplan and Steitz, 1999). We subsequently performed a series of complementary assays to confirm this interaction. In vitro, the direct binding between purified DnaB and GST-Ped1 was demonstrated by a clear peak shift in size-exclusion chromatography (SEC) and verified through GST pull-down assays (Appendix Fig. S2B,C). Furthermore, the interaction was recapitulated in a cellular context using a bacterial two-hybrid system (Appendix Fig. S2D). Quantitative analysis via isothermal titration calorimetry (ITC) using purified proteins revealed a Kd of 2.7 ± 0.35 µΜ, indicating that Ped1 binds DnaB with moderate affinity (Fig. 3B). To determine the stoichiometry of the DnaB-Ped1 complex in solution, we analyzed the purified complex by mass photometry (Appendix Fig. S2F). The mass distribution was dominated by a species of 329 kDa (70% of counts), in close agreement with the calculated mass of a DnaB hexamer bound to one Ped1 molecule (6×52.7 + 1×9.5 = 325.7 kDa), indicating that DnaB retains its hexameric assembly. The remaining events were distributed across additional minor peaks, reflecting a degree of compositional heterogeneity of the DnaB-Ped1 complex in solution (Appendix Fig. S2F).

**Figure EV4:**
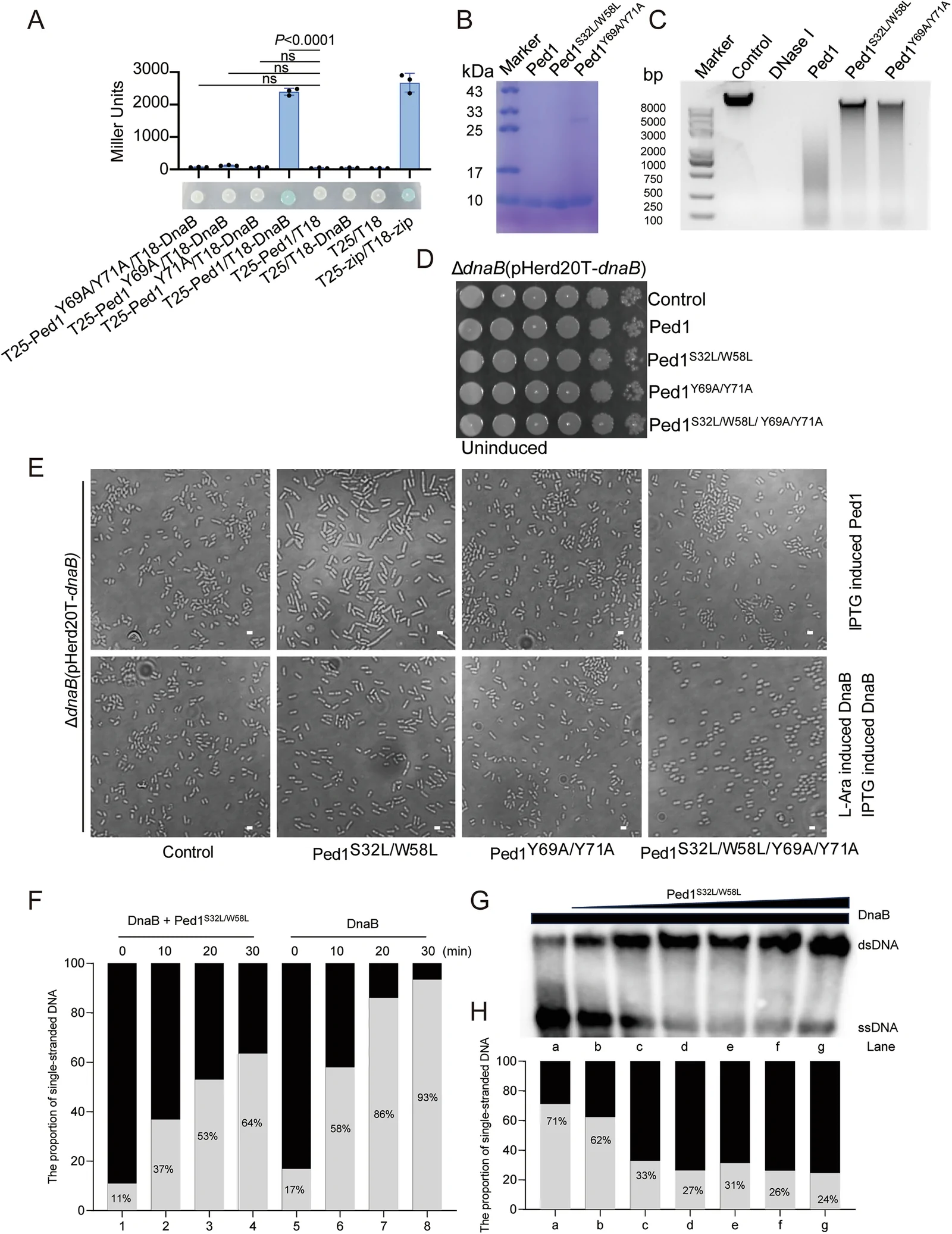
Mapping and functional characterization of the Ped1-DnaB binding interface. (**A**) Identification of residues critical for the Ped1-DnaB interaction. Bacterial two-hybrid (BACTH) analysis shows that the interaction with DnaB is abolished in Ped1 mutants harboring Y69A, Y71A, or Y69A/Y71A substitutions, as indicated by the lack of β-galactosidase activity (Miller Units) and white colony phenotypes on X-gal plates. (**B**) SDS–PAGE analysis of purified wild-type Ped1 and mutants (Ped1^S32L/W58L^ and Ped1^Y69A/Y71A^) used for biochemical assays. (**C**) Assessment of DNase activity in mutants. In vitro nuclease assays demonstrate that nuclease activity is abolished in the DNase-inactive mutant Ped1^S32L/W58L^ and the binding-deficient Ped1^Y69A/Y71A^. (**D**) Growth of *P. aeruginosa* PAO1*ΔdnaB*(pHerd20T-*dnaB*) strains harboring plasmids expressing the indicated Ped1 variants on LB agar without inducer added. (**E**) Representative phase-contrast micrographs of PAO1*ΔdnaB*(pHerd20T-*dnaB*) cells expressing the indicated Ped1 variants, which were acquired at 4 h post‑induction. Scale bar, 2.5 μm. (**F**) Quantification of the helicase activity in Fig. 3E. Band intensities of single-stranded and double-stranded DNA were quantified using ImageJ, and the percentage of unwound DNA (ssDNA/total DNA) was calculated for each lane. (**G**) In vitro inhibition of DnaB helicase activity by increasing concentrations of Ped1^S32L/W58L^. Helicase unwinding assays were performed with purified DnaB in the presence of increasing concentrations of the DNase-inactive Ped1 (Ped1^S32L/W58L^). (**H**) Quantification of the helicase assay in Fig. EV4F, performed as described in (**F**). Data are representative of three independent experiments (**B**–**E**, **G**). Data are represented as mean ± SD from three independent experiments. Statistical significance was determined by ordinary one-way ANOVA with Dunnett’s multiple comparisons test (**A**). Source data are available online for this figure.

The Ped1-DnaB interaction was further substantiated by directional mutational analysis; specifically, the interaction was completely abolished in the Ped1^Y69A^, Ped1^Y71A^, and Ped1^Y69A/Y71A^ variants (Fig. EV4A). Meanwhile, we also found that the DNase activity of the Ped1^Y69A/Y71A^ mutant was also abolished (Fig. EV4B,C). In contrast, the catalytically inactive Ped1^S32L/W58L^ variant retained their ability to bind DnaB, despite their nuclease activities being eliminated (Fig. EV4B,C; Appendix Fig. S2D). To further determine whether Ped1 binding to DnaB exerts a functional consequence on bacterial growth, we introduced the Ped1 variants in a conditional *dnaB* knockout strain. Under low DnaB expression (leaky expression), expressing the DnaB-binding-proficient but nuclease-dead mutant Ped1^S32L/W58L^ significantly inhibited bacterial growth, whereas the DnaB-binding-deficient mutants Ped1^Y69A/Y71A^ and Ped1^S32L/W58L/Y69A/Y71A^ failed to do so (Figs. 3C and  EV4D). Consistently, cell filamentation was observed in strains expressing Ped1^S32L/W58L^ but not in those expressing the binding-deficient mutants (Figs. 3D and  EV4E). Furthermore, in vitro helicase unwinding assays revealed that Ped1^S32L/W58L^ inhibited DnaB activity in a dose-dependent manner (Figs. 3E and  EV4F–H). To further investigate the DnaB-mediated DNA replication in vivo, we examined the chromosomal *ter*/*ori* ratio as a proxy of bacterial replication fork progression (Korem et al, 2015; Pang et al, 2017). As expected, expression of wild-type Ped1 significantly decreased the CT(*ori*/*ter*) ratio, indicating replication abnormalities, whereas the DnaB-binding-deficient mutant Ped1^Y69A/Y71A^ failed to decrease this ratio (Fig. 3F). Collectively, these findings demonstrate that Ped1 directly binds and inhibits DnaB helicase activity, thereby impairing host DNA replication independently of its DNase activity. However, the nuclease activity remains the dominant driver of the filamentation phenotype, as the DNase-deficient mutant caused only mild elongation compared to wild-type Ped1.

### The physiological toxicity of Ped1 is neutralized by DnaB

**Figure 4:**
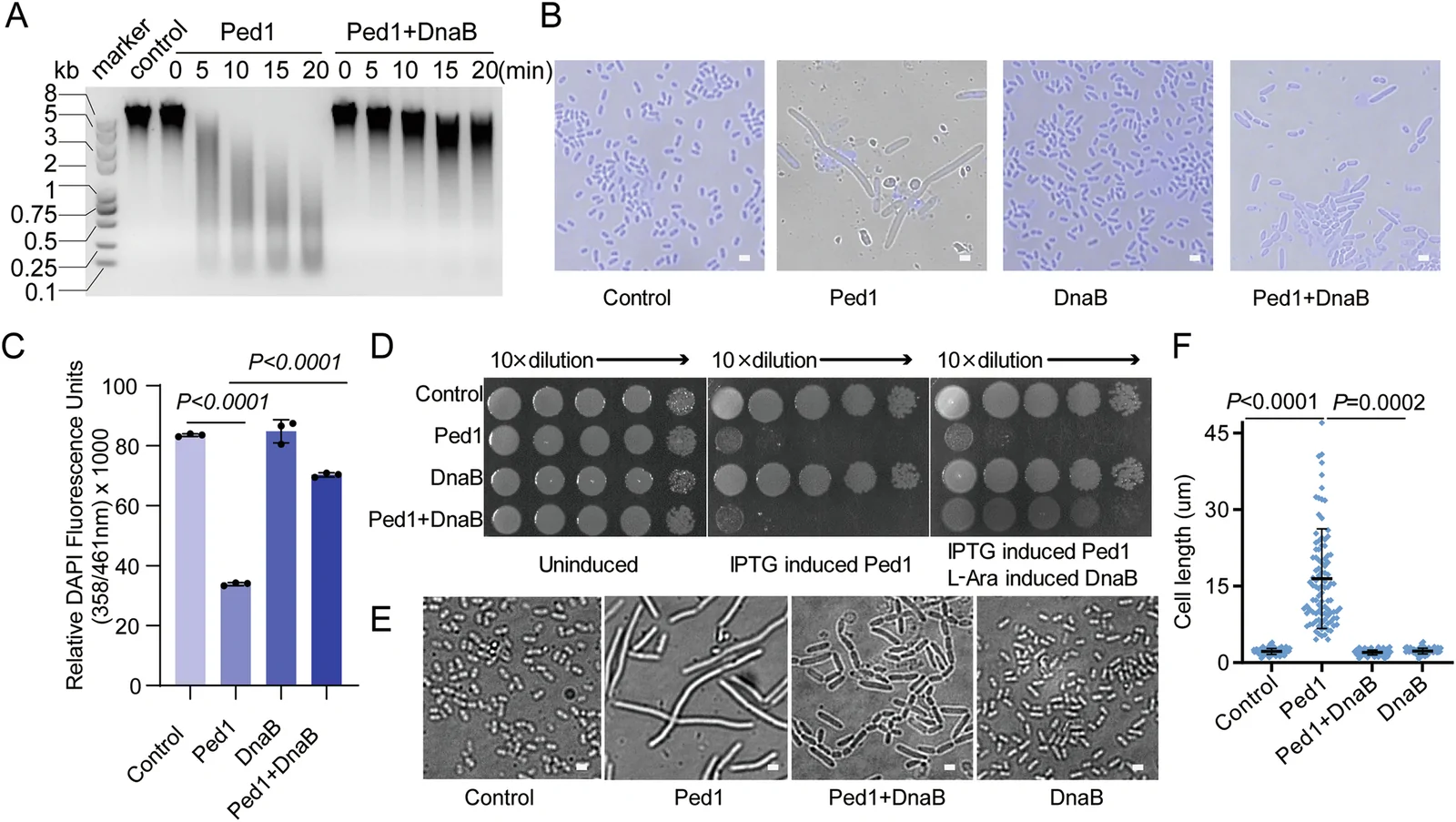
Host replicative helicase DnaB antagonizes Ped1 toxicity through direct inhibition of its nuclease activity. (**A**) Inhibition of Ped1 DNase activity by DnaB in vitro. *P. aeruginosa* genomic DNA was incubated with purified Ped1 in the presence or absence of purified DnaB. Agarose gel electrophoresis shows that while Ped1 alone completely digests the genomic DNA, the addition of DnaB protein effectively inhibits this degradation. (**B**, **C**) Quantitative analysis of intracellular DNA content via DAPI fluorescence and representative micrographs. Cells co-expressing Ped1 and DnaB maintain DNA levels comparable to control cells, significantly higher than the depleted DNA content observed in cells expressing Ped1 alone. Scale bar, 2.5 μm. (**D**) Neutralization of Ped1-induced growth inhibition by DnaB in vivo. Tenfold serial dilution assays on agar plates under various induction conditions. While Ped1 expression alone strongly suppresses bacterial growth, co-expression with DnaB restores growth to near wild-type levels. (**E**) Elimination of Ped1-induced cell filamentation. Micrographs captured 4 h post-induction show that the filamentation typically caused by Ped1 expression is abolished when DnaB is co-expressed, resulting in normal cell morphology. Scale bar, 2.5 μm. (**F**) Quantification of cell lengths from the strains shown in (**E**), *n* = 100. Data are from three independent experiments (**A**–**E**). Error bars: mean ± SD (**C**). Statistical significance was determined by ordinary one-way ANOVA with Dunnett’s test (**C**, **F**). Source data are available online for this figure.

Direct protein-protein interactions often lead to reciprocal functional modulation at the molecular level (Meyerkord and Fu, 2015; Roterman-Konieczna, 2013). As DnaB could directly interact with Ped1, we next examined whether DnaB overexpression could counteract Ped1-induced growth inhibition. To determine whether DnaB directly inhibits the DNase activity of Ped1, we performed an in vitro assay. *P. aeruginosa* genomic DNA was incubated with purified Ped1 in the presence or absence of purified DnaB protein at 37 °C for 20 min and analyzed by 1% agarose gel electrophoresis. Ped1 alone completely digested the genomic DNA, but this activity was inhibited by DnaB. The results demonstrate that DnaB directly inhibits the DNase activity of Ped1. In vitro DNA digestion assay shows nuclease activity of Ped1 could be inhibited by DnaB binding (Fig. 4A). Consistently, quantitative analysis of intracellular DNA content revealed that cells overexpressing the Ped1-DnaB complex maintained DNA levels comparable to those of the control group; both were significantly higher than the depleted DNA content observed in cells overexpressing wild-type Ped1 (Fig. 4B,C). As shown in Fig. 4D, the expression of Ped1 alone significantly inhibits PAO1 growth, whereas expression of DnaB alone or co-expression of Ped1 and DnaB in vivo did not inhibit the bacterial growth. However, it led to the filamentation elimination of PAO1 strains after 4 h of induction (Fig. 4E). Meanwhile, cell length was rescued by co-expressing DnaB with Ped1 (Fig. 4F). Taken together, these results establish the replicative helicase DnaB as a direct antagonist of the growth inhibitor Ped1 in *P. aeruginosa*.

### Ped1 acts as a functionally redundant decoy to modulate host *dnaB* expression and DNA metabolism

**Figure 5:**
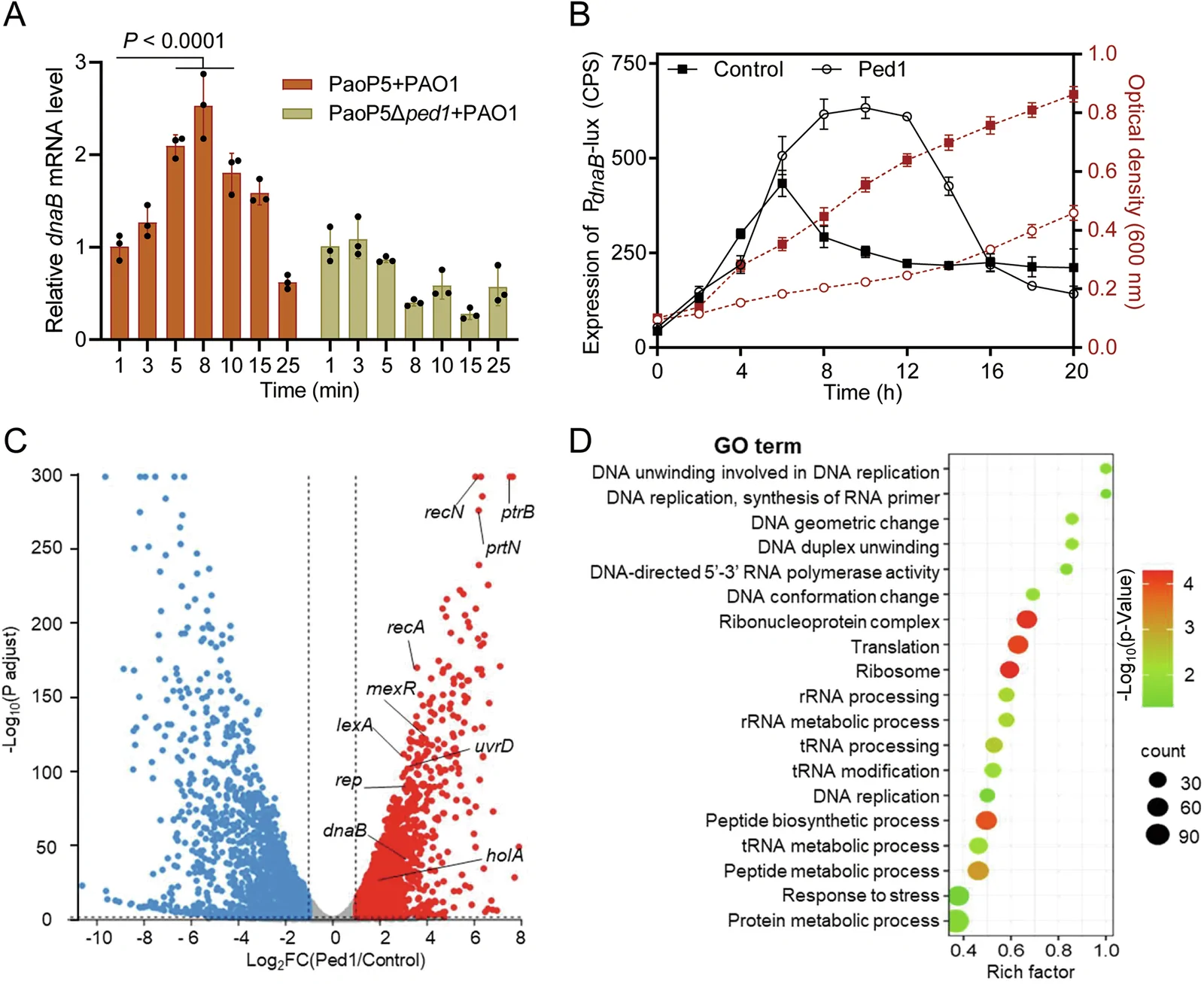
Ped1 triggers an SOS-like replication stress response and host transcription reprogramming during phage infection. (**A**) Temporal transcriptional dynamics of host *dnaB* during phage infection. Relative mRNA levels of *dnaB* were quantified by qRT-PCR in PAO1 cells infected with wild-type PaoP5 or PaoP5Δ*ped1* at the indicated time points post-infection. In wild-type infection, *dnaB* transcription surged slowly, peaking at 8 MPI, whereas this induction was significantly attenuated in the absence of ped1. (**B**) Validation of Ped1-induced *dnaB* expression via a luciferase reporter assay. The activity of the *dnaB* promoter (P*dnaB*-*lux*) was monitored over 20 h in cells expressing Ped1 compared to a vector control. (**C**) Global transcriptomic reprogramming induced by Ped1. A volcano plot displays differentially expressed genes (DEGs) between wild-type PAO1 and Ped1-expressing strains. DEGs were identified using DESeq2 with the Wald test. *P* values were adjusted by the Benjamini–Hochberg method. Blue and red dots indicate significantly up- and downregulated genes, respectively ( | log₂FC | > 1, adjusted *P* value < 0.05). Dashed lines indicate the thresholds for log₂FC = ± 1 and adjusted *P* value = 0.05. Key genes involved in DNA replication and repair (e.g., *dnaB*, *recA*, *lexA*, *uvrD*) are highlighted. (**D**) The gene ontology (GO) enrichment analysis of Ped1-induced DEGs. The bubble plot illustrates the top enriched biological processes. Notably, pathways associated with DNA replication, duplex unwinding, SOS response to stress, and protein metabolic processes were significantly overrepresented, reflecting the severe physiological disruption and the “transcriptional trap” created by Ped1 activity. The size and color of each dot represent the number of differentially expressed genes and the −Log_10_(*P* value) ranges, respectively. Statistical significance was determined using the Wald test with Benjamini–Hochberg correction for multiple testing (**C**). Data are representative of three independent experiments, error bars represent mean ± SD (**A**, **B**). Statistical significance was determined by ordinary one‑way ANOVA with Dunnett’s multiple comparisons test (**A**). Source data are available online for this figure.

Because Ped1 and DnaB physically interact and function as mutual antagonists, we reasoned that their relative expression during phage infection would be critical to understanding their interplay. To this end, we monitored the transcriptional dynamics of *ped1* and *dnaB* throughout the PaoP5 infection cycle by RT-qPCR. Following infection with wild-type PaoP5, the transcript levels of *dnaB* peaked at 8 min post-infection (MPI), representing a potent DNA replication stress response as the host attempted to replenish its replication machinery to neutralize Ped1-mediated damage (Fig. 5A). However, this induction was markedly attenuated in the absence of *ped1* (Fig. 5A). Furthermore, we observed the transcript level of *ped1* approximately 2-fold higher than that of *dnaB* throughout most of the infection cycle (Appendix Fig. S3). This Ped1-dependent modulation of *dnaB* expression, likely a consequence of the host-mediated stress response triggered by DNA damage, was further recapitulated through heterologous expression at subinhibitory levels (Fig. 5B).

To comprehensively profile the global host response to Ped1, we performed RNA-seq analysis comparing wild-type *P. aeruginosa* PAO1 to a Ped1-expressing strain. The robustness of these transcriptomic findings was independently validated by quantitative RT-PCR (qRT-PCR) of 11 representative genes, which exhibited strong concordance with the RNA-seq results (Appendix Fig. S4A). Ped1 expression induced massive transcriptional reprogramming, involving the significant upregulation of 1856 genes and the down-regulation of 1927 genes (Fig. 5C; Dataset EV2). Notably, 249 genes critical for DNA replication and repair, *recA*, *lexA*, and *uvrD* included, showed significant differential expression, a result highly consistent with the identified in vivo DNase activity of Ped1 (Fig. 5C; Datasets EV3 and 4). Gene Ontology (GO) enrichment analysis demonstrated that these differentially expressed genes span multiple essential functional categories, including nucleic acid metabolism, genomic replication, and translation (Fig. 5D). Beyond DNA metabolism, the global impact of Ped1 is further evidenced by the significant suppression of host metabolic categories, such as amino acid catabolism and biosynthetic processes (Appendix Fig. S4B,C). Collectively, these results demonstrate that while Ped1 is functionally redundant for phage viability, it serves as a sophisticated molecular weapon that sabotages genomic integrity and systematically hijacks host resources through the modulation of its binding partner, DnaB, and the broader metabolic landscape.

### Deletion of *ped1* significantly compromises phage infectivity

**Figure EV5:**
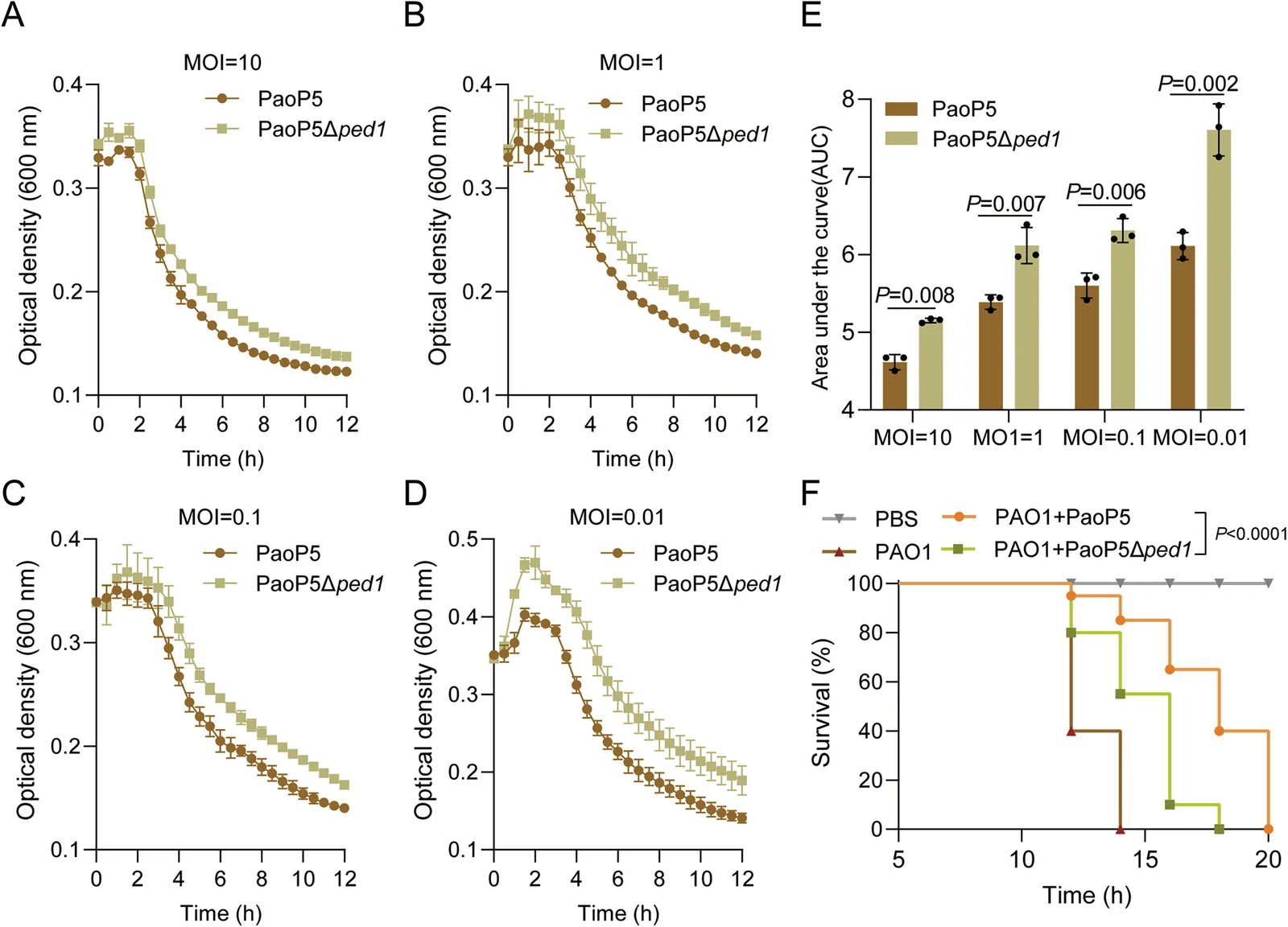
Deletion of *ped1* significantly compromises phage infectivity. (**A**–**D**) Bacterial lysis kinetics of *P. aeruginosa* PAO1 infected with PaoP5 or PaoP5Δ*ped1* at MOIs of 10, 1, 0.1, and 0.01. Bacterial density (OD_600_) was monitored every 30 min for 12 h post-infection. (**E**) Quantification of the area under the curve (AUC) for (**A**–**D**). (**F**) Survival curves of *Galleria mellonella* larvae infected with PAO1 and treated with PBS, wild-type PaoP5, or PaoP5Δ*ped1*. *n* = 20. Error bars: mean ± SD (**A**–**E**). Statistical significance was determined by unpaired *t* test (**E**). Statistical significance was calculated using the log-rank test (**F**). Source data are available online for this figure.

To investigate the physiological role of Ped1 in the phage PaoP5 life cycle, we examined the plaque morphology, phage particle morphology, and one‑step growth curve of PaoP5Δ*ped1*. The results showed that, compared with wild‑type PaoP5, PaoP5Δ*ped1* exhibited no significant differences in plaque size, morphology, or intracellular proliferation kinetics within 120 min post‑infection (Appendix Fig. S5A,B). We next evaluated the lytic activity of PaoP5Δ*ped1* against PAO1 by infecting at MOIs of 10, 1, 0.1, or 0.01 and monitoring bacterial growth in real‑time via optical density measurements (Fig. EV5A–D). The results demonstrated that PaoP5Δ*ped1* exhibited attenuated lytic activity compared to the wild‑type phage at all tested MOIs (Fig. EV5A–E), indicating that Ped1 is critical for efficient phage‑mediated host lysis. In parallel, we assessed the in vivo impact of PaoP5Δ*ped1* on PAO1 pathogenicity using a *Galleria mellonella* larva infection model. The results showed that PaoP5Δ*ped1* was also markedly less effective in attenuating PAO1 pathogenicity (Fig. EV5F). Collectively, these findings indicate that although Ped1 is dispensable for the phage basic infection cycle, it significantly enhances the overall lytic activity of phage PaoP5, thereby improving phage–host‑killing capacity and fitness.

### Ped1-like DNase is strictly distributed in *Pseudomonas* phages

**Figure 6:**
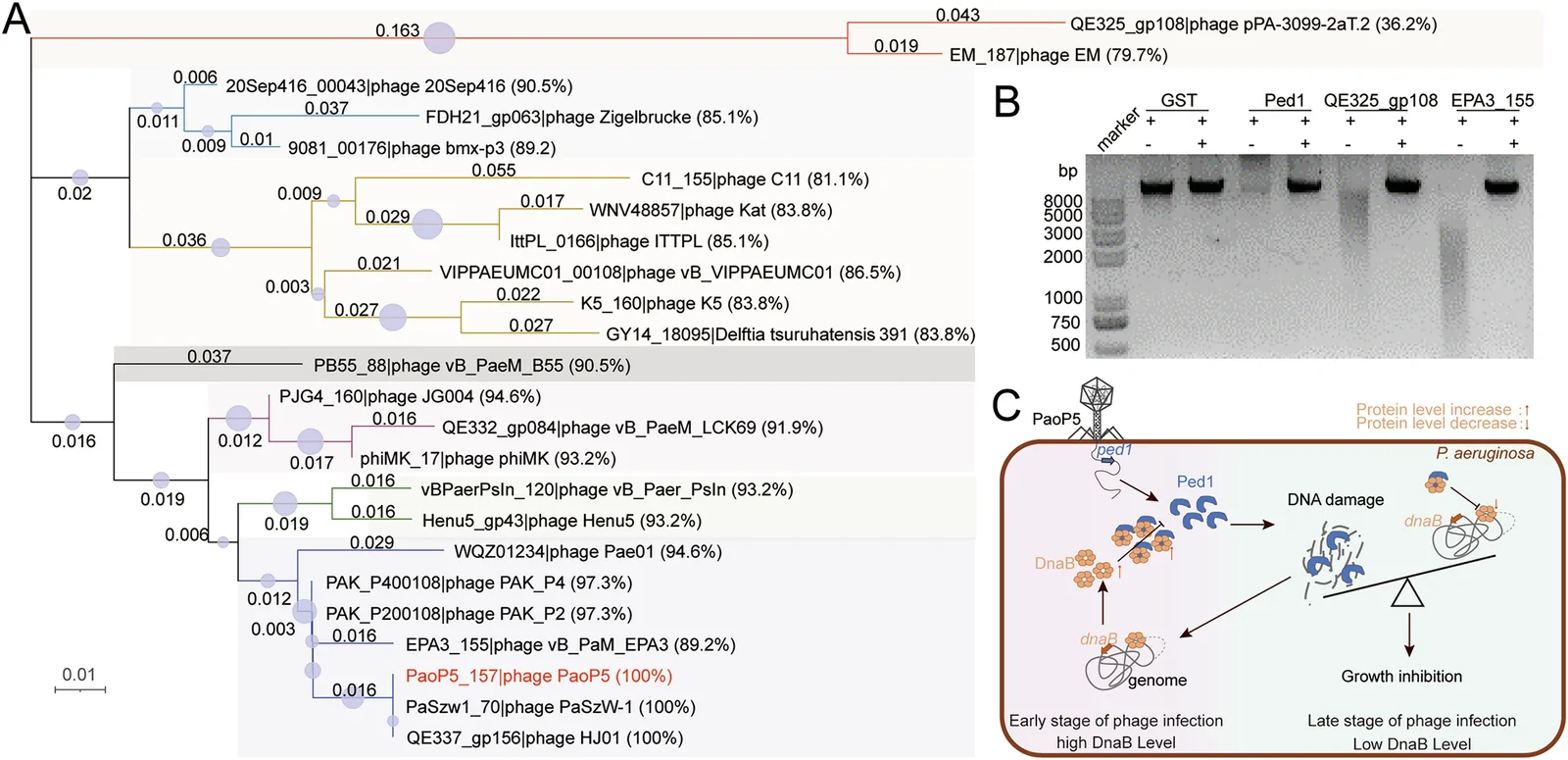
Phylogenetic analysis of Ped1 homologs and a proposal model of the Ped1-DnaB molecular arms race. (**A**) Evolutionary conservation and phylogenetic distribution of Ped1-like proteins. Ped1 homologs are exclusively restricted to phages infecting *Pseudomonas* species, suggesting a lineage-specific evolutionary adaptation within this genus. (**B**) Functional validation of representative Ped1 homologs. Purified proteins of two representative homologs, QE325_gp108 and EPA3_155, were incubated with *P. aeruginosa* genomic DNA. Both homologs exhibited potent DNase activity comparable to that of PaoP5 Ped1, confirming the conservation of nuclease function across this distinct phage protein lineage. (**C**) Conceptual model of the “Transcriptional Quicksand” trap and host subversion. Schematic illustrating the dynamic interplay between Ped1 and DnaB during the phage infection cycle. (i) Early Stage of Infection: Ped1 expression triggers genomic DNA damage and directly binds DnaB. In response to this replication stress, the host sharpens its defense by upregulating *dnaB* transcription to replenish the functional helicase pool. (ii) Late Stage of Infection: This defensive surge inadvertently creates a “transcriptional trap” where newly synthesized DnaB is immediately sequestered and neutralized by accumulating Ped1. Ped1 acts as a functional mimic, hijacking host resources and effectively steering host physiology toward deterministic lysis through its dual-hit attack on DNA integrity and replication machinery. Source data are available online for this figure.

To examine the evolutionary conservation of Ped1, we conducted a phylogenetic analysis based on its identified homologs. A BLASTp search retrieved 24 Ped1-like protein sequences (Fig. 6A), which were aligned and used to construct a neighbor-joining tree in MEGA, and a sequence alignment was also carried out (Appendix Fig. S6A). The resulting phylogeny showed that Ped1 homologs are exclusively distributed among phages infecting *Pseudomonas* species (Fig. 6A). Notably, two representative homologs, QE325_gp108 and EPA3_155, were confirmed to possess DNase activity comparable to that of PaoP5 Ped1 (Fig. 6B; Appendix Fig. S6B). These findings indicate that Ped1 represents a distinct lineage of phage-encoded nucleases that are strictly associated with *Pseudomonas* phages, likely playing a conserved role in phage–host interactions within this bacterial genus.

## Discussion

The evolutionary arms race between bacteria and phages has driven the diversification of viral effectors that act as precision weapons against host physiology. In this study, we identified and characterized Ped1, a dual-function effector from phage PaoP5 that orchestrates a two-hit strike by simultaneously degrading genomic DNA and hijacking the replication machinery. Crucially, we found that the interaction between Ped1 and the host replicative helicase DnaB is not a simple unidirectional suppression but a dynamic reciprocal antagonism. DnaB acts as an “internal immunity partner” by occupying key residues on Ped1 (e.g., Y69 and Y71), thereby neutralizing its nuclease activity and mitigating genomic damage (Fig. EV4A–C). This molecular tit-for-tat likely suggests that the infection outcome may initially hinge on the intracellular stoichiometric ratio of Ped1 to DnaB (Fig. 6C). By simultaneously targeting DNA integrity and the replication motor, Ped1 ensures a catastrophic collapse of host metabolism that cannot be fully reversed by the host’s compensatory upregulation of DnaB. Therefore, the existence of Ped1 effectively narrows the host’s survival window, steering the molecular conflict toward a deterministic lysis outcome, regardless of the host’s transient stress responses.

Structural homology searches using the Dali server yielded no genuine homologs (Z-score <4.0, excluding the self-match 9jp6-A), with top hits sharing less than 15% sequence identity (Dataset EV1). This confirms that Ped1 defines an unusual, structurally distinct class of phage-encoded endonucleases with an α/β-nuclease fold (Fig. 2A–C), which is distinct from the ββα-metal fold of the HNH superfamily and the αβββαβ fold of the PD-(D/E)XK superfamily (Govindaraju et al, 2016; Wu et al, 2020). The nuclease activity of Ped1 differs from that of canonical DNases in several respects. Unlike DNase I, Ped1 does not completely degrade DNA into mononucleotides under the experimental conditions tested (Fig. 1D). This incomplete digestion pattern is also observed in other newly identified DNases, whether of bacterial or phage origin (Song et al, 2021; Tuck et al, 2024). This observation suggests site-limited cleavage rather than nonspecific degradation, consistent with the finding that Ped1 introduces nicks into supercoiled DNA to produce open-circular and linear products. This “nicking-only” behavior is distinct from that of canonical endonucleases and may allow controlled, localized DNA damage while preserving host resources. Despite the neutral-to-acidic nature of its active pocket in the apo crystal structure, precedents such as SPP1 G2P and human RNase H1 demonstrate that substrate recruitment can rely on shape complementarity or catalytic Mg²⁺ coordination rather than a pre-formed electropositive groove (Smits et al, 2009; Yang et al, 2006). Moreover, the monomeric apo structure likely reflects a latent or auto-inhibited state that achieves catalytic competence upon nucleic acid binding, which is worthy of further in-depth exploration. The strict conservation of this novel DNase among *Pseudomonas* phages suggests that it confers a selective advantage (Fig. 6A,B), likely by enhancing host takeover through its dual nuclease and DnaB-inhibitory activities. Crucially, by integrating the nuclease activity with the physical engagement and inhibition of host DnaB helicase, Ped1 exemplifies a sophisticated viral mechanism for host takeover. Together, these structural and evolutionary features position Ped1 as a founding member of a new nuclease family and reveal a previously undescribed mechanism of host subversion.

To further corroborate the physiological relevance of Ped1-mediated DnaB inhibition we examined the *ter/ori* ratio in vivo as a surrogate marker of replication fork progression. Expression of Ped1 led to a decreased CT(*ori/ter*) ratio, indicating replication fork stalling and impaired replication termination. By contrast, the DnaB-binding-deficient mutant Ped1^Y69A/Y71A^ failed to decrease the CT(*ori/ter*) ratio (Fig. 3F). This observation provides direct in vivo evidence that Ped1 compromises host DNA replication specifically through its interaction with DnaB. Consistently in vitro helicase assays demonstrated that the catalytically inactive but binding-competent variant Ped1^S32L/W58L^ inhibited DnaB unwinding activity in a dose-dependent manner (Figs. 3E and  EV4F,G) whereas the binding-deficient mutants showed no bacterial inhibition effect (Figs. 3C,D and EV4A). Collectively, these data indicate that Ped1 binding to DnaB perturbs its helicase function, thereby impairing replication fork progression. This mechanistic defect likely accounts for the reduced lytic activity of PaoP5Δ*ped1*, as delayed replication collapse would compromise efficient phage‑mediated lysis. Regarding the biochemical basis of this unwinding arrest, mass photometry of the purified DnaB–Ped1 complex resolved three populations (Appendix Fig. S2F). The dominant species (329 ± 58 kDa; 70% of counts) corresponds to a DnaB hexamer bound to a single Ped1 molecule (6×52.7 + 9.5 ≈ 326 kDa), whereas the minor lower-mass population (117 ± 39 kDa; 19%) is consistent with Ped1 bound to a sub-hexameric form of DnaB, and the minor higher-mass population (614 ± 99 kDa; 9%) with a dimer of the Ped1-DnaB hexamer complex. Even though the compositional heterogeneity, together with peak widths (σ) that are large relative to the mass of a single Ped1 molecule (~ 9.5 kDa), preclude assigning a single precise DnaB: Ped1 stoichiometry, these data indicate that Ped1 likely doesn’t inhibit DnaB by promoting dissociation of its hexameric assembly, but instead arrests unwinding while bound to the intact hexamer. Consistently, in vitro unwinding assays using independently purified proteins showed that the catalytically inactive but binding-competent variant Ped1^S32L/W58L^ could significantly inhibit DnaB’s helicase activity upon co-incubation (Fig. 3C–E).

The temporal expression profile of *dnaB* during PaoP5 infection provides a compelling in vivo context for this conflict. The increase in *dnaB* transcription during the first 8 min post-infection, which was markedly attenuated in the Δ*ped1* mutant, highlights Ped1 as a primary driver of host physiological remodeling (Fig. 5A,B). This induction likely represents a classical SOS-like stress response where the host, detecting Ped1-mediated DNA damage, attempts to replenish its replication machinery (Fig. 5C,D). However, this transcriptional upregulation creates a trap: newly synthesized DnaB is immediately sequestered and neutralized by accumulating Ped1. By hijacking host resources in this way, Ped1 effectively diverts them into a futile defense, ensuring host subversion. Although Ped1 is dispensable for the lytic cycle under standard laboratory conditions, it contributes to early replication efficiency and overall lytic activity (Fig. EV5A–E). This contribution likely confers a significant advantage under more competitive or stressful environments. The attenuated lytic activity of PaoP5Δ*ped1* across multiple MOIs and its reduced protective efficacy in vivo (Fig. EV5A–F) indicate that Ped1 acts as an accessory effector that enhances lysis efficiency and phage fitness in physiologically relevant settings, even though it is dispensable under laboratory conditions. This phenotype distinguishes Ped1 from other well-characterized phage-encoded nucleases, such as T4 gp17 and endonuclease II, which are strictly required for phage DNA replication because they generate nucleotides through processive degradation of host DNA (Alam et al, 2008; Lagerback et al, 2009). In contrast, Ped1 is not essential for phage genome replication. Instead, it accelerates host metabolic collapse via a dual-action mechanism: degrading genomic DNA and inhibiting DnaB helicase. This reduces the window for host SOS-mediated recovery. The strict conservation of Ped1 among *Pseudomonas* phages suggests that this accessory function provides a selective advantage in competitive environments, where rapid and efficient lysis is critical for successful proliferation.

In summary, our integrated structural, biochemical, and functional analyses of Ped1 not only expand the known universe of nuclease folds but also unveil a master coordinator of host physiology, providing a textbook example of molecular co-evolution. By simultaneously targeting genomic integrity and the conserved DnaB helicase, the functionally redundant Ped1 employs a synergistic ‘two-hit’ strategy that effectively cripples bacterial viability. The reciprocal antagonism between Ped1 and DnaB exemplifies a dynamic molecular conflict in which a core host replicative machine is co-opted as both a defense and a vulnerability. Although host cells mobilize DnaB to counteract Ped1‑mediated toxicity, this response is ultimately insufficient to prevent lysis, as Ped1’s non‑essential role enables it to function as a functional mimic that diverts host resources toward a futile defense. This mechanism, together with its strict conservation among *Pseudomonas* phages, expands our understanding of phage–host interactions and provides a promising blueprint for developing phage-derived antimicrobial strategies against multidrug-resistant *P. aeruginosa*.

## Methods

**Table:** Reagents and tools table

Reagent/resource	Reference or source	Identifier or catalog number
**Experimental models**		
List of *P. aeruginosa* and *E. coli* strains	This paper	Dataset EV5
List of *Pseudomonas aeruginosa* phage PaoP5	This paper	Dataset EV5
**Recombinant DNA**		
List of plasmids	This paper	Dataset EV5
**Antibodies**		
Mouse anti DDDDK-Tag mAb	Abclonal	AE005
Mouse anti-His-Tag mAb	Abclonal	AE003
Mouse anti-GST-Tag mAb	Abclonal	AE001
HRP-conjugated Goat anti-Mouse IgG (H + L)	Abclonal	AS003
Purified anti-*E. coli* RNA Polymerase α Antibody	BioLegend	663104
**Oligonucleotides and other sequence-based reagents**		
oligonucleotide sequence information	This paper	Dataset EV6
**Chemicals, enzymes, and other reagents**		
IPTG (Isopropyl β-D-1-thiogalactopyranoside)	Sigma-Aldrich	I6758
L-Arabinose	Sigma-Aldrich	A3256
Carbenicillin	Sigma-Aldrich	C1389
Gentamicin	Sigma-Aldrich	G1397
Tetracycline	Sigma-Aldrich	T7660
Kanamycin	Sigma-Aldrich	K4000
DNase I	TransGen Biotech	GD201-01
DNase I reaction buffer	TransGen Biotech	GD201-01
HiTrap Desalting column	Cytiva	17546508
HisTrap HP column	Cytiva	11000458
GSTrap HP column	Cytiva	28401747
Superdex 200 Increase 10/300 GL column	Cytiva	17546508
SYBR Green nucleic acid gel stain	Tsingke Biotech	N/A
One Step TUNEL Apoptosis Assay Kit	Beyotime	C1086
DAPI (4′,6-diamidino-2-phenylindole)	Sigma-Aldrich	D9542
PURExpress^®^ In Vitro Protein Synthesis Kit	New England Biolabs	E6800S
Murine RNase Inhibitor	New England Biolabs	M0314
SYBR Green PCR Master Mix	abm	G891
2×Es Taq MasterMix (Dye)	cwbio	CW0690S (1 ml)
PrimeSTAR^®^ Max DNA Polymerase	TaKaRa	R045Q
RNAprep Pure Cell/Bacteria Kit	TianGen biotech	DP430
Reverse transcriptase kit	abm	G592
SteadyPure Bacterial Genomic DNA Extraction Kit	Accurate Biology	AG21007
ECL Plus Kit	Cytiva	RPN418-1
PVDF membrane	Millipore	IPVH00010
X-gal (5-Bromo-4-chloro-3-indolyl β-D-galactopyranoside)	Sigma-Aldrich	B4252
ONPG (o-Nitrophenyl β-D-galactopyranoside)	Sigma-Aldrich	N1127
QuickMutation™ Random Mutagenesis Kit	Beyotime	D0219S
PreScission Protease	Cytiva	27084301
anti-Flag M2 magnetic beads	Sigma	M8823
BiodyneTM B Nylon membrane	Thermo Scientific	77016
Silver Staining Kit	Beyotime	P0017S
Glutathione beads	Beyotime	P2258
**Software**		
Fiji ImageJ	NIH	https://imagej.net/
GraphPad Prism	GraphPad Software	Version 8.0 or later
MEGA 7	MEGA software	https://www.megasoftware.net/
PyMOL	Schrödinger	Version 2.5 or later (with CavitOmiX plugin)
XDS	Max Planck Institute	Version Jan 2020
aimless	CCP4i v8.0	https://www.ccp4.ac.uk/download/index.php#os=windows
COOT 0.9.8.96 EL	CCP4i v8.0	https://www.ccp4.ac.uk/download/index.php#os=windows
phenix.refine	Phenix 1.19	https://phenix-online.org/download
HADDOCK	Utrecht University	https://wenmr.science.uu.nl/haddock2.4/
Tanon 5200 Image analysis system	Tanon Technologies	
**Other**		
NanoDrop spectrophotometer	Thermo Fisher Scientific	Model: NanoDrop 2000
Leica TCS SP8 microscope system	Leica	N/A
ÄKTA pure	GE Healthcare/Cytiv	29018224

### Bacterial strains and growth conditions

*P. aeruginosa*, *E. coli* strains, and plasmids used in the study are listed in Dataset EV5. Bacterial strains were grown in LB broth with shaking at 220 rpm at 37 °C. For plasmid maintenance, antibiotics were used at the following concentrations where appropriate: for *E. coli*, carbenicillin at 50 μg/mL, gentamicin at 15 μg/mL, and tetracycline at 10 μg/mL; for *P. aeruginosa*, gentamicin at 50 μg/mL, tetracycline at 150 μg/mL, and carbenicillin at 300 μg/mL in LB.

### Plasmids construction

The primers used in this study are listed in Dataset EV6. To obtain expression plasmids, the fragment of phage PaoP5 *ped1* open reading frame was amplified by PCR using the corresponding primers. Then the DNA fragments and vector plasmids were digested with the indicated restriction enzyme and cloned into pMMB67HE, pME6032, pET28a-*ped1*, or pGEX-6p-1 to yield the corresponding plasmid derivatives. Plasmids pET28a-*dnaB* and pHerd-*dnaB* were constructed using the same method as the *P. aeruginosa* genomic DNA. The bacterial two-hybrid system plasmids pUT18C-*ped1* and pKT25-*dnaB* were constructed in a similar manner.

Site-directed mutagenesis plasmids pGEX-6p-1-*ped1*^*S32L*^, pGEX-6p-1-*ped1*^*W58L*^, pGEX-6p-1-*ped1*^*S32L/W58L*^, and pGEX-6p-1-*ped1*^*Y69A/Y71A*^ were constructed by overlap PCR as described previously (Wang et al, 2015). Briefly, DNA of mutant *ped1*^*S32L*^ was amplified by two rounds of PCR. Primer pairs *ped1*-S/*ped1*^*S32L*^-AS and *ped1*^*S32L*^-S/*ped1*^*S32L*^-A were used to amplify segments 1 and 2, respectively. The second round of PCR was carried out by *ped1*-S/A as primer pair, while fragment 1 and fragment 2 as templates to obtain the *ped1*^*S32L*^ fragment. The DNA fragment was digested and cloned into a similar digested pGEX-6p-1 to produce pGEX-6p-1-*ped1*^*S32L*^. Plasmids pGEX-6p-1-*ped1*^*W58L*^ and pGEX-6p-1-*ped1*^*S32L/W58L*^ were constructed in a similar manner.

To construct the pME6032-*ped1*-mCherry plasmid, the *ped1* fragment without the stop codon TAA was amplified using primers *ped1*-*mCherry*-S1/AS1, while the *mCherry* fragment was amplified using primers *ped1*-*mCherry*-S2/AS2. Subsequently, the *ped1*-mCherry fragment was amplified using primers *ped1*-*mCherry*-S1/AS2 by overlap PCR, and then inserted into pME6032 to generate pME6032-*ped1*-mCherry.

### Growth inhibition assay

PAO1 cells harboring the indicated plasmids were cultured in LB medium at 37 °C until reaching the stationary phase. The density of the cell cultures was adjusted to OD_600_ = 1.0. Tenfold serial dilutions were prepared in LB medium, and 2 μl of this cell dilution were spotted onto LB agar plates containing the indicated concentrations of IPTG. The bacterial plates were examined after incubating at 37 °C for 24 h. To obtain growth curves, overnight cultures of cells were diluted 100-fold into LB medium containing the indicated concentrations of IPTG. Growth was monitored by measuring the optical density every 2 h over a 12-h period at 37 °C.

### Microscopy

For cell length analysis, the indicated PAO1 cells expressing Ped1 or Ped1^S32L/W58L^ with or without DnaB were cultured in LB medium with or without IPTG and/or L-Arabinose. At the indicated time points, the cells were harvested and resuspended in PBS buffer. The cells were placed on pad slides and observed using the Leica TCS SP8 microscope system (Leica). Cell length and images were processed with Fiji ImageJ.

### Protein purification

To express and purify His_6_-tagged recombinant proteins, pET28a or pET22b was transformed into *E. coli* BL21(DE3). The overnight culture was sub-cultured in 1 L fresh LB medium at 37 °C until OD_600_ to 0.6, then the cells were induced with 0.5 mM IPTG for another 16 h at 16 °C. Cells were collected by centrifugation and resuspended in His Buffer A (10 mM Tris, 500 mM NaCl, and 10% Glycerol, pH 7.5), and sonicated on ice. After sonication, the supernatant was passed through a HisTrap HP column (Cytiva), and impurities were removed using 10% His Buffer B (50 mM Tris-HCl, 500 mM NaCl, 500 mM imidazole, pH 7.5) and 90% His Buffer A with the ÄKTA purification system. The target protein was eluted with a gradient of 100% His Buffer B. The proteins were desalted with His Buffer C (50 mM Tris-HCl, 200 mM NaCl, pH 7.5) by HiTrap Desalting column (Cytiva). The resulting proteins were analyzed by SDS–PAGE and stored at −80 °C until use.

To express and purify GST-tagged recombinant proteins, pGEX-6p-1 plasmids derivative were used, and the corresponding protein production cells were collected by centrifugation. Then the cells were resuspended in GST Buffer A (10 mM Tris-HCl, 300 mM NaCl, pH 7.5) and sonicated on ice. The centrifuged supernatant was passed through a GSTrap HP column (Cytiva), the Impurities were removed using GST Buffer A in an ÄKTA system, and the GST-tagged protein was eluted with GST Buffer B (10 mM Tris-HCl, 300 mM NaCl, 10 mM GSH, pH 7.5). The proteins were desalted with GST Buffer C (10 mM Tris-HCl, 200 mM NaCl, pH 7.5) by HiTrap Desalting column. The resulting proteins were analyzed by SDS–PAGE and stored at -80 °C until use. To obtain the non-tagged Ped1 and variants, the GST tag was removed by incubation with PreScission Protease (Cytiva) for 20 h at 16 °C, and untagged proteins were eluted from the GSTrap HP column with GST Buffer C according to the protocol.

### In vitro nuclease assay

To detect the DNase activity of Ped1, 0.1 mM Ped1 or variants were mixed with 0.3 ng of indicated genomic DNA or plasmid DNA in DNase I reaction buffer. After incubation for 30 or 60 min at 37 °C, the integrity of DNA was analyzed by 1% agarose gel electrophoresis and visualized with SYBR Green (Beijing Tsingke Biotech) by a Tanon 5200 Image analysis system (Tanon Technologies). The RNase activity of Ped1 was assessed in a similar manner using a Tris reaction buffer (50 mM Tris-HCl, 200 mM NaCl, pH 7.5).

To detect the DNase activity of Ped1 on single stand DNA, a 52-nt biotin-labeled DNA oligonucleotide was synthesized by Beijing Tsingke Biotech (Dataset EV6). Then 0.1 mM Ped1 was mixed with 5 nM of biotin-labeled DNA in DNase I reaction buffer. After incubation for 60 min at 37 °C, the DNA sample was resolved by native PAGE by electrophoresis and transferred onto a Biodyne^TM^ B Nylon membrane (Thermo Scientific). The integrity of DNA fragments was visualized by a streptavidin-horseradish peroxidase conjugate and ECL Plus Kit (Cytiva) according to the manufacturer’s protocol.

### In vivo TUNEL assay

TUNEL assay was carried out by one Step TUNEL Apoptosis Assay Kit (Beyotime) according to the manufacturer’s protocol with minor modification. PAO1 cells harboring the pME6032-*ped1* or empty pME6032 plasmids were cultured at 37 °C with 1 mM IPTG induction until reaching the stationary phase. A 1 mL (10^9^) aliquot of cell culture was centrifuged to collect the cells. The resulting cells were washed with PBS and fixed in 4% paraformaldehyde for 30 min and incubated at 37 °C for 5 min in PBS buffer containing 0.3% Triton X-100. After washing twice with PBS, 50 μL of TUNEL solution was added, and the cells were incubated at 37 °C for 60 min in the dark. Then, the cells were washed twice with PBS and suspended in 25 μL PBS and examined with a fluorescence microscope.

### DAPI staining

PAO1 cells harboring the pME6032-*ped1* or empty pME6032 plasmids were cultured at 37 °C with 1 mM IPTG induction until reaching the stationary phase. A 1 mL/10^9^ aliquot of cell culture was centrifuged to collect the cells. The resulting cells were washed with PBS and resuspended in 50 μL PBS with 15 μg/ml DAPI (Sigma-Aldrich). After incubation at 37 °C for 60 min from light, the cells were washed twice with PBS. The cells were resuspended in 50 μL of PBS buffer and examined with a fluorescence microscope.

### Crystallization, data collection, processing, model building, and refinement

The recombinant fusion protein Ped1 with an N-terminal hexahistidine tag was expressed exogenously in *E. coli* BL21(DE3). The cells were lysed in a buffer containing 50 mM HEPES and 500 mM NaCl at pH 6.8. The target protein was purified from the supernatant using Ni-NTA affinity chromatography, followed by size-exclusion chromatography (Superdex 75 Increase) with a buffer composed of 20 mM HEPES and 300 mM NaCl at pH 6.8. Protein purity was assessed via SDS–PAGE and quantified using a NanoDrop spectrophotometer (Thermo Scientific). Initial screening for crystallization was conducted at a protein concentration of 8 mg/mL using commercial kits, which include Index^TM^, Proplex^TM^, Crystal Screen^TM^, PEG Ion^TM^, PACT^TM^, and JCSG^TM^ (Hampton Research). Crystals suitable for diffraction were grown in a solution containing (w/v) 25% PEG3350, 0.1 M Bis-Tris pH 6.5 employing the sitting-drop vapor diffusion method at 16 °C. The crystals were cryoprotected by briefly soaking in the reservoir solution supplemented with 20% glycerol. X-ray diffraction data were collected at the Shanghai Synchrotron Radiation Facility (SSRF) beamline BL19u1, utilizing a wavelength of 0.97923 Å and the small-angle oscillation method with a 0.5° oscillation angle, yielding a total of 720 images in total. The data were processed with XDS and autoPROC for peak search, refinement, and integration, followed by scaling using the AIMLESS tool integrated into CCP4i. The phase problem was addressed through molecular replacement (MR) using PHASER within the PHENIX suite. The search model was derived from fragments predicted by the PyMOL plugin CavitOmiX. The initial model was built automatically using AutoBuild, and subsequent refinement was performed iteratively with REFMAC5 and the PHENIX.refine module. Model quality was evaluated using MolProbity (Comprehensive Validation) in PHENIX. Data collection and refinement statistics are summarized in Dataset EV7.

### Molecular docking

To further investigate the interactions between the Ped1 protein and double-stranded DNA (dsDNA), molecular docking simulations were conducted using the HADDOCK server. The three-dimensional structure of Ped1 was obtained from crystallization trials made in this study, while the initial atom coordinates of dsDNA were generated using AlphaFold. The docking procedure involved rigid-body docking followed by semi-flexible refinement, utilizing the HADDOCK refinement module with default parameters. The top-ranked complexes were subjected to further analysis and validation using the PDBePISA server to assess the stability and reliability of the predicted interactions.

### Co-immunoprecipitation assay

In vivo co-immunoprecipitation analysis was carried out with anti-Flag M2 magnetic beads (Sigma) as described previously with minor modification (Han et al, 2019). Briefly, PAO1 cells harboring the pMMB67H-*ped1*-Flag or empty vector plasmid were cultured in 100 mL LB medium with 1 mM IPTG at 37 °C. Cells were harvested by centrifugation and resuspended in Co-IP lysis buffer (20 mM Tris, 150 mM NaCl, 0.1% Triton X-100, pH 8.0) and sonicated on ice. The centrifuged supernatant was incubated with 20 μL anti-Flag M2 magnetic beads at 4 °C for 4 h, followed by washing five times with Co-IP wash buffer (20 mM Tris, 500 mM NaCl, 0.1% Triton X-100, pH 8.0) to remove unbound proteins. Then the beads-coated protein was dissolved in 100 μL SDS loading buffer, followed by SDS–PAGE, and the proteins were visualized by silver staining (Beyotime). The individual protein band retained by beads coated with Ped1-Flag, but not vector control, was excised and analyzed by LC-MS/MS.

### Size-exclusion chromatography

Size-exclusion chromatography analysis was carried out using ÄKTA purification system with Superdex 200 increase 10/300 GL column (Sigma-Aldrich). The purified GST-Ped1 and DnaB-His_6_ were used to detect the interaction between Ped1 and DnaB. 0.2 mM of GST-Ped1 and 0.2 mM DnaB-His_6_ were mixed in 500 μL GST buffer A. After overnight incubation at 4 °C, 450 μL of the mixture was passed through a Superdex 200 increase 10/300 GL with GST buffer A. Proteins were collected according to the elution peaks, and the collected fractions were analyzed by SDS–PAGE electrophoresis followed by Coomassie brilliant blue. GST-Ped1 or DnaB-His_6_ alone was an individual control.

### Bacterial two-hybrid assay

Bacterial two-hybrid complementation assays were carried out as previously described (Karimova et al, 1998). To specify, pKT25-*dnaB*/pUT18c-*ped1* or indicated control plasmids were co-transformed into *E. coli* BTH101 and spread on LB agar plates with 1 mM IPTG and 40 μg/mL X-gal. The agar plates were incubated for 24 h at 30 °C. To quantify the interaction of protein pairs, the β-galactosidase activity was assayed using ONPG as the substrate.

### GST pull-down assay

To demonstrate the interaction between Ped1 and DnaB in vitro, GST pull-down assay with the indicated proteins was performed as described previously (Wang et al, 2020). Briefly, 100 μg of purified GST or an equal amount of GST-Ped1 protein were mixed with pre-washed glutathione beads slurry (Beyotime) in GST buffer A. After incubating at 4 °C for 2 h, the unbound proteins were washed away with GST buffer A. Then, the beads were mixed with 20 μg of DnaB-His_6_ protein and incubated at 4 °C for another 4 h. After incubation, the beads were washed three times with GST Buffer A (10 mM Tris-HCl, 300 mM NaCl, pH 7.5) to remove unbound proteins. Subsequently, the GST beads retained proteins were detected by immunoblot analysis after SDS–PAGE using anti-His and anti-GST antibodies (Abclonal).

### Western blot analysis

For western blots, protein samples were separated by SDS–PAGE and transferred to a polyvinylidene difluoride (PVDF) membrane (Millipore). After blocking with 5% milk, the membrane was sequentially incubated with primary antibodies and horseradish peroxidase-conjugated secondary antibodies in TBST buffer. The target protein signal was detected using the ECL Plus reagent kit (Cytiva) according to the manufacturer’s instructions by a Tanon 5200 Image analysis system (Tanon Technologies).

### Isothermal titration calorimetry assay (ITC)

The binding of Ped1 to DnaB was characterized by isothermal titration calorimetry using a MicroCal PEAQ-ITC instrument (Malvern Panalytical) at 25 °C with a reference power of 5. The sample cell was loaded with DnaB at a final concentration of 50 μM, and the injection syringe contained Ped1 at 300 μM; both proteins were prepared in the buffer containing 20 mM HEPES 150 mM NaCl (pH 7.5) to minimize heats of dilution. The titration comprised 19 injections in total: an initial 0.4 μL injection delivered over 0.8 s, followed by 18 injections of 2 μL each delivered over 4 s, with an interval of 120 s between consecutive injections to allow the thermal power signal to return to baseline. The first injection (0.4 μL) was excluded from the analysis to correct for ligand diffusion across the syringe tip during the pre-titration equilibration period. The heat evolved by each injection was integrated, normalized per mole of injectant, and plotted against the molar ratio of Ped1 to DnaB. The resulting binding isotherm was fit to a one-site binding model to determine the dissociation constant Kd using the MicroCal PEAQ-ITC Analysis Software (Malvern Panalytical).

### Error-prone PCR screening

To construct the pKT25-*ped1* error-prone PCR library, the *ped1* gene was first amplified using the QuickMutation™ Random Mutagenesis Kit (Beyotime). The resulting PCR product was purified and subsequently cloned into the pKT25 vector using the same restriction enzyme digestion sites. The ligation mixture was then transformed into *E. coli* BTH101 cells harboring pUT18C-*dnaB* via electroporation. Transformants were selected on LB agar plates supplemented with 1 mM IPTG and 40 µg/mL X‑gal. White colonies were picked, and the specific mutation sites within the *ped1* gene were identified by DNA sequencing.

### Fork unwinding assay

DNA fork unwinding assay for DnaB was performed according to the methods by Kaplan and Steitz (Kaplan and Steitz, 1999). Biotin-labeled Fork DNA1 and Fork DNA2 were synthesized by Beijing Tsingke Biotech (Dataset EV6). Then, the same amount of two DNA fragments was annealed by heating to 95 °C for 5 min followed by slow cooling to room temperature to form double-stranded forked DNA. Then 2 μM of the purified DnaB, or co-purified DnaB-Ped1 and DnaB-Ped1^S32L/W58L^ complex, were added to a 10 µl reaction system containing 50 nM forked DNA in reaction buffer (50 mM HEPES, 10 mM DTT, 10 mM MgAc2, 2 mM ATP, and 0.2 mg/ml BSA). After incubation at 37 °C for 30 min, 2.5 µL of termination buffer (100 mM Tris-HCl, 200 mM EDTA, 0.5% SDS, pH 7.5) was added. Then, 2.5 µL of color development solution (30% glycerol, 0.25% bromophenol blue) was added and resolved by native PAGE by electrophoresis, followed by transfer onto a Biodyne^TM^ B Nylon membrane (Thermo Scientific). Biotin-labeled DNA was visualized by a streptavidin-horseradish peroxidase conjugate and ECL Plus Kit (Cytiva) according to the manufacturer’s protocol.

### RNA-seq and data analysis

For transcriptome sequencing, PAO1 cells harboring the pME6032-*ped1* or empty pME6032 plasmids were cultured at 37 °C with 1 mM IPTG induction until reaching the stationary phase. Cells from a 1 mL aliquot of culture (~10⁹ cells) were collected by centrifugation. The total RNA was extracted from the collected cells using the RNAprep Pure Cell/Bacteria Kit (TianGen biotech) and the DNase I Kit (TianGen biotech) according to the manufacturer’s protocol. After removing ribosomal RNA by MICROBExpress™ Kit (Invitrogen), mRNA was used to generate the cDNA library according to the NEBNext® Ultra™ II RNA Library Prep Kit for Illumina (NEB). Then, cDNA libraries were sequenced on an Illumina HiSeq 2000 system. Subsequently, the clean reads of the strains were mapped to the reference genome of *P. aeruginosa* using BWA alignment (Li and Durbin, 2009). RPKM was used to normalize the expression level of genes by RESM software (Li and Dewey, 2011; Mortazavi et al, 2008), Differential expression genes (DEGs) were identified using DESeq2, statistical significance was assessed using the Wald test, and *P* values were adjusted for multiple testing using the Benjamini–Hochberg procedure to control the false discovery rate (FDR) (*P* < 0.05 and |log_2_ fold change | > 1) (Kanehisa et al, 2008). KEGG pathway enrichment analysis of DEGs was also performed to identify significantly enriched metabolic pathways using the KEGG Orthology (KO) system. All sequencing data have been uploaded to BioProject under accession number PRJNA1256806.

### Quantitative real-time PCR

Total RNA was isolated from bacterial samples as described for RNA-seq analysis above, then cDNA was synthesized from RNA samples using a reverse transcriptase kit (abm), then the quantitative real-time PCR was performed using SYBR Green PCR Master Mix (abm) with primers listed in Dataset EV6. The relative mRNA levels were normalized to *rpsL* as an internal control, and the relative changes in gene expression among them were calculated using the relative quantification method.

### Sequence analysis and phylogeny of Ped1

The protein sequence of PaoP5_157 was accessible in the National Center for Biotechnology Information (NCBI) databank (www.ncbi.nlm.nih.gov/protein) under accession number ALT58436. A total of 24 protein sequences were found by using BLASTp search against the NCBI non-redundant protein database with a sequence identity threshold of ≥30% (QE325 gp108 as an outgroup), followed by manual curation if necessary. These protein sequences were aligned using the CLUSTALW program (www.clustal.org), and the corresponding tree was inferred by MEGA version 7 (www.megasoftware.net) using the neighbor-joining method under the Jones-Taylor-Thornton model and with branch support assessed by 1000 bootstrap replicates.

### Mass photometry analysis of the DnaB–Ped1 complex

Mass photometry measurements were conducted on a Refeyn OneMP mass photometer (England), which determines the mass of individual biomolecules in solution via interferometric scattering microscopy (Young et al, 2018). Prior to each measurement, all buffers and samples were equilibrated to room temperature to minimize thermally induced focal drift. A clean glass coverslip (24 × 50 mm²) was placed on the sample stage together with a self-adhesive silicone CultureWell gasket, and immersion oil was applied to the objective lens with care taken to exclude air bubbles, which could otherwise disturb the autofocus ring and compromise measurement stability. A 16‑µL droplet of measurement buffer (20 mM HEPES, 150 mM NaCl, pH 7.5) was deposited into the gasket well and used to locate and lock the focal plane by maximizing image sharpness; a brief buffer‑only movie was recorded to establish the background signal. The DnaB–Ped1 sample was diluted to 0.1 µM in the same buffer, and 2 µL of this diluted sample was introduced into the buffer droplet and mixed thoroughly by repeated pipetting. Data acquisition was performed using AcquireMP software (Refeyn). If the number of detected particle landing events was insufficient (< 500 counts), an additional sample was added incrementally (up to a maximum cumulative volume of 20 µL) to improve counting statistics. Mass calibration was carried out using bovine serum albumin (BSA, 66 kDa) as the reference standard. The recorded movies were analyzed with DiscoverMP software (Refeyn) to generate ratiometric contrast histograms, convert contrast to mass based on the BSA calibration curve, and determine the oligomeric mass distribution of the DnaB–Ped1 complex.

### Knockout of phage genes using the CRISPR-Cas9 system

Phage genes were knocked out using the CRISPR‑Cas9 plasmid pPTCS according to the established method (Le et al, 2024). To delete the *ped1* gene in phage PaoP5, a spacer was generated by annealing primers *ped1*-gRNA-S/AS and ligated into *Eco31* I-digested pPTCS. To construct the recombination template, primer pairs *ped1*-Up-S/AS and *ped1*-Down-S/AS were used to amplify the 250 bp fragments upstream and downstream of the *ped1* gene, respectively. The upstream and downstream fragments were fused by overlap PCR and then cloned into the multiple cloning site of pTCPLS. The resulting plasmid was transformed into PAO1 to generate the PAO1/pTCPLS-Δ*ped1* strain, which was subsequently infected with 10^5^ PFU of phages. Plaques were selected for PCR, and the mutant phage was verified by PCR and sequencing. All primers used are listed in Dataset EV6.

### *Galleria Mellonella* killing assay

The therapeutic efficacy of wild-type PaoP5 and PaoP5Δ*ped1* against PAO1 infection was evaluated using a *Galleria mellonella* larva model as described previously (Yan et al, 2026). Larvae were randomly divided into four groups (*n* = 20 per group) and injected with 10 μL of sterile PBS, PAO1 alone (1 × 10^5^ CFU/larva), PAO1 plus wild-type PaoP5, or PAO1 plus PaoP5Δ*ped1*. For phage-treated groups, phages were administered at an MOI of 0.1 simultaneously with bacterial challenge. Following injection, larvae were incubated at 37 °C in the dark and monitored every 2 h for up to 24 h.

### Transmission electron microscopy

For morphological analysis, phage particles were visualized by transmission electron microscopy (TEM). An aliquot of the phage lysate was deposited onto a carbon-coated copper grid and incubated for 1 min at room temperature. The grid was subsequently negatively stained with 2% phosphotungstic acid for 30 s. Images were acquired using a HT7800 transmission electron microscope (Hitachi, Tokyo, Japan).

### In vitro protein synthesis using the PURExpress^®^ system

In vitro protein synthesis was performed using the PURExpress^®^ In Vitro Protein Synthesis Kit (NEB) according to the manufacturer’s instructions. Each 25-μL reaction contained 10 μL Solution A, 7.5 μL Solution B, 250 ng plasmid DNA template (e.g., pET28a-*ped1*-flag), 20 units of Murine RNase Inhibitor (NEB), and nuclease-free water to volume. Solution B was added last. Reactions were incubated at 37 °C for 2 h and terminated on ice. For SDS–PAGE analysis, 2.5 μL of the reaction product was heated at 100 °C for 3 min and resolved by SDS–PAGE, followed by Coomassie Brilliant Blue staining or western blot.

### Quantitative analysis of genome replication

To assess bacterial DNA replication status, the copy number ratio of the chromosomal origin (*ori*) to terminus (*ter*) was determined by qPCR. The *ori* and *ter* regions of *P. aeruginosa* PAO1 were identified using the online tool Tubic (tubic.org), and specific primers were designed. Only primers with 90–100% efficiency and *R*^2^ > 0.95 were used in Dataset EV6. Genomic DNA was extracted from mid-log-phase cells (OD_600_ = 1) using a Bacterial Genomic DNA Extraction Kit (Accurate Biotechnology) and diluted to 10 ng/μL. Subsequently, quantitative real-time PCR was performed using SYBR Green PCR Master Mix (abm).

## Supplementary information

Appendix

Peer Review File

Dataset EV1

Dataset EV2

Dataset EV3

Dataset EV4

Dataset EV5

Dataset EV6

Dataset EV7

Source data Fig. 1

Source data Fig. 2

Source data Fig. 3

Source data Fig. 4

Source data Fig. 5

Source data Fig. 6

Figure EV1 Source Data

Figure EV2 Source Data

Figure EV3 Source Data

Figure EV4 Source Data

Figure EV5 Source Data

Appendix Figure S1-S6 Source Data

Expanded View Figures

## Data Availability

All sequencing data have been uploaded to BioProject under accession number PRJNA1256806. The source data of this paper are collected in the following database record: biostudies:S-SCDT-10_1038-S44318-026-00924-8.
