# Supplementary material for: A dual-action bacteriophage protein Ped1 degrades DNA and blocks replication in Pseudomonas aeruginosa
Source: EMBO J. 2026 Oct 2;45(21):7541–63. doi: 10.1038/s44318-026-00924-8 (PMC13728149; doi:10.1038/s44318-026-00924-8)
Supplement: Supplementary file 1 [file 44318_2026_924_MOESM1_ESM.pdf]

Appendix for "*A dual-action bacteriophage protein Ped1 degrades DNA and blocks replication in Pseudomonas aeruginosa*"

Table of contents

Appendix Figure S1. *In vivo* visualization and quantification of DNA damage and depletion by Ped1. 2

Appendix Figure S2. Identification of DnaB as a direct binding partner of Ped1. 3

Appendix Figure S3. RT-qPCR analysis of *ped1* and *dnaB* expression at 1, 5, 8, 10 and 15 minutes post-infection. 4

Appendix Figure S4. Validation of transcriptomic data and global host response analysis. 5

Appendix Figure S5. Plaque morphology and one-step growth curve analysis of the PaoP5Δ*ped1* mutant. 6

Appendix Figure S6. Purification of Ped1 and sequence alignment of its homologs. 6

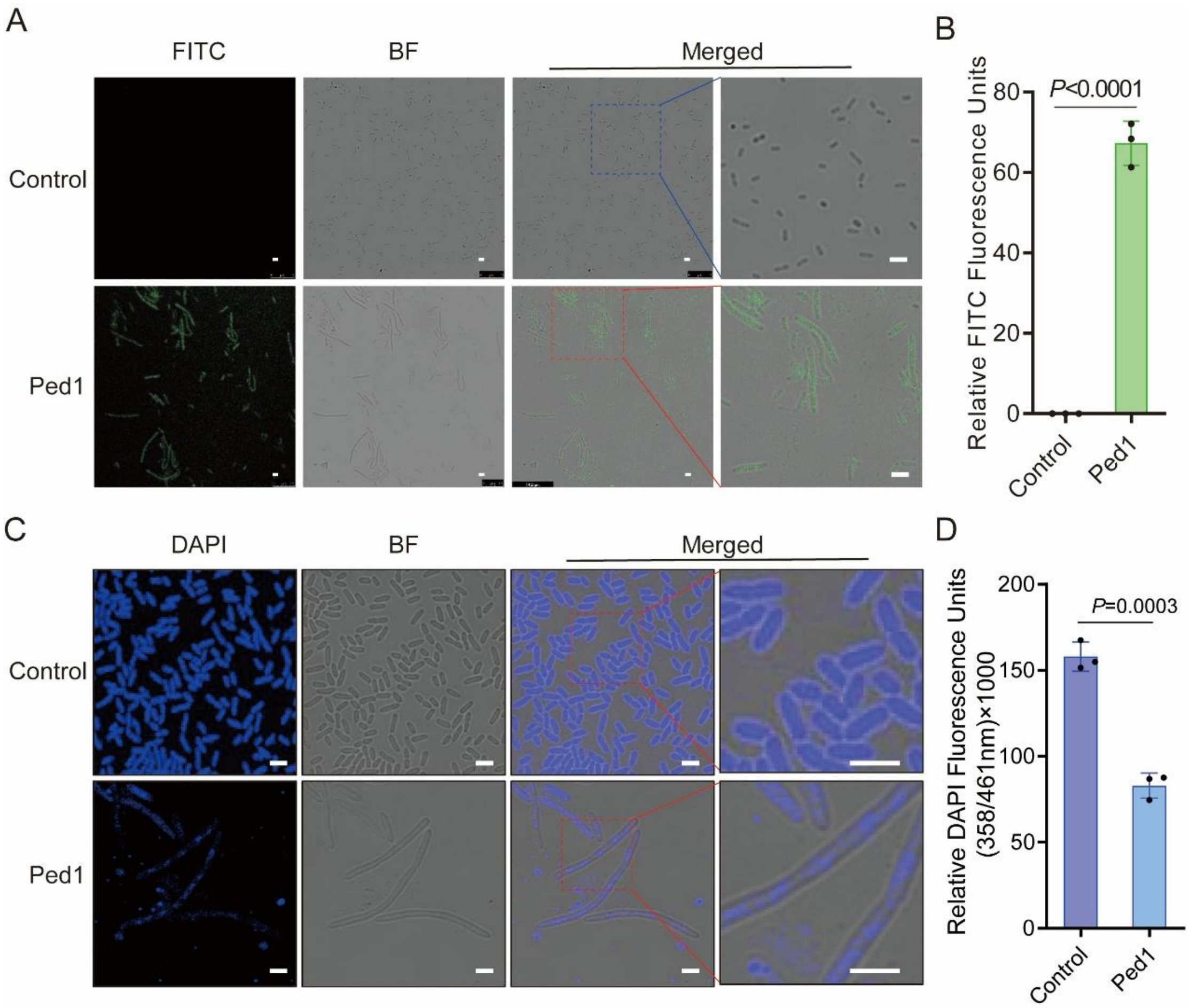

Appendix Figure S1. ***In vivo*** **visualization and quantification of DNA damage and depletion by Ped1.**

(A) TUNEL assay for detecting DNA strand breaks. Representative fluorescence (FITC), bright-field (BF), and merged micrographs of P. aeruginosa PAO1 cells harboring an empty vector (Control) or induced to express Ped1. Intense green fluorescence (FITC) indicates the presence of extensive DNA fragmentation in Ped1-expressing cells. Scale bar, 2.5 μm. **(B)** Quantification of TUNEL signal. Bar graph showing a significant increase in relative FITC fluorescence units in cells producing Ped1 compared to the control group (P < 0.0001). Data represent mean ± SD from three independent experiments. **(C)** DAPI staining for visualizing genomic DNA content. Micrographs showing DAPI-stained genomic DNA (blue) in Control and Ped1-expressing cells. Cells overexpressing Ped1 exhibit visibly diminished DAPI fluorescence, indicating severe intracellular DNA depletion. Scale bar, 2.5 μm. **(D)** Quantification of intracellular DNA content. Bar graph showing approximately a 2-fold reduction in relative DAPI fluorescence units in Ped1-producing cells compared to

vector control cells (P = 0.0003). Data are represented as mean ± SD from three independent experiments (*B, D*). Statistical significance was determined by unpaired *t* test (*B, D*).

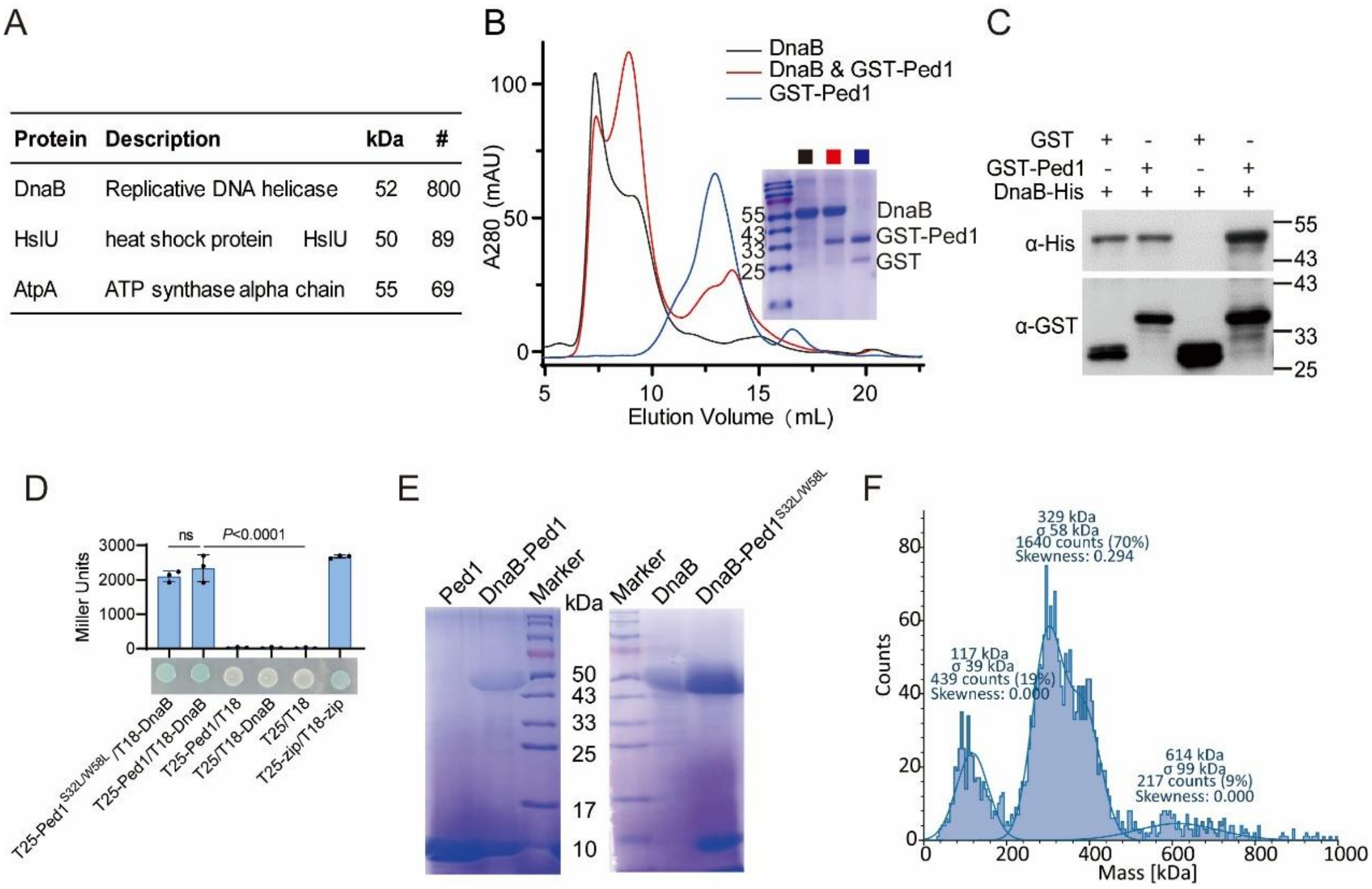

Appendix Figure S2. Identification of DnaB as a direct binding partner of Ped1.

(A) Mass spectrometry identification of Ped1-interacting proteins. A summary table lists the top three host proteins enriched from *P. aeruginosa* PAO1 cell lysates via co-immunoprecipitation with Ped1-Flag. The replicative DNA helicase DnaB was identified with the highest hit counts. The symbol "#" indicates the number of identified peptides **(B)** Size-exclusion chromatography (SEC) analysis of the Ped1-DnaB interaction. Chromatograms for purified DnaB alone (black), GST-Ped1 alone (blue), and a DnaB/GST-Ped1 mixture (red). The significant peak shift for the mixture and the corresponding SDS-PAGE analysis (inset) demonstrate the formation of a stable complex. **(C)** GST pull-down assay confirming direct interaction. Immunoblotting with α-His and α-GST shows that DnaB-His is specifically pulled down by GST-Ped1, but not by the GST-only control. **(D)** Bacterial two-hybrid (BACTH) analysis in cellular context. Interaction between Ped1 and DnaB was quantified by β-galactosidase activity (Miller Units) and visualized on X-gal-containing plates (blue spots). The catalytically inactive Ped1$^{S32L/W58L}$ variant retains its high-affinity binding to DnaB ($P < 0.0001$ vs. controls). **(E)** SDS-PAGE analysis of purified

proteins and complexes. Coomassie-stained gels show the purity of recombinant Ped1, DnaB, and the DnaB-Ped1 (WT and Ped1$^{S32L/W58L}$) complexes used for biochemical assays. **(F)** Mass photometry of the purified DnaB–Ped1 complex. The contrast histogram was fitted with three Gaussian distributions; the mean mass, standard deviation (σ), event counts (percentage of total) and skewness are indicated for each population. The predominant species (329 kDa, 70%) basically corresponds to a DnaB hexamer (~316 kDa) bound to 1 Ped (~9.5 kDa) molecules. Data are representative of three independent experiments (*B, C, E, F*). Data are represented as mean ± SD from three independent experiments. Statistical significance was determined by ordinary one-way ANOVA with Dunnett's multiple comparisons test (*D*).

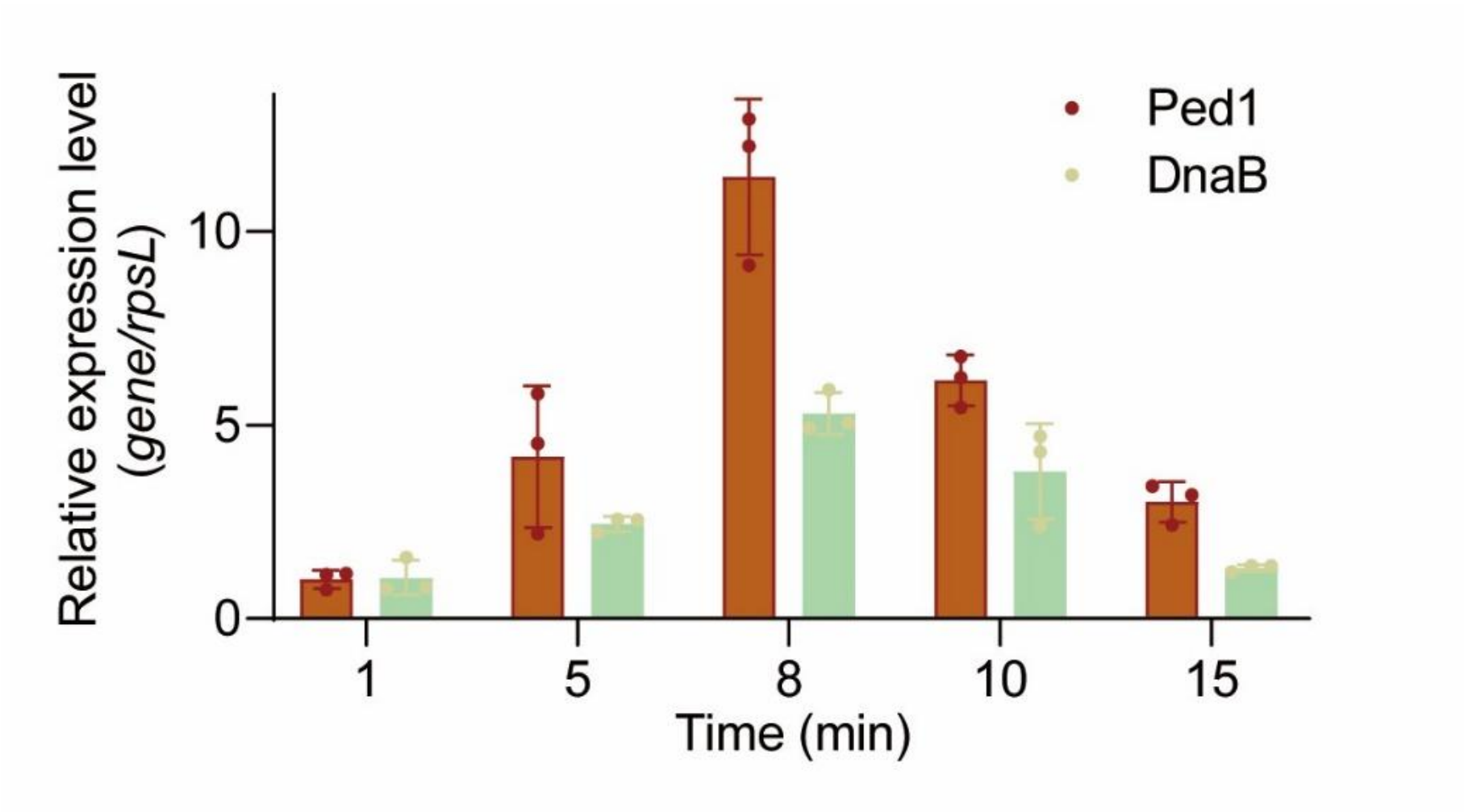

Appendix Figure S3. RT-qPCR analysis of *ped1* and *dnaB* expression at 1, 5, 8, 10 and 15 minutes post-infection.

P. aeruginosa PAO1 was infected with phage PaoP5 (MOI = 10). At indicated time, total RNA was extracted and analyzed by qRT-PCR using specific primers for *ped1, dnaB* and the housekeeping gene *rpsL*. Transcript levels are shown are normalized to *rpsL* at indicated time points. Data are represented as mean ± SD from three independent experiments.

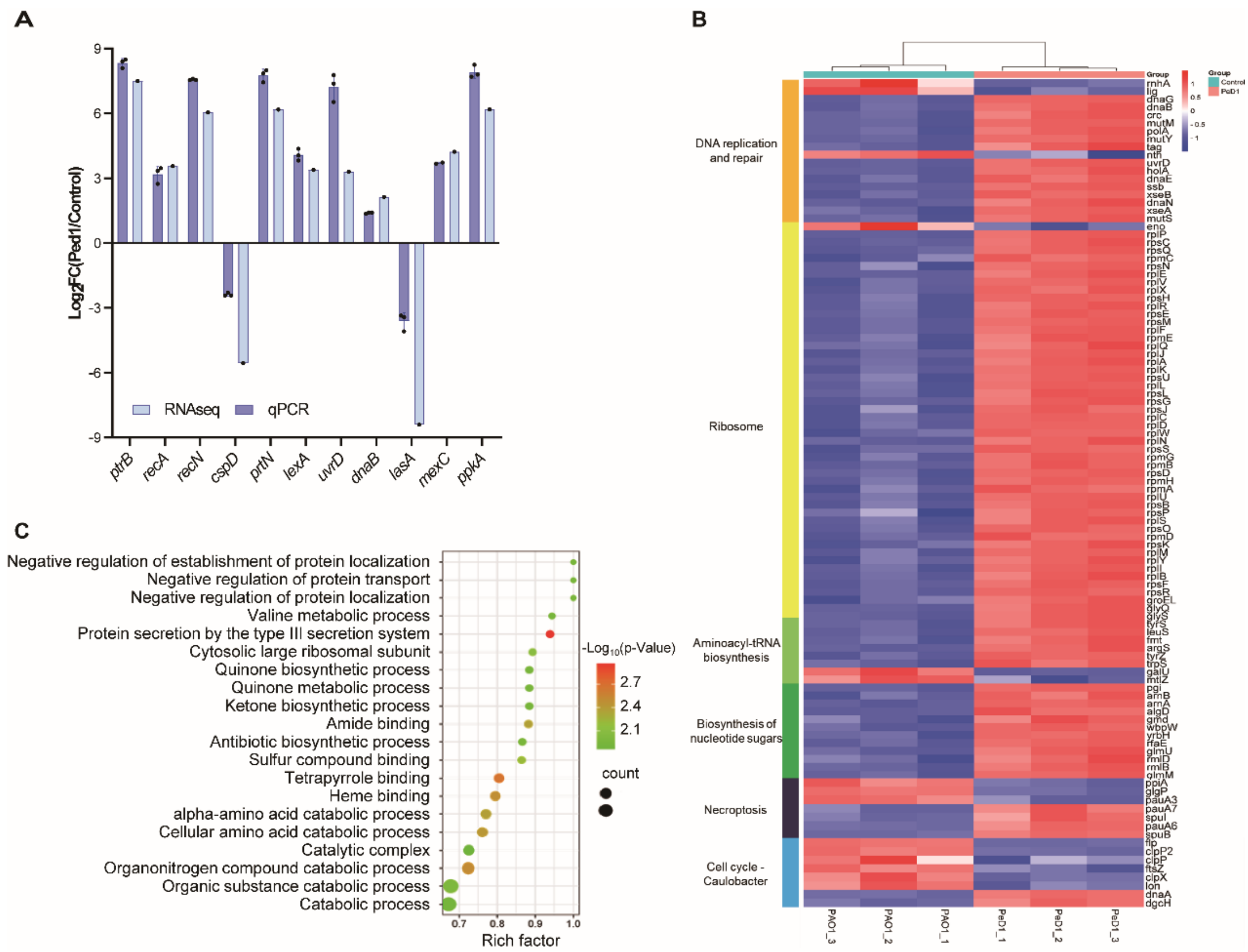

Appendix Figure S4. Validation of transcriptomic data and global host response analysis.

(A) Validation of RNA-seq results by qRT-PCR. Expression of eleven representative genes was measured in three independent repeated experiments. Data are mean ± SD. **(B)** Heatmap of Ped1-regulated core metabolic genes in *P. aeruginosa* PAO1. RPKM (reads per kilobase per million mapped reads) values of selected genes in PAO1 expressing Ped1 (Ped1_1-3) versus empty-vector control (PAO1_1-3). Each column represents one of three biological replicates. Color scale indicates expression levels (blue, low; red, high). Genes are grouped according to their associated metabolic pathways. **(C)** GO enrichment analysis (bubble chart) of differentially expressed genes, showing the top 20 enriched terms (P< 0.0136). Dot size indicates gene count; color represents -$\log_{10}$(P-value). Statistical significance was determined using the Wald test with Benjamini-Hochberg correction for multiple testing (*B, C*).

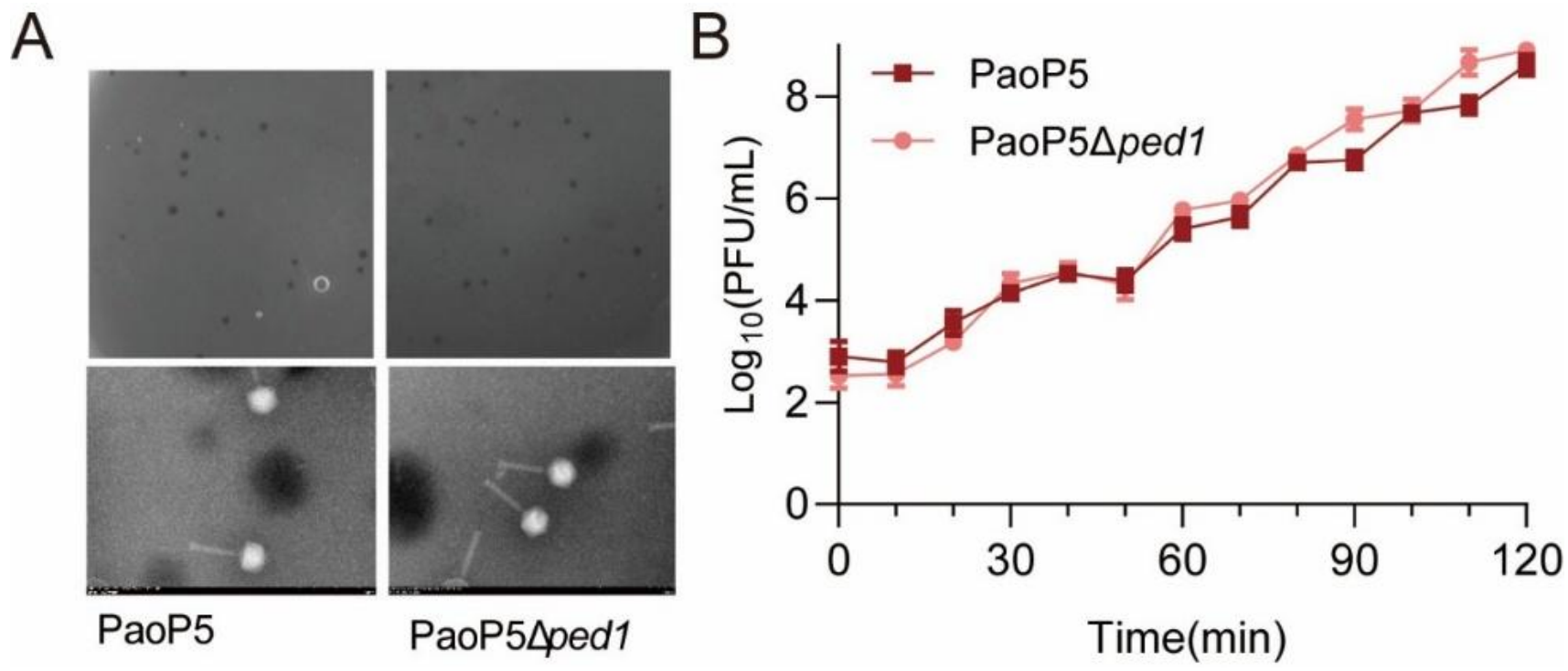

Appendix Figure S5. Plaque morphology and one-step growth curve analysis of the PaoP5Δ*ped1* mutant.

(A) Plaque morphology and scanning electron microscopy (SEM) images of PaoP5 and PaoP5Δ*ped1* are shown. **(B)** One-step growth curve of wild-type PaoP5 and PaoP5Δ*ped1* on PAO1. Data are representative of three independent experiments; error bars represent mean ± SD.

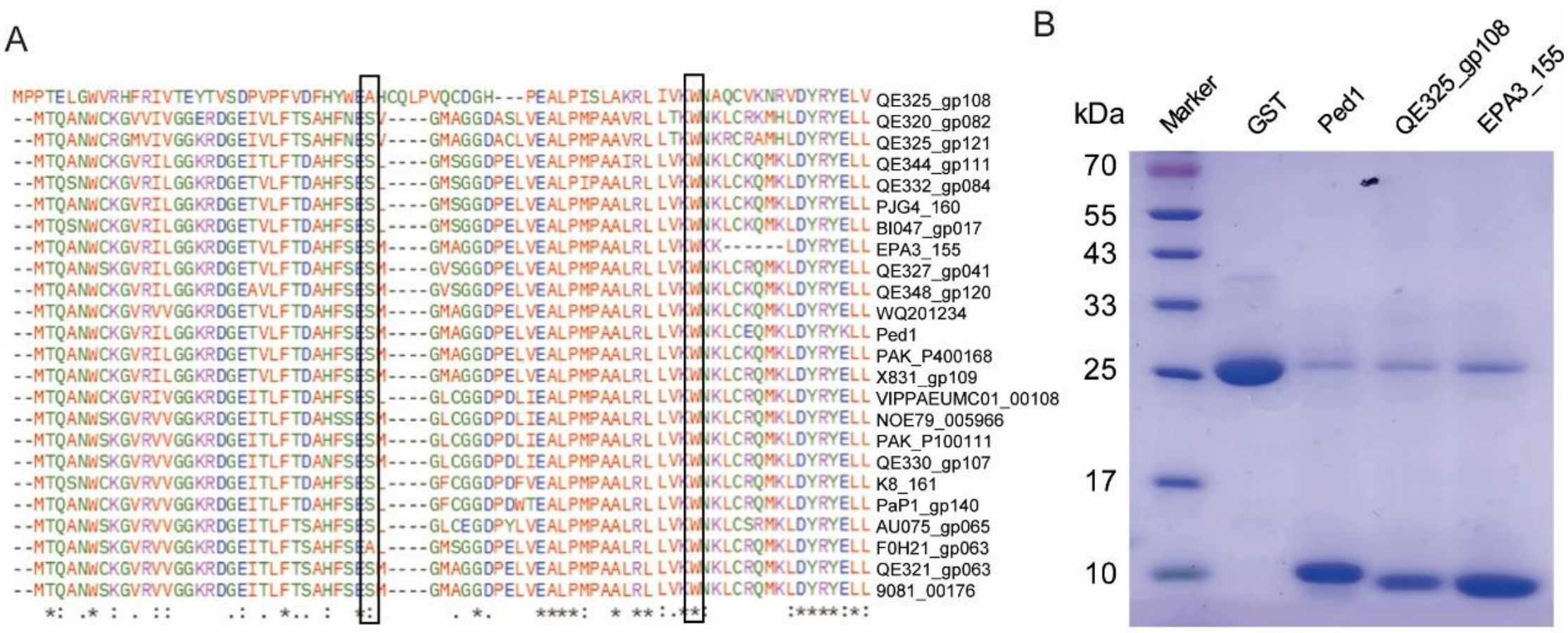

Appendix Figure S6. Purification of Ped1 and sequence alignment of its homologs.

(A) Multiple sequence alignment of Ped1 homologs. **(B)** Purified GST, Ped1, QE325_gp108, and EPA3_155 were separated by SDS-PAGE and stained with Coomassie Blue R-250. Data are representative of three independent experiments.
