# Supplementary material for: A dual-action bacteriophage protein Ped1 degrades DNA and blocks replication in Pseudomonas aeruginosa
Source: EMBO J. 2026 Oct 2;45(21):7541–63. doi: 10.1038/s44318-026-00924-8 (PMC13728149; doi:10.1038/s44318-026-00924-8)
Supplement: Supplementary file 2 [file 44318_2026_924_MOESM22_ESM.pdf]

Expanded View Figures

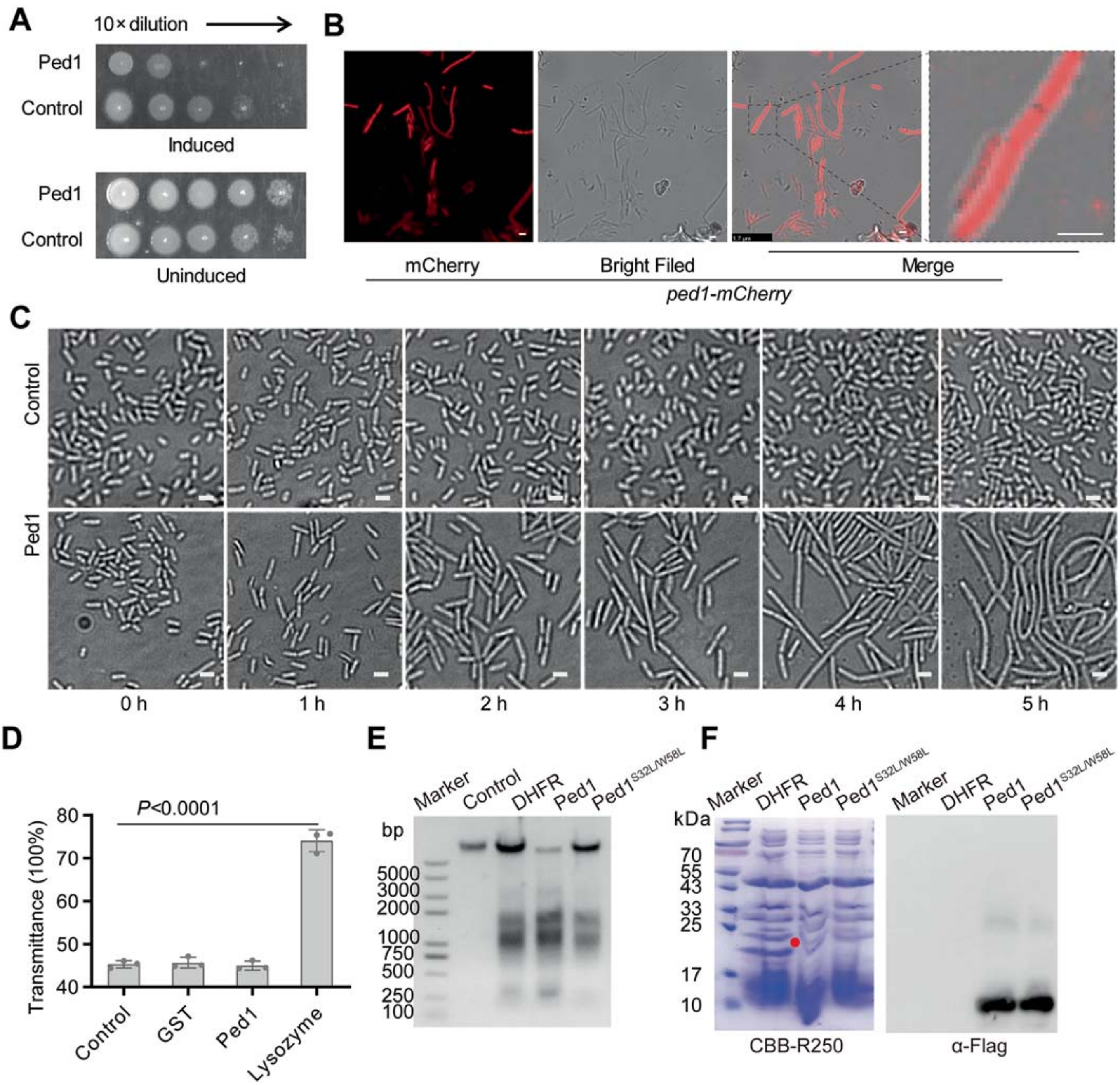

Figure EV1. Localization and phenotypic characterization of Ped1-expressing cells.

(**A**) Growth of *P. aeruginosa* PAO1 on LB agar plates. PAO1 cells harboring an empty vector (Control) or pME6032-ped1 (Ped1) were spotted as tenfold serial dilutions on plates with (Induced) or without (Uninduced) 1 mM IPTG. (**B**) Subcellular localization of Ped1-mCherry. Representative fluorescence, bright field, and merged micrographs of PAO1 cells expressing a Ped1-mCherry fusion protein. Ped1-mCherry is homogeneously distributed within the cytoplasm of filamentous cells. Scale bar, 2.5 μm. (**C**) Time-course microscopy of cell filamentation. Micrographs of PAO1 harboring an empty vector (Control) or expressing Ped1 taken at 1-h intervals (0–5 h) post-induction with IPTG. Scale bar, 2.5 μm. Images at 0 h and 5 h correspond to those presented in Fig. 1B. (**D**) Lysozyme activity assay. The transmittance (100%) of cell wall suspensions was measured after incubation with Control buffer, GST, purified Ped1, or Lysozyme (positive control). Ped1 exhibited no detectable lysozyme activity. (**E**) In vitro DNase activity assay. λ phage genomic DNA was incubated with the indicated in vitro synthesized Ped1 or Ped1$^{S32L/W58L}$ in DNase I buffer containing 1 mM $MgCl_2$ at 37 °C for 60 min. DHFR served as a negative control for the DNase assay. (**F**) CBB-250 staining or western blot analysis of in vitro synthesized proteins. In all, 25-μl reactions containing 250 ng template DNA and 20 units RNase Inhibitor were incubated at 37 °C for 2 h. Two microliters of each reaction were analyzed by SDS–PAGE using a 12% Tris-glycine gel followed by CBB-250 staining or western blot. The red dot indicates the protein of interest. Data are representative of three independent experiments (**A–C**, **E**, **F**). Data are represented as mean ± SD from three independent experiments. Statistical significance was determined by two-way ANOVA (**D**). Source data are available online for this figure.

 © The Author(s)

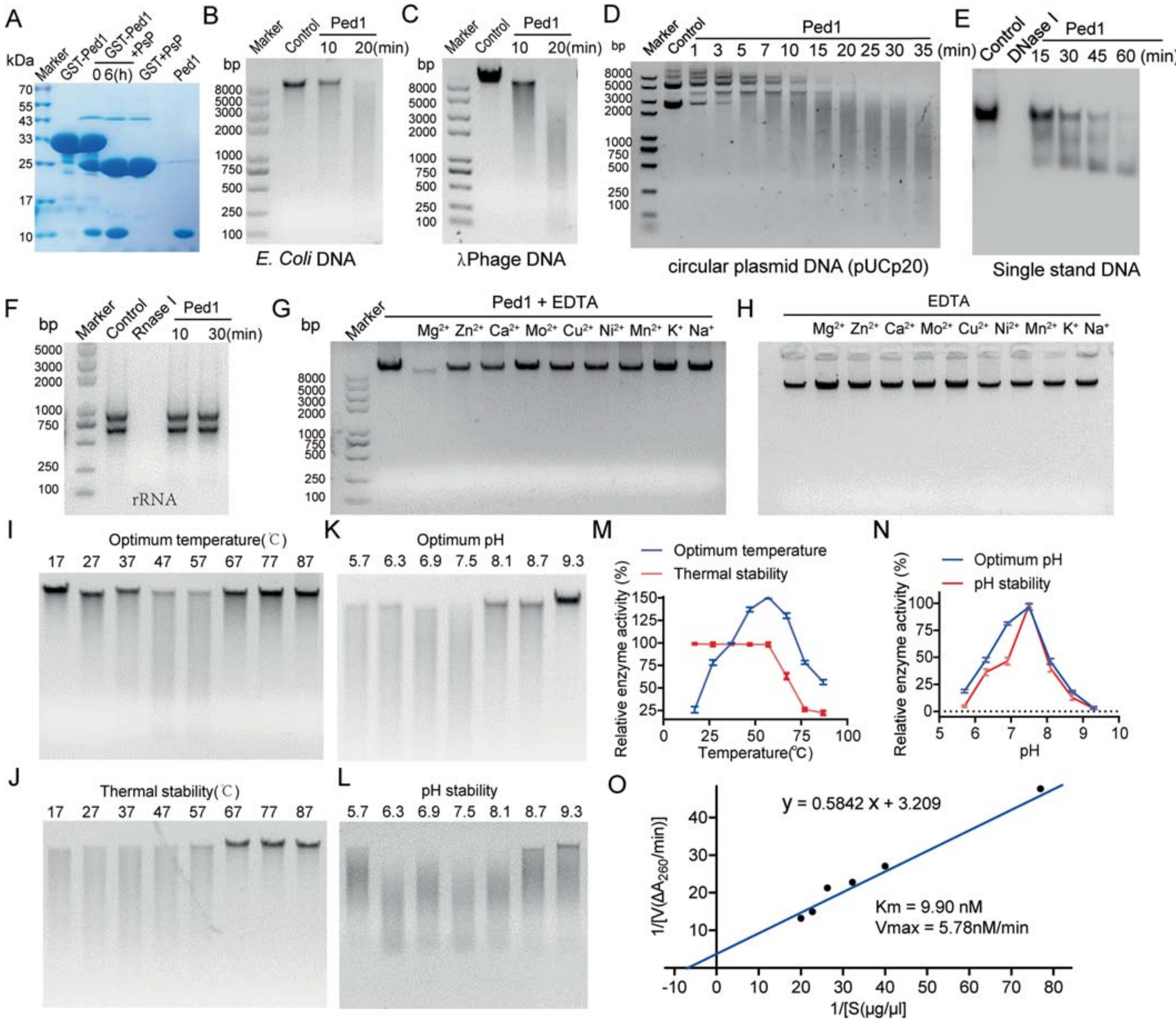

Figure EV2. Biochemical characterization of Ped1 DNase activity.

(**A**) Purification and GST-tag removal of GST-Ped1. (**B**, **C**) *E. coli* (**B**) or λ phage genomic DNA (**C**) was incubated with Ped1 in DNase I buffer containing 1 mM $MgCl_2$ at 37 °C for 20 min. Products were resolved by agarose gel electrophoresis. (**D**, **E**) Activity of Ped1 against circular plasmid DNA (pUCP20) (**D**) and single-stranded DNA (**E**). DNase I served as a positive control. (**F**) RNA hydrolysis assay. Total RNA was incubated with Ped1 and analyzed by agarose gel electrophoresis. (**G**, **H**) Metal ion-dependency of Ped1 nuclease activity. Λ DNA was incubated in DNase I buffer containing a final concentration of 2 mM indicated metal ions for 10 min with (**G**) or without (**H**) Ped1. (**I–L**) Effects of temperature (**I**, **J**) and pH (**K**) on Ped1 nuclease activity against λDNA or its thermal stability. λDNA was incubated with Ped1 for 10 min at the indicated temperatures (**I**). Ped1 was pre-incubated at the indicated temperatures for 10 min, then assayed for activity at 37 °C for 10 min (**K**). λDNA was incubated with Ped1 for 10 min at the indicated pH values (**K**). Products were analyzed using agarose gel electrophoresis. (**L**) Ped1 was pre-incubated in buffers of the indicated pH for 10 min, then assayed at 37 °C for 10 min. (**M**) Optimal temperature and thermal stability of Ped1. (**N**) Optimal pH and pH stability of Ped1. (**O**) Lineweaver-Burk plots for Ped1 activity with λDNA as substrate. Data are representative of three independent experiments (**A–I**). (**M**, **N**) Each point represents the mean ± SD of three independent experiments. Source data are available online for this figure.

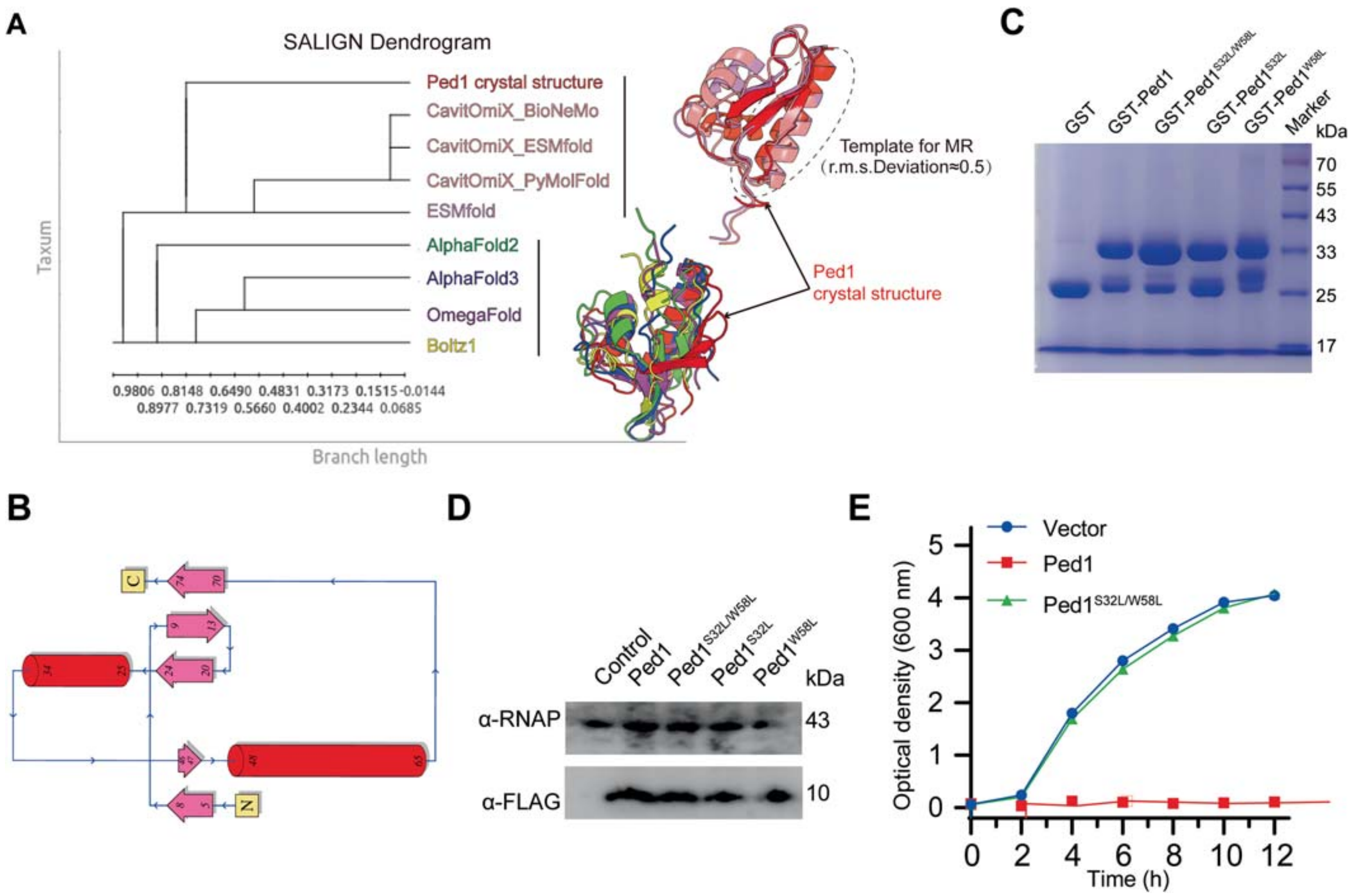

Figure EV3. Structural comparison and mutational analysis of Ped1.

(**A**) Comparison of experimentally determined and predicted Ped1 structures. A dendrogram (left) and superimposed structures (right) show the relationship between the experimental Ped1 crystal structure and various computational models. Despite structural predictions from tools like AlphaFold, critical backbone deviations necessitated the use of PyMol plugin CavitOmiX-generated fragments as search templates for molecular replacement. (**B**) Topological diagram of Ped1. This schematic illustrates the unique α/β-nuclease fold, characterized by a central three-stranded antiparallel β-sheet flanked by two α helices. (**C**) Purification of Ped1 variants. SDS–PAGE analysis confirms the successful purification of GST-tagged wild-type Ped1 and indicated variants (S32L/W58L, S32L, and W58L) used for downstream functional assays. (**D**) Western blot analysis of Ped1 expression. Immunoblotting with α-Flag confirms equivalent expression levels of wild-type Ped1 and its variants in PAO1 cells. α-RNAP was utilized as a loading control. (**E**) Growth dynamics of PAO1 expressing Ped1 variants. A growth curve monitored by OD_{600} demonstrates that while wild-type Ped1 causes severe growth inhibition, the catalytically inactive $Ped1^{S32L/W58L}$ variant lacks physiological toxicity. Data representative of three independent experiments (**C**, **D**). Data are represented as mean ± SD from three independent experiments (**E**). Source data are available online for this figure.

 © The Author(s)

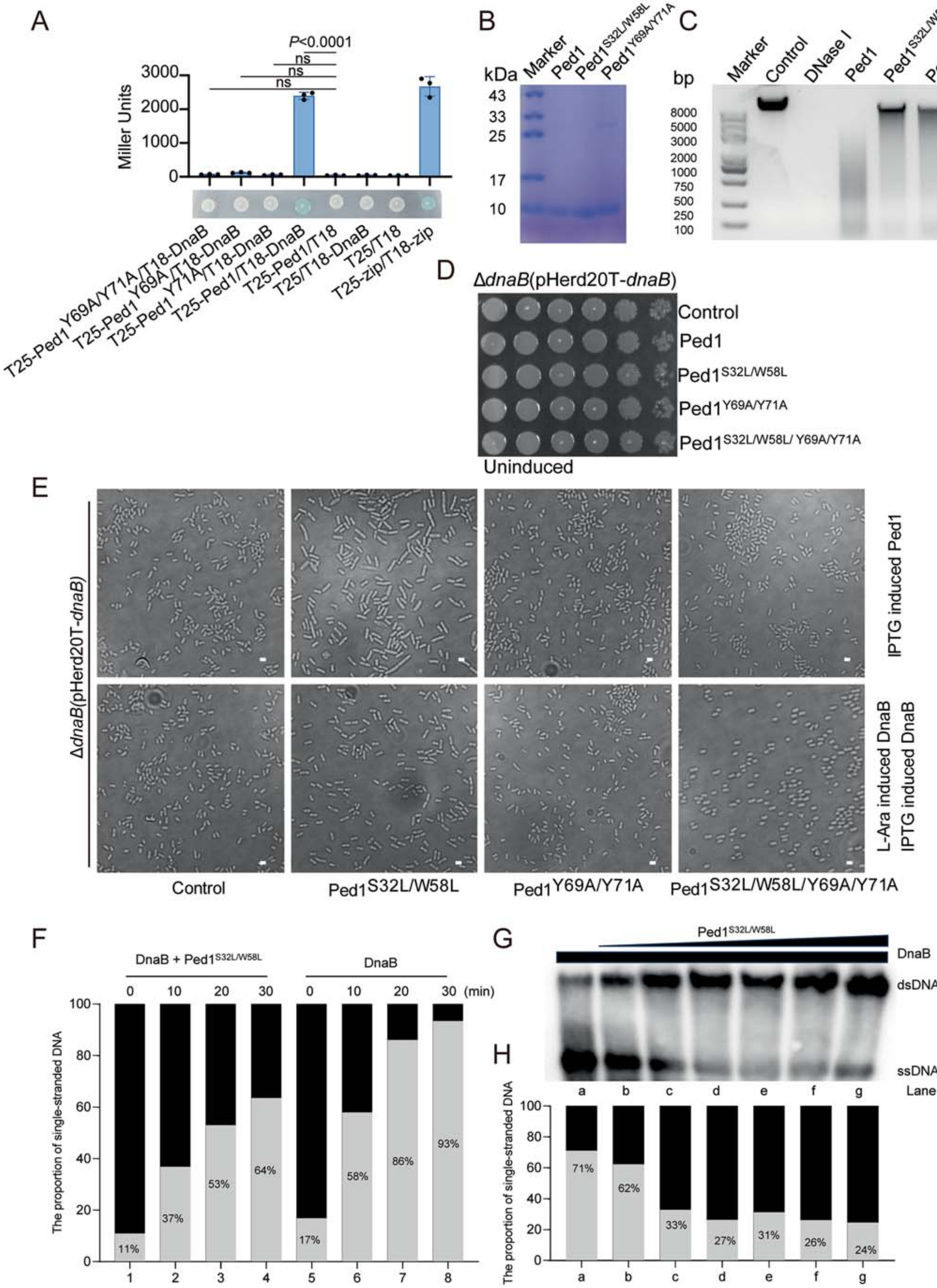
A
P<0.0001
ns
ns
ns
Miller Units
3000
2000
1000
0
T25-Ped1 Y69A/Y71A/T18-DnaB
T25-Ped1 Y69A/T18-DnaB
T25-Ped1 Y71A/T18-DnaB
T25-Ped1/T18-DnaB
T25-Ped1/T18
T25/T18-DnaB
T25/T18
T25-zip/T18-zip
B
kDa
Marker
Ped1
Ped1S32L/W58L
Ped1Y69A/Y71A
43
33
25
17
10
C
bp
Marker
Control
DNase I
Ped1
Ped1S32L/W58L
Ped1Y69A/Y71A
8000
5000
3000
2000
1000
750
500
250
100
D
ΔdnaB(pHerd20T-dnaB)
Control
Ped1
Ped1S32L/W58L
Ped1Y69A/Y71A
Ped1S32L/W58L/ Y69A/Y71A
Uninduced
E
ΔdnaB(pHerd20T-dnaB)
IPTG induced Ped1
L-Ara induced DnaB
IPTG induced DnaB
Control
Ped1S32L/W58L
Ped1Y69A/Y71A
Ped1S32L/W58L/Y69A/Y71A
F
DnaB + Ped1S32L/W58L
DnaB
0
10
20
30
0
10
20
30
(min)
The proportion of single-stranded DNA
100
80
60
40
20
0
11%
37%
53%
64%
17%
58%
86%
93%
1
2
3
4
5
6
7
8
G
Ped1S32L/W58L
DnaB
dsDNA
ssDNA
Lane
a
b
c
d
e
f
g
H
The proportion of single-stranded DNA
100
80
60
40
20
0
71%
62%
33%
27%
31%
26%
24%
a
b
c
d
e
f
g

Figure EV4. Mapping and functional characterization of the Ped1-DnaB binding interface.

(**A**) Identification of residues critical for the Ped1-DnaB interaction. Bacterial two-hybrid (BACTH) analysis shows that the interaction with DnaB is abolished in Ped1 mutants harboring Y69A, Y71A, or Y69A/Y71A substitutions, as indicated by the lack of β-galactosidase activity (Miller Units) and white colony phenotypes on X-gal plates. (**B**) SDS–PAGE analysis of purified wild-type Ped1 and mutants (Ped1$^{S32L/W58L}$ and Ped1$^{Y69A/Y71A}$) used for biochemical assays. (**C**) Assessment of DNase activity in mutants. In vitro nuclease assays demonstrate that nuclease activity is abolished in the DNase-inactive mutant Ped1$^{S32L/W58L}$ and the binding-deficient Ped1$^{Y69A/Y71A}$. (**D**) Growth of *P. aeruginosa* PAO1Δ*dnaB*(pHerd20T-*dnaB*) strains harboring plasmids expressing the indicated Ped1 variants on LB agar without inducer added. (**E**) Representative phase-contrast micrographs of PAO1Δ*dnaB*(pHerd20T-*dnaB*) cells expressing the indicated Ped1 variants, which were acquired at 4 h post-induction. Scale bar, 2.5 μm. (**F**) Quantification of the helicase activity in Fig. 3E. Band intensities of single-stranded and double-stranded DNA were quantified using ImageJ, and the percentage of unwound DNA (ssDNA/total DNA) was calculated for each lane. (**G**) In vitro inhibition of DnaB helicase activity by increasing concentrations of Ped1$^{S32L/W58L}$. Helicase unwinding assays were performed with purified DnaB in the presence of increasing concentrations of the DNase-inactive Ped1 (Ped1$^{S32L/W58L}$). (**H**) Quantification of the helicase assay in Fig. EV4F, performed as described in (**F**). Data are representative of three independent experiments (**B–E**, **G**). Data are represented as mean ± SD from three independent experiments. Statistical significance was determined by ordinary one-way ANOVA with Dunnett's multiple comparisons test (**A**). Source data are available online for this figure.

 © The Author(s)

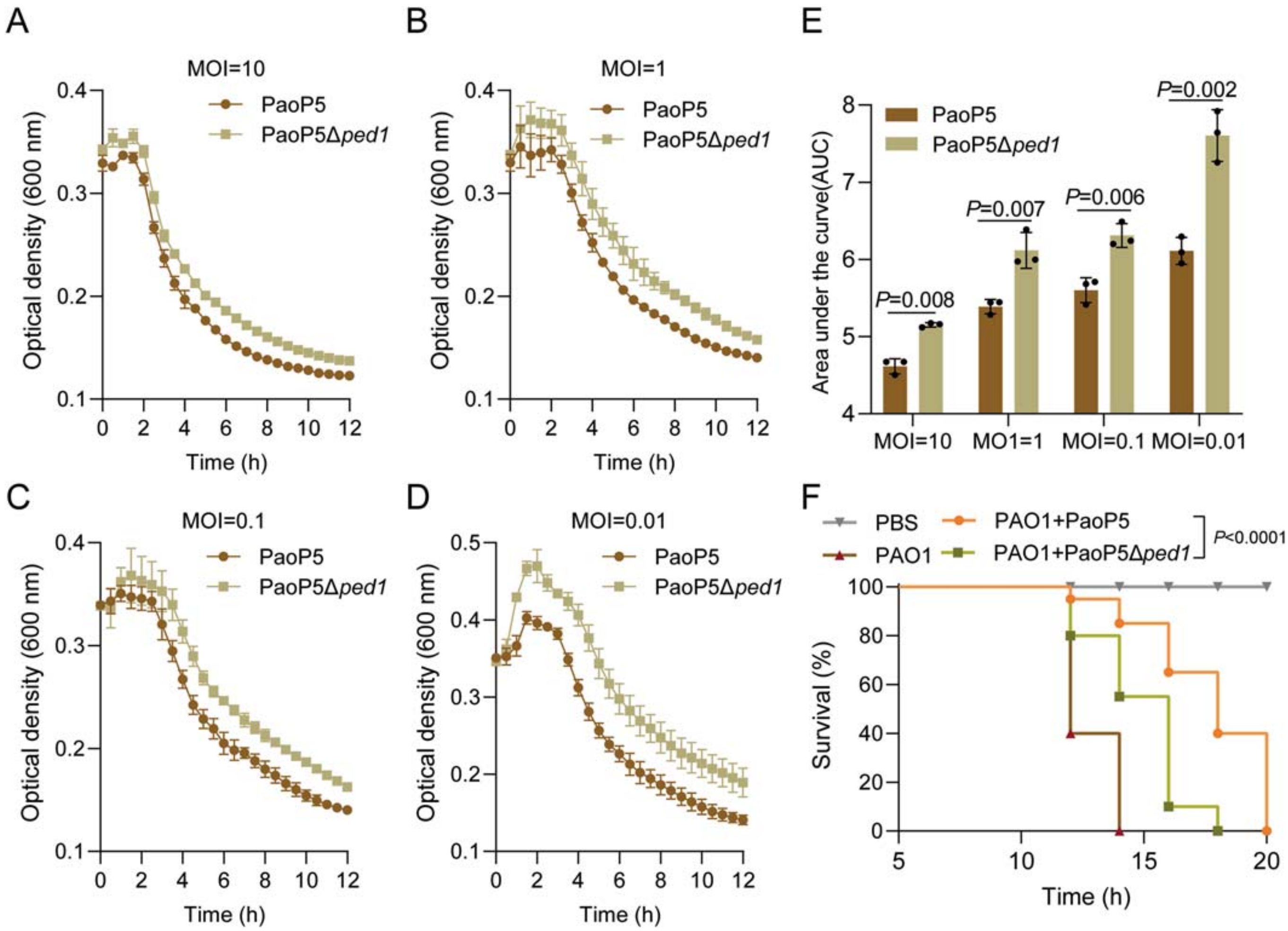

Figure EV5. Deletion of *ped1* significantly compromises phage infectivity.

(A–D) Bacterial lysis kinetics of *P. aeruginosa* PAO1 infected with PaoP5 or PaoP5Δ*ped1* at MOIs of 10, 1, 0.1, and 0.01. Bacterial density (OD_{600}) was monitored every 30 min for 12 h post-infection. **(E)** Quantification of the area under the curve (AUC) for **(A–D)**. **(F)** Survival curves of *Galleria mellonella* larvae infected with PAO1 and treated with PBS, wild-type PaoP5, or PaoP5Δ*ped1*. $n = 20$. Error bars: mean ± SD **(A–E)**. Statistical significance was determined by unpaired *t* test **(E)**. Statistical significance was calculated using the log-rank test **(F)**. Source data are available online for this figure.

© The Author(s)
