# Supplementary material for: A dual-action bacteriophage protein Ped1 degrades DNA and blocks replication in Pseudomonas aeruginosa
Source: EMBO J. 2026 Oct 2;45(21):7541–63. doi: 10.1038/s44318-026-00924-8 (PMC13728149; doi:10.1038/s44318-026-00924-8)
Supplement: Supplementary file 3 [file 44318_2026_924_MOESM2_ESM.pdf]

THE EMBO JOURNAL

A dual-action bacteriophage protein Ped1 degrades DNA and blocks replication in *Pseudomonas aeruginosa*

Tietao Wang, Wenbo Yan, Chen Wang, Longgang Sang, Yingxin Yang, Jingru Zhao, Chao Li, Lijun Liu, Yani Zhang, Shuai Le, and Haihua Liang

Corresponding author(s): Haihua Liang (lianghh@sustech.edu.cn) , Tietao Wang (WangTietao@nwu.edu.cn)

Review Timeline:

Submission Date:	12th Mar 26
Editorial Decision:	14th May 26
Revision Received:	31st Jul 26
Editorial Decision:	1st Sep 26
Revision Received:	12th Sep 26
Accepted:	20th Sep 26

Editor: Ieva Gailite

Transaction Report:

(Note: With the exception of the correction of typographical or spelling errors that could be a source of ambiguity, letters and reports are not edited. Depending on transfer agreements, referee reports obtained elsewhere may or may not be included in this compilation. Referee reports are anonymous unless the Referee chooses to sign their reports.)

1st Editorial Decision **14th May 2026**

Dear Haihua,

Thank you for submitting your manuscript for consideration by the EMBO Journal. I sincerely apologise for the unusually extended assessment process of your manuscript due to delays in reviewer report submission. We have now received comments from two reviewers, which are included below for your information.

As you will see, both reviewers find the study generally of interest and appreciate its contribution to the research field. At the same time, they raise a number of substantive concerns regarding the strength of the evidence for the proposed dual role of Ped1 as a DNase and DnaB inhibitor that would need to be conclusively addressed before they can support publication of the study here.

Based on the interest expressed in the reports, I would like to invite you to submit a revised manuscript in response to the comments by both reviewers. If some of the mechanistic concerns cannot be addressed, EMBO Reports could be a more suitable venue for the revised version. I would be happy to discuss the revision and the feasibility of specific experiments in more detail via email or phone/videoconferencing.

We generally allow three months as standard revision time, which can be extended to six months in the case of major revisions. Should you foresee a problem in meeting this deadline, please let us know in advance to discuss an extension.

As a matter of policy, competing manuscripts published during this period will not negatively impact on our assessment of the conceptual advance presented by your study. However, please contact me as soon as possible upon publication of any related work to discuss the appropriate course of action.

When preparing your letter of response to the referees' comments, please bear in mind that this will form part of the Review Process File and will therefore be available online to the community. For more details on our Transparent Editorial Process, please review our Editorial Policies: https://link.springer.com/partners/embo-press/editorial-policies. Please also see the attached instructions for further guidelines on preparation of the revised manuscript.

Please feel free to contact me if you have any further questions regarding the revision. Thank you for the opportunity to consider your work for publication. I look forward to discussing your revision.

With best wishes,

Ieva

Ieva Gailite, PhD
Senior Scientific Editor
The EMBO Journal
Meyerhofstrasse 1
D-69117 Heidelberg
Tel: +4962218891309
i.gailite@embojournal.org

Instructions for preparing your revised manuscript:

Read our guidance for manuscript revisions and related editorial policies: https://link.springer.com/journal/44318/submission-guidelines#cms-Revised-submissions

Please make sure you upload a letter of response to the referees' comments together with the revised manuscript.

Please also check that the title and abstract of the manuscript are brief, yet explicit, even to non-specialists.

When assembling figures, please refer to our figure preparation guideline in order to ensure proper formatting and readability in print as well as on screen:
https://media.springernature.com/original/springer-cms/rest/v1/content/27825798/data/v1

At EMBO Press we ask authors to provide source data for the main manuscript figures. You will receive a separate email with instructions for providing source data with your revised manuscript, including how to upload and organize the files.

IMPORTANT: When you send the revision we will require

- a point-by-point response to the referees' comments, with a detailed description of the changes made (as a word file).
- a word file of the manuscript text.
- individual production quality figure files (one file per figure)
- a complete author checklist
- Expanded View files (replacing Supplementary Information)
- a Reagents and Tools Table as part of the Methods section

Please remember: Digital image enhancement is acceptable practice, as long as it accurately represents the original data and conforms to community standards. If a figure has been subjected to significant electronic manipulation, this must be noted in the figure legend or in the 'Materials and Methods' section. The editors reserve the right to request original versions of figures and the original images that were used to assemble the figure.

We realize that it is difficult to revise to a specific deadline. In the interest of protecting the conceptual advance provided by the work, we recommend a revision within 3 months (12th Aug 2026). Please discuss the revision progress ahead of this time with the editor if you require more time to complete the revisions. Use the link below to submit your revision:

https://emboj.msubmit.net/cgi-bin/main.plex

Referee #1:

Wang & Yan & Wang et al. from the Zhang and Liang labs studied the role of the proposed nuclease Ped1 from the phage PaoP5 in interplay with DnaB in the host takeover of Pseudomonas. On one side Ped1 is proposed to be a DNase and to degrade the host genomic DNA and on the other side to directly interfere with the DnaB complex to prevent host genome replication, in the end it is toxic to the cell and potentially interacts with DnaB that impedes its DNase activity. The authors characterised the biochemical parameters for DNase activity, solved the structure of this small protein Ped1 and claim in vitro DNase activity and interaction with DnaB. The authors did a lot of work in terms of biochemical characterisation of DNase activity and interaction. In essence, this story represents like an all-in work, where multiple insights were united under the umbrella of a dual-action protein that is per se novel but is it really required to do both?, I guess not if DNA gets degraded, what is then the role of the DnaB interaction. I do have substantial questions related to the individual roles of these actions, and also to the model, as it does not become clear how this all works together in phage infection. In general, if my concerns can be clarified, I do foresee that this work is highly significant for the phage community.

Major comments

The claim that Ped1 has a DNase activity is based on in vitro DNase assays and the TUNEL assay after in vivo expression. Now the outcome of the TUNEL assay can be a secondary effect not directly caused by DNase activity of Ped1. The purification of Ped1 for in vitro assays looks clean, but carryovers and impurities by other DNases cannot be excluded. In this line, in Fig. 1d the activity of Ped1 does appear to be very limited, also after 90 min (very long time for a DNase) compared to DNase I positive control. As DNA gets degraded, but not to the level of single nucleotides, the fragment sizes between 500-1000 bp suggest a specific cleavage at defined sites that cannot be explained by the Ped1 structure given in Fig. 2, as this is too small to facilitate sequence recognition, hence it may be an impurity that does the cleavage in the background. In addition, the structure does not show a clear positively charged pocket to bind DNA, the here proposed catalytically active residues serine and tryptophane are so far not described to be able to coordinate a magnesium for cleavage as suggested by Fig. 1e. All structure predictions about a nuclease are nice and shiny, but ultimately this is all based on predictions and docking. A search for structural homology does not yield any links to DNases, that are actually quite well characterised and conserved, and the authors do not dwell on this but rather claim a novel DNase fold, that would be novel if true. Functional assays of the mutants in vivo show effects, but still it remains to validate direct action on DNA breaks, and in vitro assays, again the purification may be different with a mutated protein, and the cleavage of the GST was not complete (Fig. S4c). In essence, the authors must convincingly show that Ped1 is indeed a DNase without any risk of contaminations. I suggest to produce Ped1 by in vitro translation in highly purified systems like PUREexpress that lacks any DNases and to confirm that the products (no purification required) are DNase active, please also include the catalytically dead variants.

Ped1 is claimed here to bind the hexameric DnaB complex (6x51.4 kDa = 308.4 kDa) but this is not evident from the SEC-MALS, as the mass is estimated about 305 kDa, this is the mass without Ped1, the kd is pretty low, and the inhibition of Ped1 in Fig. 4a could be again related to interactions with DNases in the background, or in Fig. 4d,e related to indirect effects. The elution profiles in Fig. S5b require a Coomassie gel, and the absorption profiles do not add up that suggests aggregation or non-equivalent sample materials in injections. Please comment on these points, but to convincingly show the interaction between DnaB and Ped1, the authors should consider single-particle cryo-EM.

How can effects on genome replication be discriminated from interference with DnaB? It remains very important to check the

genome copy number per cell after expression of Ped1 to solidify the claims about replication, e.g. by Southern blotting. The filamentous growth form upon expression of Ped1 was here linked to a blocking in cell replication, but this phenotype is very commonly induced upon stress. It does also not become clear to me, why ped1 expression should increase the expression of dnaB and how this supports the model, aside that this is an indirect effect.

Related to the model (where a phage is shown), Ped1 shows a strong effect upon expression alone, but after deletion in PaoP5 there is no effect. The authors should follow up on this and identify conditions where the delta phage is affected in progeny or replication time, and cross-validate previous physiological effects from overexpression of ped1.

Minor comments
Line 168, Show the AlphaFold 2/3 models
Please add a sequence alignment for all the Ped1 homologs in Fig. 6a, indicate also the proposed catalytic residues, these must be conserved.

Other comments
The references given in the text are not acceptable, the first authors should be indicated clearly.
Line 94. The term „lytic virus" is wrong, it is a virulent virus.
Dnase I should be DNase I.
Fig. 1b, at which IPTG concentration was this recorded, how does it correlate to Fig. 1a, keep here in mind related to the title of the results paragraph, that the cells are growing, hence Ped1 has at this stage no effect on the cell growth but division.
"Bioinformatic analysis showed that Ped1 shares no significant sequence similarity with any protein studied before." This strong statement needs some data or being rephrased.
Please give the column used in SEC-MALS and comment about the different elution compared to Fig. S5b.

Referee #2:

Wang et al. report the discovery and characterisation of a phage-encoded protein, Ped1, which acts as a nuclease and blocks DNA replication during infection of Pseudomonas aeruginosa. Ped1 was previously identified as an inhibitor of Pseudomonas, and here is characterised structurally and biochemically.

The introduction provides an informative and balanced account of the mechanisms used by phages to subvert host physiology in Pseudomonas.

Overexpression of fluorescent Ped1 causes a filamentation phenotype (Fig1), which correlates with the DNase activity of the protein in vivo and in vitro. The X-ray structure reveals a novel fold and conserved active site residues are identified (Fig 2). Ped1 is shown to interact specifically with the hexameric replicative helicase DnaB (Fig 3). This interaction inhibits DnaB helicase activity and also inhibits Ped1 nuclease activity. It should be said that the evidence for DnaB helicase inhibition is rather weak and could be strengthened if this claim is considered significant.

Overall, this is an interesting body of work that brings many different techniques to bear on the function of the Ped1 nuclease during phage infection. The data for Ped1 nuclease activity are solid, as is the interaction with DnaB (although stoichiometry is unclear as written). The evidence that DnaB is inhibited by Ped1 binding is rather weak and needs to be strengthened if this remains a central claim of the paper.

Specific points:
1. Line 152. Km should be reported in units of concentration, eg nM and Vmax in reciprocal time.
2. For the kinetic analysis, the methods do not describe the conditions of the nuclease assay used - in particular the substrate and enzyme concentrations. Have steady-state, multiple turnover conditions been applied?
3. In figure S2, a Michaelis-Menten plot is mentioned in the figure legend, but not shown.
4. In figure S2m, n, means of duplicate measurements are shown {plus minus} SD. Statistical analysis cannot be carried out with less than triplicate measurements.
5. In figure S2o, error bars are suggested but seem to be absent.
6. Please submit the structure to the DALI server, which is the gold standard for structural comparisons, and report the most significant matches. Does the core topology match any known nuclease superfamily? Is the enzyme a monome? - what does PISA analysis of the structure indicate?
7. With a single active site, Ped1 would be expected to nick dsDNA in the first instance. This could be tested by incubating the protein with a supercoiled plasmid and looking for relaxed products at early time points.
8. In figure S4, parts d and e are mis-labelled.
9. Figure S5a - clarify what label # signifies. How often was this experiment carried out?
10. Figure S5e - the gels showing purity of proteins are heavily cropped. Please provide uncropped gels to allow the reader to

assess the purity objectively.

11. Line 220 - what does "recruits" mean in this context? Do you mean "binds"?
12. Line 220 - is a Kd of 3.2 µM high affinity for a protein:protein interaction? Can the authors provide some examples of other high affinity protein interactions with similar Kds in the literature?
13. Figure 3 and accompanying text. DnaB is a hexamer and Ped1 is suggested to be a monomer. Do the ITC data support a 1:1 (ie 6:6) complex of the two proteins? How does this fit with figure 3f which shows a mass for the complex of 300 kDa -roughly equal to 6 DnaB subunits but not a 6:6 complex?
14. For the ITC experiments, more details of the experimental design are required. What was the fitting model used? Were other models also tested? How often was this experiment repeated - it looks like a single time from the text.
15. Figure 3e - DnaB unwinding assay. It is possible that the variant Ped1 used here can still bind to ssDNA and dsDNA. This could result in inhibition without requiring an interaction with DnaB. Can the authors please test this possibility?
16. The methods section suggests that the wt Ped1 protein was also tested with DnaB in the unwinding assay, but this data is not shown. Can the authors clarify please?
17. For the unwinding assay, why were purified Ped1-DnaB complexes assayed rather than adding increasing concentrations of Ped1 to DnaB in the assay? These experiments would provide important further confirmation of this inhibition.
18. Lines 350-355. These comments are very speculative and founded on rather sparse evidence, so should be toned down or removed.
19. the model in figure 6 requires that Ped1 and DnaB are expressed at similar levels in infected cells. Is there any evidence for this? If not, this issue needs to be discussed and caveated.
20. Figure 6. What does "evolutionary decoy" mean? Where is evolution coming into this mechanism, other than the fact that all the players co-evolve, which is true for everything in this context. Likewise, the term "strategic highjacker" seems unnecessary and anthropomorphic.
21. Many phage express DNA nucleases that degrade host DNA to provide nucleotides for phage DNA synthesis. This should be discussed in a paragraph in the discussion to place the work in context.

Dear editor and reviewers:

We are deeply grateful to the editor and the reviewers for their constructive comments and careful evaluation of our manuscript "**A dual-action bacteriophage protein Ped1 degrades DNA and blocks replication to subvert *Pseudomonas*" (ID: EMBOJ-2026-124104).** All concerns have been carefully addressed, and the manuscript has been revised according to the reviewers' suggestions. Our point-by-point responses follow below.

Response to Reviewers:

Referee #1:

Wang & Yan & Wang et al. from the Zhang and Liang labs studied the role of the proposed nuclease Ped1 from the phage PaoP5 in interplay with DnaB in the host takeover of Pseudomonas. On one side Ped1 is proposed to be a DNase and to degrade the host genomic DNA and on the other side to directly interfere with the DnaB complex to prevent host genome replication, in the end it is toxic to the cell and potentially interacts with DnaB that impedes its DNase activity. The authors characterised the biochemical parameters for DNase activity, solved the structure of this small protein Ped1 and claim in vitro DNase activity and interaction with DnaB. The authors did a lot of work in terms of biochemical characterisation of DNase activity and interaction.

In essence, this story represents like an all-in work, where multiple insights were united under the umbrella of a dual-action protein that is per se novel but is it really required to do both?, I guess not if DNA gets degraded, what is then the role of the DnaB interaction. I do have substantial questions related to the individual roles of these actions, and also to the model, as it does not become clear how this all works together in phage infection. In general, if my concerns can be clarified, I do foresee that this work is highly significant for the phage community.

Response: We sincerely appreciate your positive evaluation for our manuscript. We have done several experiments to address your concerns.

Major comments

The claim that Ped1 has a DNase activity is based on in vitro DNase assays and the TUNEL assay after in vivo expression. Now the outcome of the TUNEL assay can be a secondary effect not directly caused by DNase activity of Ped1. The purification of Ped1 for in vitro assays looks clean, but carryovers and impurities by other DNases cannot be excluded. In this line, in Fig. 1d the activity of Ped1 does appear to be very limited, also after 90 min (very long time for a DNase) compared to DNase I positive control.

Response: We thank the reviewer for this important concern. We agree that TUNEL positivity alone cannot conclusively demonstrate direct nuclease activity, as DNA

fragmentation may arise indirectly from cellular stress responses. To address this concern, DNase I, a recognized positive control for DNase assay, was used to verify the Ped1-mediate DNA damage in TUNEL assay. Results show that both Ped1 and DNase I-treated bacteria also displayed TUNEL-positive staining, which confirmed that the TUNEL assay faithfully detects DNA fragmentation in our experimental system (**Fig SS1 and Appendix Figure S1A**). These data further support that the TUNEL signal observed in Ped1-expressing cells indicates direct DNA damage caused by Ped1.

In line with the above observation, several independent observations support the intrinsic DNase activity of Ped1. **(1)** Purified Ped1 efficiently degraded multiple DNA substrates *in vitro*, including genomic DNA, λ DNA, plasmid DNA, and single-stranded DNA (**Fig. EV2B-E**). **(2)** When purified using the same BL21(DE3) host strain, purification procedure, and buffer system, the GST control (related to GST-Ped1) and the catalytic mutant Ped1$^{S32L/W58L}$ (related to wild-type Ped1) exhibited no detectable nuclease activity (**Fig. 2D**). **(3)** Wild-type and mutant Ped1 proteins were synthesized using the PURExpress® *In Vitro* protein synthesis system. The in vitro-synthesized wild-type Ped1 protein retained DNase activity, whereas the catalytic mutant (Ped1$^{S32L/W58L}$) remained inactive. (**Fig. EV1E**). These results strongly support that DNA degradation is an intrinsic property of Ped1 rather than the consequence of contaminating nucleases.

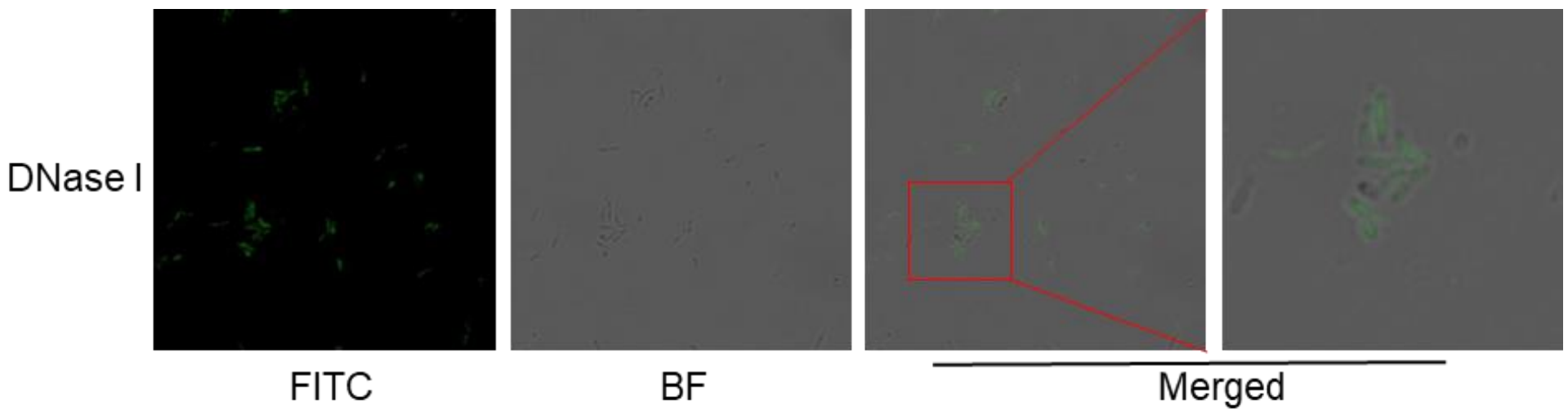

<u>Fig SS1</u>. DNase I treatment induces TUNEL-positive staining in *P. aeruginosa* PAO1. PAO1 cells were treated with DNase I (positive control) and then subjected to TUNEL assay. Green fluorescence (FITC) indicates the presence of DNA strand breaks in DNase I-treated cells.

As DNA gets degraded, but not to the level of single nucleotides, the fragment sizes between 500-1000 bp suggest a specific cleavage at defined sites that cannot be explained by the Ped1 structure given in Fig. 2, as this is too small to facilitate sequence recognition, hence it may be an impurity that does the cleavage in the background.

Response: We appreciate your careful and insightful observation. Unlike DNase I, Ped1 does not completely degrade DNA into mononucleotides under the experimental conditions tested. Therefore, we propose that Ped1 possesses basal nuclease activity that mediates progressive degradation rather than complete digestion. Meanwhile, several reports have also identified serial new DNases which, in *in vitro* DNase assays with purified proteins, do

not achieve the complete digestion into mononucleotides as DNase I does (Song *et al.*, 2021, *Nat Commun* 12: 423; Gruenig *et al.*, 2011, *J Biol Chem* 286: 8240-8251; Smits *et al.*, 2009, *EMBO Rep*, 10: 592-8 and Tuck *et al.*, 2024, *Cell* 187: 6914-6928 e20).

In addition, the structure does not show a clear positively charged pocket to bind DNA, the here proposed catalytically active residues serine and tryptophane are so far not described to be able to coordinate a magnesium for cleavage as suggested by Fig. 1e.

Response: We thank Reviewer for this incisive comment on the electrostatics of the Ped1 active site and on the residue assignments in **Fig. 1E**. We address the two issues separately, (i) the absence of an obviously basic DNA-binding surface, and (ii) the proposed roles of Ser32 and Trp58, and we now state explicitly which conclusions are model-derived hypotheses versus experimentally supported.

(1) A nuclease active site does not present a basic, electropositive DNA-binding surface. The premise that DNA nucleases recruit substrate through a conserved basic groove is not general, and structurally characterized precedents show the opposite. For example, In the C-terminal nuclease domain of the Bacillus subtilis phage SPP1 large terminase (G2P), Smits *et al*. found that "The surface of G2P is mostly negatively charged … the active site groove is largely acidic, although sporadic basic residues are located on the periphery of the groove", and concluded that these "positively charged residues are not conserved in closely related phages … suggesting that DNA binds to the active site mostly through shape-complementarity rather than charge–charge interactions"(Smits *et al*, 2009, *EMBO Rep* 10:14). The same principle holds for a canonical cellular nuclease: for human RNase H1, Nowotny et al. reported that "Although RNase H1 is overall positively charged, the substrate interface is neutral to acidic in character, which likely contributes to the catalytic specificity" (Yang *et al*, 2006, *Mol Cell* 22:5-13)

(2) The catalytic metal, not a basic surface, provides the local electrostatics — and the metal site is not fully formed in the apo enzyme. Ped1 is a strictly Mg^{2+}-dependent nuclease. In the two-metal-ion mechanism shared by the RNase H-like superfamily, the divalent cations neutralize the negative charge of the scissile phosphate and bridge enzyme and substrate; Yang *et al*. describe how "the two Mg^{2+} ions are jointly coordinated by the nucleic-acid substrate and catalytic residues"(Yang *et al*, 2006, *Mol Cell* 22:5-13). Critically, such metal sites are often only weakly occupied until substrate is present — as Nowotny et al. note, the "metal-binding sites do not attain full affinity until a nucleic acid is bound" (a point echoed by Smits et al. for G2P) (Smits *et al*, 2009, *EMBO Rep* 10:14). This is perhaps why an apo crystal structure may not display an obvious electropositive, metal-loaded active site.

(3) The apo, monomeric crystal likely represents a latent/regulated state. Virus-encoded

nucleases frequently keep their active sites auto-restrained to avoid premature genome cleavage. In G2P, Smits et al. showed that "the nuclease activity is regulated by movement of the β-hairpin, altering active site access and the orientation of catalytically essential residues"(Smits *et al*, 2009, *EMBO Rep* 10:14). It is possible that the resting Ped1 structure captures a latent state, with the productive DNA-binding/catalytic configuration induced upon substrate binding and/or upon interaction with host DnaB.

Taken together, the final productive DNA-binding/catalytic configuration induced upon substrate binding is difficult to be determined based on current experimental evidence. What we presented in **Fig. 2C** is actually a knowledge-based model, that is, the protein-DNA complex was actually modelled after we confirmed the key residues of Ped1 and Mg^{2+}-dependent properties, which is consistent with the experimental evidence.

As we mentioned above, the initial identification of effective mutation sites actually originated from random mutations. And the docking model presented is a knowledge-based model that is consistent with the experimental evidence. In fact, whether conformational changes induced by DNA binding, and water molecules exist or not, number of metal ions and water molecules located in the pocket are all not sure. The final productive DNA-binding/catalytic configuration induced upon substrate binding is difficult to be determined. But alanine substitutions at these positions (S32L, W58L, and S32L/W58L) abolish nuclease activity in vitro (**Fig. 2D**) and eliminate Ped1 toxicity in vivo, independently confirming that both residues are essential for the active site. Moreover, Ser32 as a direct metal ligand is well precedented. A serine hydroxyl can directly chelate a catalytic Mg^{2+}: in yeast enolase, Wedekind et al. titled their structure "Chelation of serine 39 to Mg^{2+} latches a gate at the active site of enolase" (PDB 1EBG) (Wedekind *et al*, 1994, *Biochemistry* 33: 9333-42). As for Trp58, here we revised the sentence and we do not claim that the Trp58 side chain directly coordinates the catalytic Mg^{2+} (Line 174).

All structure predictions about a nuclease are nice and shiny, but ultimately this is all based on predictions and docking. A search for structural homology does not yield any links to DNases, that are actually quite well characterised and conserved, and the authors do not dwell on this but rather claim a novel DNase fold, that would be novel if true.

Response: Thanks for your insightful comment on contents about the "novel fold" and homology search. We submitted the Ped1 crystal structure (query chain s001A) to the Dali server, which returned 86 hits ranked by Z-score (**Data Set EV7**). The top hit, 9jp6-A (annotated "nuclease encoded by gene PaoP5_157"), corresponds to Ped1 itself (Z = 17.2, RMSD = 0.1 Å, 100% sequence identity) and therefore represents the query structure rather than a structural homolog. Excluding this self-match, the highest Z-score was only 3.9 (6uek-A, urocanate hydratase; and 6ywc-C, an antibody heavy chain); no hit reached

Z ≥ 4, only four hits fell in the range 3.5 ≤ Z < 4, and the remaining 81 hits had Z-scores between 2 and 3.5. All of these matches are at the noise level: they share only 4–15% sequence identity, superpose with high RMSD (2.0–3.9 Å) over short alignments (lali = 43–58 residues), and correspond to functionally unrelated proteins spanning disparate families (*e.g.*, urocanate hydratase, an antibody fragment, ClpS, a PI3K regulatory subunit, CRISPR/Cas proteins, and various polymerases and transporters). Such a distribution—uniformly low Z-scores, negligible sequence identity, and no coherent structural family—is the pattern expected in the absence of any genuine structural homolog.

Functional assays of the mutants in vivo show effects, but still it remains to validate direct action on DNA breaks, and in vitro assays, again the purification may be different with a mutated protein, and the cleavage of the GST was not complete (Fig. S4c). In essence, the authors must convincingly show that Ped1 is indeed a DNase without any risk of contaminations. I suggest to produce Ped1 by in vitro translation in highly purified systems like PUREexpress that lacks any DNases and to confirm that the products (no purification required) are DNase active, please also include the catalytically dead variants.

Response: Thanks for your comments and constructive suggestions. We synthesized wild-type and mutant Ped1 using the PURExpress® *In Vitro* Protein Synthesis system. The *in vitro* synthesized wild-type Ped1 protein retained DNase activity whereas the catalytic mutant (Ped1$^{S32L/W58L}$) remained inactive (**Fig EV1E-F**), confirming that the nuclease activity is intrinsic to Ped1 and not derived from contaminating bacterial nucleases.

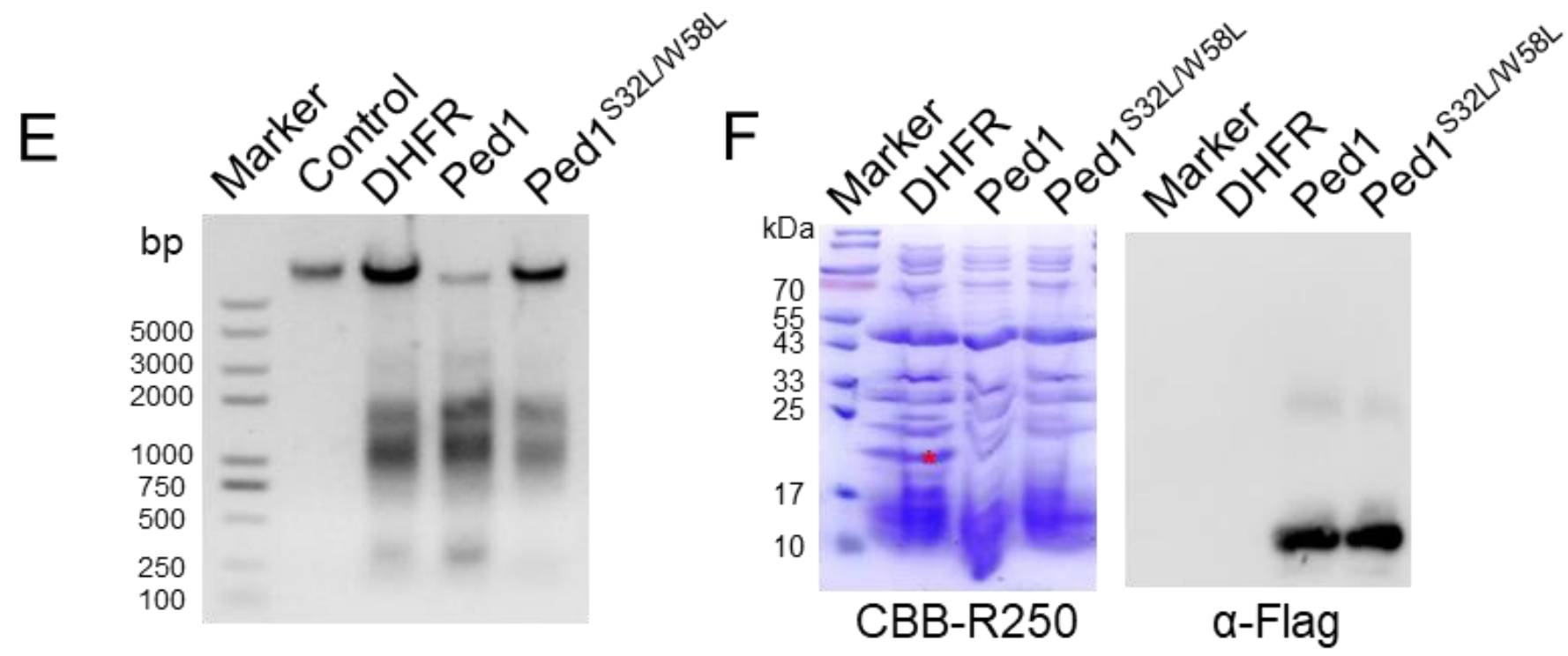

Fig. EV1E-F (in manuscript text). *In vitro* synthesis of Ped1 and examination of its DNase activity. (**E**) In vitro DNase activity assay. λ phage genomic DNA was incubated with the indicated in vitro synthesized Ped1 or Ped1$^{S32L/W58L}$ in DNase I buffer containing 1 mM $MgCl_2$ at 37°C for 60 min. DHFR served as a negative control for the DNase assay. (**F**) CBB-250 staining or western blot analysis of in vitro synthesized proteins. 25-μl reactions containing 250 ng template DNA and 20 units RNase Inhibitor were incubated at 37°C for 2 hours. Two μl of each reaction was analyzed by SDS-PAGE using a 12% Tris-glycine gel followed by CBB-250 staining or western blot. The red dot indicates the protein of interest.

Ped1 is claimed here to bind the hexameric DnaB complex (6x51.4 kDa = 308.4 kDa) but this is not evident from the SEC-MALS, as the mass is estimated about 305 kDa, this is the mass without Ped1, the kd is pretty low, and the inhibition of Ped1 in Fig. 4a could be again related to interactions with DNases in the background, or in Fig. 4d,e related to indirect effects. The elution profiles in Fig. S5b require a Coomassie gel, and the absorption profiles do not add up that suggests aggregation or non-equivalent sample materials in injections. Please comment on these points, but to convincingly show the interaction between DnaB and Ped1, the authors should consider single-particle cryo-EM.

Response: We thank the reviewer for this critical question.

(1) In order to confirm the DnaB:Ped1 stoichiometry, except SEC-MALS, we also tried quantitative mass imaging of single biological macromolecules by mass photometry (Young *et al*, 2018 *Science* 360:423-427). The mass photometry analysis of the purified DnaB-Ped1 complex resolved three populations. The dominant species (329 ± 58 kDa; 70% of counts) corresponds closely to the calculated mass of a DnaB hexamer bound to a single Ped1 molecule (6 × 52.7 + 9.5 = 325.7 kDa). This is not a 6:6 complex, which would have a calculated mass of approximately 6 × (52.7 + 9.5) ≈ 373 kDa. The observed 329 kDa species is therefore basically consistent with a 1 Ped1: 1 DnaB hexamer stoichiometry. However, the additional minor populations observed—117 ± 39 kDa (19%) and 614 ± 99 kDa (9%)—likely represent sub-hexameric DnaB species or dimeric complexes, reflecting the inherent compositional heterogeneity of the protein preparation rather than multiple physiologically relevant binding modes (**Appendix Fig. S2F**). The compositional heterogeneity, together with peak widths (σ) that are large relative to the mass of a single Ped1 molecule (~9.5 kDa), precludes assigning a single precise DnaB:Ped1 stoichiometry. We already substitute SEC-MALS result with mass photometry result and revised relevant contents (Lines 212-220, 367-375).

(2) Regarding the ITC data: As the reviewer correctly notes, the n value derived from our ITC titration was less than 1(which may lie in the sample heterogeneity or the relative activity of the sample is less than 1.), making it difficult to assign a precise DnaB:Ped1 stoichiometry from the ITC data alone. However, ITC remains a robust method for determining binding affinity (Kd), and we obtained a consistent Kd of 2.7 ± 0.35 μM , confirming a specific interaction between Ped1 and DnaB.

(3) We completely share the reviewer's vision that a single-particle cryo-EM structure would represent the ultimate structural depiction of this complex. In fact, inspired by a similar rationale, our team had already actively attempted single-particle cryo-EM screening for the DnaB–Ped1 complex prior to submission. Unfortunately, despite rigorous optimization of grids and freezing conditions, we were unable to resolve a high-resolution reconstruction of the complex.

How can effects on genome replication be discriminated from interference with DnaB? It remains very important to check the genome copy number per cell after expression of Ped1 to solidify the claims about replication, e.g. by Southern blotting.

Response: Thanks for this important suggestion. To genetically separate the nuclease and replication-inhibitory functions of Ped1, we compared wild-type Ped1 with separation-of-function mutants. Specifically, Ped1$^{S32L/W58L}$ lost detectable nuclease activity but retained DnaB interaction and growth inhibitory activity in the conditional *dnaB* knockout strain (**Fig. 2D and Appendix Figure S2D**). In contrast, Ped1$^{Y69A/Y71A}$ lost both DnaB binding and detectable nuclease activity and failed to inhibit growth in the conditional *dnaB* knockout strain (**Fig. EV4A and EV4C**). Because radioactive labeling was unavailable, we monitored genomic DNA replication by qPCR instead of Southern blotting, following a previously reported protocol (Korem *et al.,* 2015, *Science* 349:1101-1106; Pang *et al.*, 2017, *PLoS Genet* 13: e1006908). The results showed that expression of Ped1 led to a significant decrease in the CT(*ori/ter*) ratio, indicating that inhibition of DnaB activity directly causes replication abnormalities. In contrast, the DnaB binding deficient mutant Ped1$^{Y69A/Y71A}$ failed to decrease the CT(*ori/ter*) ratio, which returned to normal levels (**Fig. 3F**). Together, these findings indicate that inhibition of DNA replication can be genetically separated from nuclease activity and is associated with DnaB targeting.

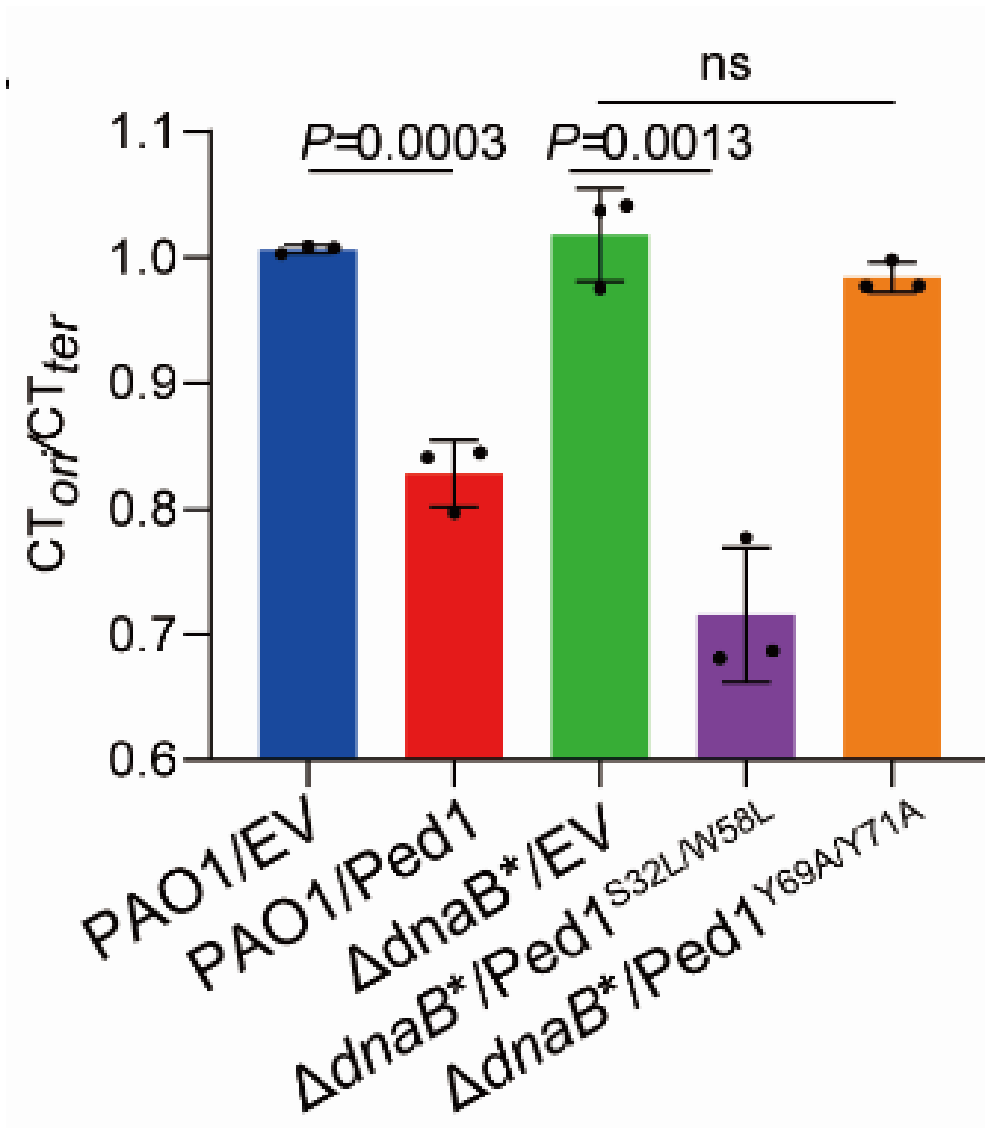

Fig 3F. Replication activity assessed by *ter/ori* ratio. The CT(*ori/ter*) ratio was determined by qPCR in the indicated *P. aeruginosa* PAO1 or Δ*dnaB*(pHerd20T-*dnaB*) (Δ*dnaB**) strains.

The filamentous growth form upon expression of Ped1 was here linked to a blocking in cell replication, but this phenotype is very commonly induced upon stress. It does also not become clear to me, why ped1 expression should increase the expression of dnaB and how this supports the model, aside that this is an indirect effect.

Response: We agree with your assessment. Although filamentation is well-established consequence of DNA damage, which is triggered by SOS responses and other cellular stresses, it is not diagnostic of replication inhibition alone. Consistent with this interpretation, RNA-seq data and qRT-PCR analyses revealed that Ped1 expression altered multiple SOS-associated genes, such as *recA* and *lexA*. The increased expression of *dnaB* is part of the cellular response to replication stress and DNA damage, but not a direct regulatory effect of Ped1. Meanwhile, the catalytically inactive mutant Ped1$^{S32L/W58L}$ does not alter the *dnaB* expression **(Fig. SS2**).

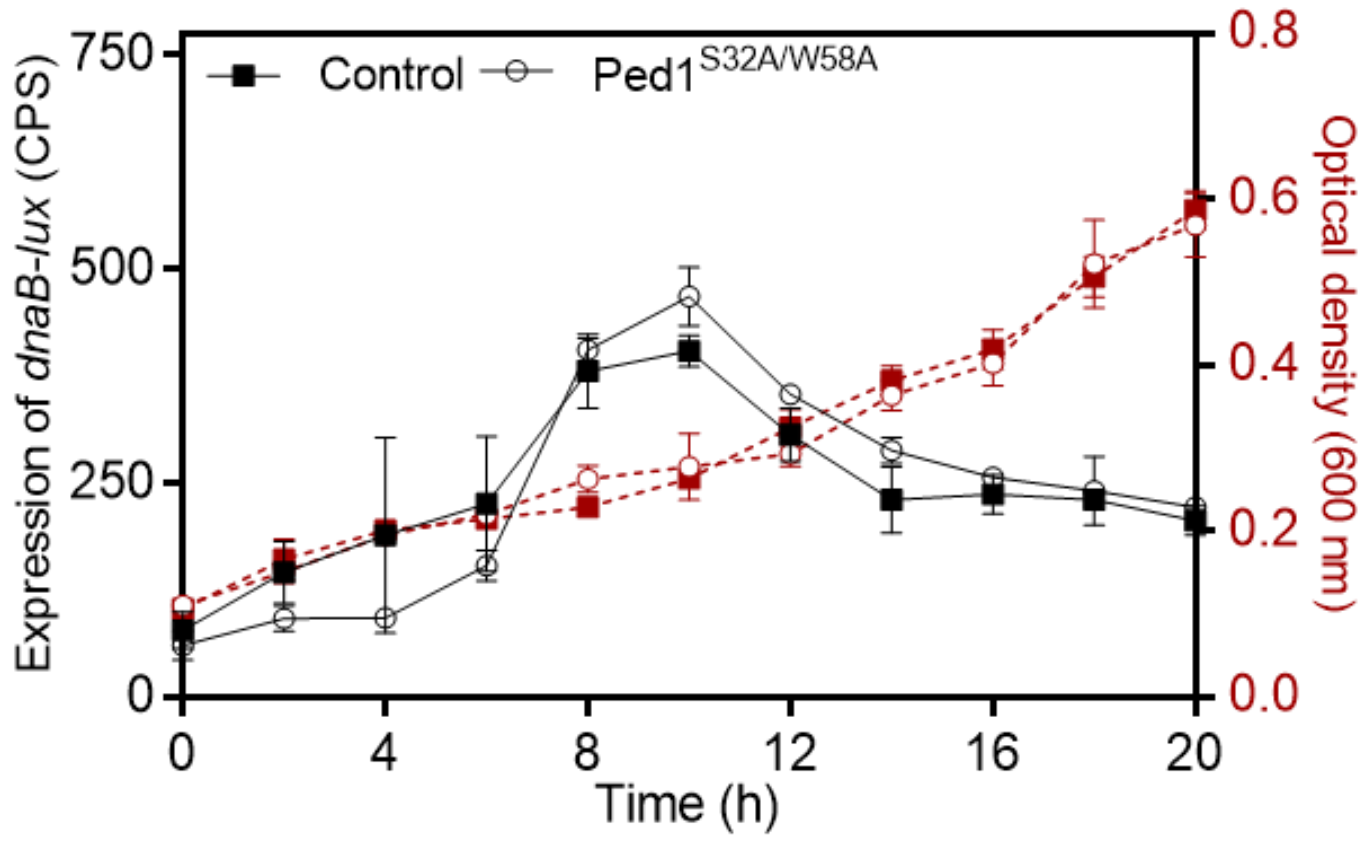

Fig SS2. Validation of Ped1-induced *dnaB* expression via a luciferase reporter assay. The activity of the *dnaB* promoter (P*dnaB-lux*) was monitored over 20 hours in cells expressing Ped1 and Ped1$^{S32L/W58L}$ compared to a vector control.

Related to the model (where a phage is shown), Ped1 shows a strong effect upon expression alone, but after deletion in PaoP5 there is no effect. The authors should follow up on this and identify conditions where the delta phage is affected in progeny or replication time, and cross-validate previous physiological effects from overexpression of ped1.

Response: We appreciate your valuable comments. We agree that elucidating the physiological function of Ped1 during phage infection constitutes a key component of this study. To further address this question, we constructed a gene-deletion mutant of the phage PaoP5Δ*ped1* and conducted a series of experiments to characterize its associated phenotypes. The bacterial lysis data demonstrated that the deletion of *ped1* markedly impairs phage lytic activity against *P. aeruginosa* PAO1 (**Fig. EV5A-E**). Furthermore, using the *Galleria mellonella* infection model, we found that the *ped1*-deficient phage PaoP5Δ*ped1* exhibited substantially attenuated bactericidal activity against PAO1 *in vivo* (**Fig. EV5F**). Collectively, these findings indicate that Ped1 makes a critical contribution to enhancing host lysis efficiency throughout phage infection.

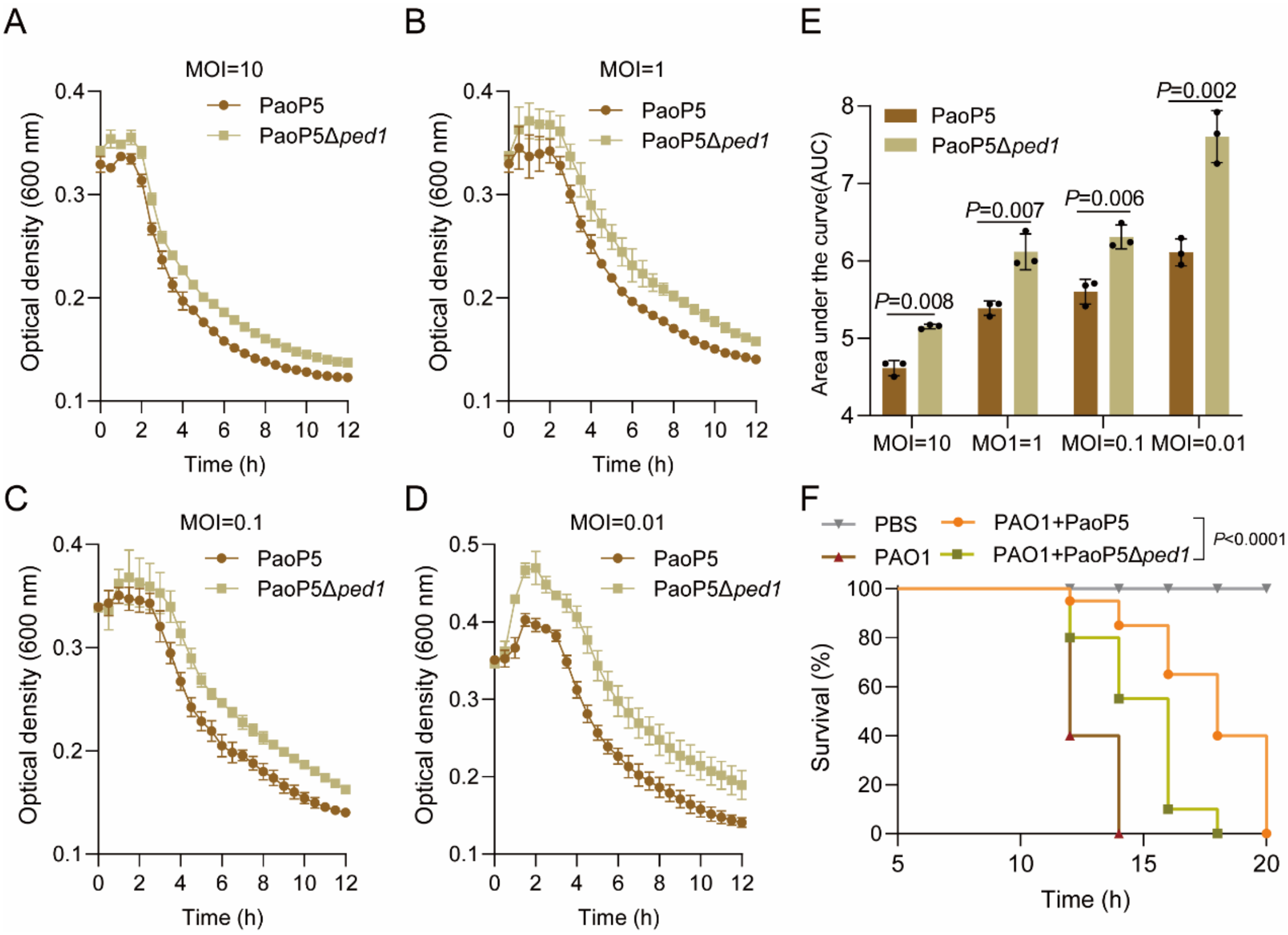

Figure EV5. Deletion of *ped1* significantly compromises phage infectivity. (A-D) Bacterial lysis kinetics of *P. aeruginosa* PAO1 infected with PaoP5 or PaoP5Δ*ped1* at MOIs of 10, 1, 0.1 and 0.01. Bacterial density (OD_{600}) was monitored every 30 minutes for 12 hours post-infection. **(E)** Quantification of the area under the curve (AUC) for *A-D*. **(F)** Survival curves of *Galleria mellonella* larvae infected with PAO1 and treated with PBS, wild-type PaoP5, or PaoP5Δ*ped1*. n=20. Error bars: mean ± SD (*A-E*). Statistical significance was determined by unpaired t-test (*E*). Statistical significance was calculated using log-rank test (*F*).

Minor comments

Line 168, Show the AlphaFold 2/3 models

Response: We thank the reviewer for this important concern. Models predicted by AlphaFold 2/3 were already shown in **Figure EV3A**, they were aligned with Ped1 crystal structure

Please add a sequence alignment for all the Ped1 homologs in Fig. 6a, indicate also the proposed catalytic residues, these must be conserved.

Response: We appreciate your valuable comments. Trp58 is strictly conserved across the analyzed sequences, whereas Ser32 is only relatively conserved (**Appendix Figure S6A**).

Other comments

The references given in the text are not acceptable, the first authors should be indicated clearly.

Response: We have revised the formatting of all references in accordance with the journal's requirements.

Line 94. The term „lytic virus" is wrong, it is a virulent virus.

Response: Revised as suggested.

Dnase I should be DNase I.

Response: We have made corrections throughout the manuscript.

Fig. 1b, at which IPTG concentration was this recorded, how does it correlate to Fig. 1a, keep here in mind related to the title of the results paragraph, that the cells are growing, hence Ped1 has at this stage no effect on the cell growth but division.

Response: To clarify the distinct readouts, we have added the IPTG concentration (1 mM) to the **Figure 1B** legend. As shown, **Figure 1A** reports growth curves (OD_{600}), whereas **Figure 1B and 1C** present morphological changes (cell length). Together, these results consistently demonstrate that Ped1 affects cell growth by inhibiting cell division.

"Bioinformatic analysis showed that Ped1 shares no significant sequence similarity with any protein studied before." This strong statement needs some data or being rephrased.

Response: Thank you for your helpful suggestion. We have revised the description as "BLAST and the conserved domain (CD) searches revealed that Ped1 lacks any detectable similarity to known functional proteins and contains no identifiable conserved domains." (Lines 106-108).

Please give the column used in SEC-MALS and comment about the different elution compared to Fig. S5b.

Response: We thank the reviewer for this question. The SEC-MALS analysis was performed using a Superdex 200 Increase 10/300 GL column (Cytiva), which is the same column type used for the conventional SEC analysis shown in **Fig. S5B (Now: Appendix Figure S2B)**. This column has a separation range of approximately 10 kDa to 600 kDa, making it suitable for analyzing the DnaB hexamer (~300 kDa) and its complexes with Ped1. And the result shown in **Fig. S5B (Now: Appendix Figure S2B)** is not from SEC-

MALS, but SEC-HPLC. SEC-HPLC is not the gold standard to measure the molecular weight of biomacromolecules, which are affected by several factors. The factors include shape mismatch, calibration curve dependence, non-ideal column interactions, glycosylation, concentration and loading effects, temperature and flow rate and so on. But none of these affect SEC-MALS, which measures absolute MW from light scattering independent of elution position.

Referee #2:

Wang et al. report the discovery and characterisation of a phage-encoded protein, Ped1, which acts as a nuclease and blocks DNA replication during infection of Pseudomonas aeruginosa. Ped1 was previously identified as an inhibitor of Pseudomonas, and here is characterised structurally and biochemically. The introduction provides an informative and balanced account of the mechanisms used by phages to subvert host physiology in Pseudomonas. Overexpression of fluorescent Ped1 causes a filamentation phenotype (Fig1), which correlates with the DNase activity of the protein in vivo and in vitro. The X-ray structure reveals a novel fold and conserved active site residues are identified (Fig 2). Ped1 is shown to interact specifically with the hexameric replicative helicase DnaB (Fig 3). This interaction inhibits DnaB helicase activity and also inhibits Ped1 nuclease activity. It should be said that the evidence for DnaB helicase inhibition is rather weak and could be strengthened if this claim is considered significant.

Overall, this is an interesting body of work that brings many different techniques to bear on the function of the Ped1 nuclease during phage infection. The data for Ped1 nuclease activity are solid, as is the interaction with DnaB (although stoichiometry is unclear as written). The evidence that DnaB is inhibited by Ped1 binding is rather weak and needs to be strengthened if this remains a central claim of the paper.

Response: We sincerely appreciate your positive evaluation for our manuscript. We have performed the following additional experiments to address these concerns. **(1)** Quantitative analysis via isothermal titration calorimetry (ITC) using purified DnaB and Ped1 revealed a Kd of 2.7±0.35 μM (**Fig. 3B**), which is comparable to the protein-protein binding parameters reported previously (Gao *et al*., 2026, *Nat Commun*. 17(1): 4150; Antar *et al*., 2023, *Commun Biol*. 6(1):1204). **(2)** We examined intracellular DNA replication efficiency in bacteria expressing wild-type Ped1, Ped1$^{S32L/W58L}$, or Ped1$^{Y69A/Y71A}$. The results showed that Ped1 binding to DnaB leads to a significant decrease in bacterial replication efficiency (**Fig. 3F**). **(3)** We performed the helicase assays with increasing concentrations of Ped1$^{S32L/W58L}$ *in vitro* and observed a dose-dependent inhibition of DnaB enzymatic activity by Ped1 (**Figs. 3E and Fig. EV4G**). **(4)** In the conditional *dnaB* knockdown strain (Δ*dnaB*(pHerd20T-*dnaB*)), only the DNase-deficient mutant that retains DnaB binding

(Ped1$^{S32L/W58L}$) and inhibits bacterial growth, whereas the binding-deficient mutant (Ped1$^{Y69A/Y71A}$) did not. Collectively, these results clearly support that Ped1 inhibits DnaB helicase function through direct binding.

Specific points:

1. Line 152. Km should be reported in units of concentration, eg nM and Vmax in reciprocal time.

Response: The protein binding parameters have been revised to: Km =9.90 nM, Vmax = 5.78 nM/min. Corresponding updates have been made in the Results and Methods sections.

2. For the kinetic analysis, the methods do not describe the conditions of the nuclease assay used - in particular the substrate and enzyme concentrations. Have steady-state, multiple turnover conditions been applied?

Response: Thanks for your suggestions. The reaction was performed at 37°C using λ DNA (0.5 μg/μL) as substrate and 0.01 mM enzyme. Aliquots were collected within every 5 min to ensure steady-state and multiple-turnover conditions.

3. In figure S2, a Michaelis-Menten plot is mentioned in the figure legend, but not shown.

Response: We apologize for the confusion. What we intended to present was the Lineweaver–Burk plot, not the Michaelis–Menten plot, and the description has been corrected.

4. In figure S2m, n, means of duplicate measurements are shown {plus minus} SD. Statistical analysis cannot be carried out with less than triplicate measurements.

Response: The experiments shown are three independent biological replicates. We have updated the figures accordingly, and present as the mean ± SD (Fig. EV2M-N).

5. In figure S2o, error bars are suggested but seem to be absent.

Response: We apologize for the lack of clarity in our original presentation. The figure shows a Lineweaver–Burk plot, in which each data point represents an individual calculated value rather than the mean of replicate measurements. Therefore, error bars are not applicable. To avoid confusion, we have revised the corresponding text and figure legend in the revised manuscript.

6. Please submit the structure to the DALI server, which is the gold standard for structural comparisons, and report the most significant matches. Does the core topology match any known nuclease superfamily? Is the enzyme a monome? - what does PISA analysis of the structure indicate?

Response: We thank the reviewer for this important concern.

(1) Thank you for your question. We submitted the Ped1 crystal structure (query chain s001A) to the Dali server, which returned 86 hits ranked by Z-score. The top hit, 9jp6-A (annotated "nuclease encoded by gene PaoP5_157"), corresponds to Ped1 itself (Z = 17.2, RMSD = 0.1 Å, 100% sequence identity) and therefore represents the query structure rather than a structural homolog. Excluding this self-match, the highest Z-score was only 3.9 (6uek-A, urocanate hydratase; and 6ywc-C, an antibody heavy chain); no hit reached Z ≥ 4, only four hits fell in the range 3.5 ≤ Z < 4, and the remaining 81 hits had Z-scores between 2 and 3.5. All of these matches are at the noise level: they share only 4–15% sequence identity, superpose with high RMSD (2.0–3.9 Å) over short alignments (lali = 43–58 residues), and correspond to functionally unrelated proteins spanning disparate families (e.g., urocanate hydratase, an antibody fragment, ClpS, a PI3K regulatory subunit, CRISPR/Cas proteins, and various polymerases and transporters). Such a distribution—uniformly low Z-scores, negligible sequence identity, and no coherent structural family—is the pattern expected in the absence of any genuine structural homolog.

DALI aligns structures based on inter-residue distance matrices, which is powerful but does not strictly enforce sequence continuity. Alignments reported by DALI can sometimes reflect opportunistic geometric similarity rather than a physically meaningful folding equivalence, especially when long loops or rearrangements bring distant segments into proximity. The heuristic search and scoring model can miss some significant structural relationships or produce ambiguous alignments, particularly for non-canonical folds. Therefore, a high DALI Z-score or apparently good superposition should be interpreted with caution, ideally corroborated with additional evidence (e.g., sequence conservation, functional data, other alignment methods). For DALI topology alignment analysis, significant alignments typically have Z-scores>2, but confident evolutionary/functional matches (strong matches) often have much higher Z-scores (generally > 8–10) *(Dietmann et al., 2001, Nucleic Acids Res 29:55-57* and *Holm et al.,1999, Nucleic Acids Res 27:244-247*). Holm, L. and *et. al* mentioned in their study that "Thresholds vary between protein families... One should look at a combination of structural, sequence and functional conservation to infer an evolutionary relationship." (*Dietmann et al., 2001, Nucleic Acids Res 29:55-57* and *Holm et al., 1999, Nucleic Acids Res 27:244-247*).

According to the standard Dali criteria (Z-scores below 2 are considered spurious, and Z-scores of roughly ≥ 8 are generally required to indicate homology), Ped1 has no credible structural homolog in the PDB, as all non-self matches fall within the noise range. This

provides independent support for our conclusion that Ped1 adopts a previously undescribed α/β-nuclease fold and can be classified as an orphan nuclease. This result is fully consistent with, and corroborates, our Foldseek search, which likewise identified no experimentally determined structural homologs.

(2) We analyzed the Ped1 crystal structure with PISA to assess whether it forms a stable oligomeric assembly. PISA reported the only stable assembly present in the coordinates as multimeric state 1, with the formula Aab2 and composition A·[CL]·$[GOL]_2$—*i.e.*, a single protein chain (A) together with one chloride ion (CL) and two glycerol molecules (GOL). Thus, PISA assigns Ped1 as a monomer in the crystal, with no stable protein–protein interface; the only "interface" contacts involve crystallization additives (glycerol and chloride) rather than a biologically meaningful protein interface. Consistent with this assignment, the interface is energetically weak. Although the buried area is ~612 $Å^2$ with ΔG_int ≈ −10.4 kcal/mol, the dissociation free energy is only ΔG_diss ≈ +0.8 kcal/mol (TΔS_diss ≈ 0.8 kcal/mol), and the predicted dissociation pattern (A·[CL]·[GOL] + [GOL]) corresponds simply to the loss of a glycerol molecule. A ΔG_diss of only ~0.8 kcal/mol indicates that this assembly is essentially unstable in solution and does not represent a genuine biological oligomer. We therefore conclude that the Ped1 crystal structure contains no stable protein–protein oligomerization interface and that Ped1 itself is monomeric; the apparent "complex" is composed solely of solvent/additive molecules (glycerol and chloride) and has no biological significance.

7. With a single active site, Ped1 would be expected to nick dsDNA in the first instance. This could be tested by incubating the protein with a supercoiled plasmid and looking for relaxed products at early time points.

Response: Thanks for this valuable suggestion. We performed DNase assay using supercoiled pUCP20 plasmid DNA as the substrate (**Fig. EV2D**). Following a 3-min incubation with Ped1, nicked open circular DNA was readily detected together with a proportion of linear DNA, indicating that Ped1 initially introduces nicks into supercoiled DNA that can subsequently progress to linearization. These findings further support the endonuclease activity of Ped1.

8. In figure S4, parts d and e are mis-labelled.

Response: We have corrected the mislabeling in Fig. S3D and E (**Fig. EV3D and E**) as indicated.

9. Figure S5a - clarify what label # signifies. How often was this experiment carried out?

Response: The symbol "#" indicates the number of identified peptides. The Co-IP experiment was performed twice, and mass spectrometry analysis was carried out once.

10. Figure S5e - the gels showing purity of proteins are heavily cropped. Please provide uncropped gels to allow the reader to assess the purity objectively.

Response: Thanks for your comment. The uncropped original gel images have been supplemented as a separate file (**Source Data files**) in submission system.

11. Line 220 - what does "recruits" mean in this context? Do you mean "binds"?

Response: We appreciate your comment. The term "recruits" was intended to indicate that Ped1 associates with DnaB through direct binding. To avoid ambiguity, we have replaced "recruits" with "binds" throughout the revised manuscript where appropriate.

12. Line 220 - is a Kd of 3.2 μM high affinity for a protein:protein interaction? Can the authors provide some examples of other high affinity protein interactions with similar Kds in the literature?

Response: We thank you for raising this important point. We acknowledge that a Kd of 3.2 μM falls within the moderate to moderately high affinity range for protein-protein interactions, rather than the nanomolar range typically associated with ultra-high affinity. Nevertheless, this level of affinity is commonly observed in a variety of physiologically relevant protein-protein and protein-ligand interactions. For comparison, we surveyed the literature and compiled published Kd values (all in the μM range) determined by ITC for protein-protein interactions.

(1) Gao S, Wang L, Yao L, Mao Y, Eisenstein M, Soh HT, Zheng L. Aptamers with magnetically tunable affinity for divalent cobalt ions. ***Nat Commun***. 2026, 17(1):4150.
(2) Antar H, Gruber S. VirB, a transcriptional activator of virulence in Shigella flexneri, uses CTP as a cofactor. ***Commun Biol***. 2023, 6(1):1204.

13. Figure 3 and accompanying text. DnaB is a hexamer and Ped1 is suggested to be a monomer. Do the ITC data support a 1:1 (ie 6:6) complex of the two proteins? How does this fit with figure 3f which shows a mass for the complex of 300 kDa -roughly equal to 6 DnaB subunits but not a 6:6 complex?

Response: We thank the reviewer for this critical question regarding the stoichiometry of the Ped1-DnaB interaction. In order to confirm the DnaB:Ped1 stoichiometry, except SEC-MALS , we also tried quantitative mass imaging of single biological macromolecules by mass photometry (*Young et al*, 2018, *Science* 360: 423-427). The mass photometry

analysis of the purified DnaB-Ped1 complex resolved three populations. The dominant species (329 ± 58 kDa; 70% of counts) corresponds closely to the calculated mass of a DnaB hexamer bound to a single Ped1 molecule (6 × 52.7 + 9.5 = 325.7 kDa). This is not a 6:6 complex, which would have a calculated mass of approximately 6 × (52.7 + 9.5) ≈ 373 kDa. The observed 329 kDa species is therefore basically consistent with a 1 Ped1 : 1 DnaB hexamer stoichiometry. However, the additional minor populations observed—117 ± 39 kDa (19%) and 614 ± 99 kDa (9%)—likely represent sub-hexameric DnaB species or dimeric complexes, reflecting the inherent compositional heterogeneity of the protein preparation rather than multiple physiologically relevant binding modes **(Appendix Fig. S2F**). The compositional heterogeneity, together with peak widths (σ) that are large relative to the mass of a single Ped1 molecule (~9.5 kDa), precludes assigning a single precise DnaB:Ped1 stoichiometry. We already substitute SEC-MALS result with mass photometry result and revised relevant contents (Lines 212-220, 367-375).

14. For the ITC experiments, more details of the experimental design are required. What was the fitting model used? Were other models also tested? How often was this experiment repeated - it looks like a single time from the text.

Response: Thanks for your suggestion. We have revised the Materials and Methods section to provide additional details of the ITC experiments as "The binding of Ped1 to DnaB was characterized by isothermal titration calorimetry using a MicroCal PEAQ-ITC instrument (Malvern Panalytical) at 25 °C with a reference power of 5. The sample cell was loaded with DnaB at a final concentration of 50 μM, and the injection syringe contained Ped1 at 300 μM; both proteins were prepared in the buffer containing 20 mM HEPES 150 mM NaCl (pH 7.5) to minimize heats of dilution. The titration comprised 19 injections in total: an initial 0.4 μL injection delivered over 0.8 s, followed by 18 injections of 2 μL each delivered over 4 s, with an interval of 120 s between consecutive injections to allow the thermal power signal to return to baseline. The first injection (0.4 μL) was excluded from the analysis to correct for ligand diffusion across the syringe tip during the pre-titration equilibration period. The heat evolved by each injection was integrated, normalized per mole of injectant, and plotted against the molar ratio of Ped1 to DnaB. The resulting binding isotherm was fit to a one-site binding model to determine the dissociation constant Kd using the MicroCal PEAQ-ITC Analysis Software (Malvern Panalytical)." (Lines 617-630). This experiment was performed three times using MicroCal PEAQ-ITC, and Fig. 3B shows a representative result from one of the three independent experiments. The results of all three replicates are shown in the figure below.

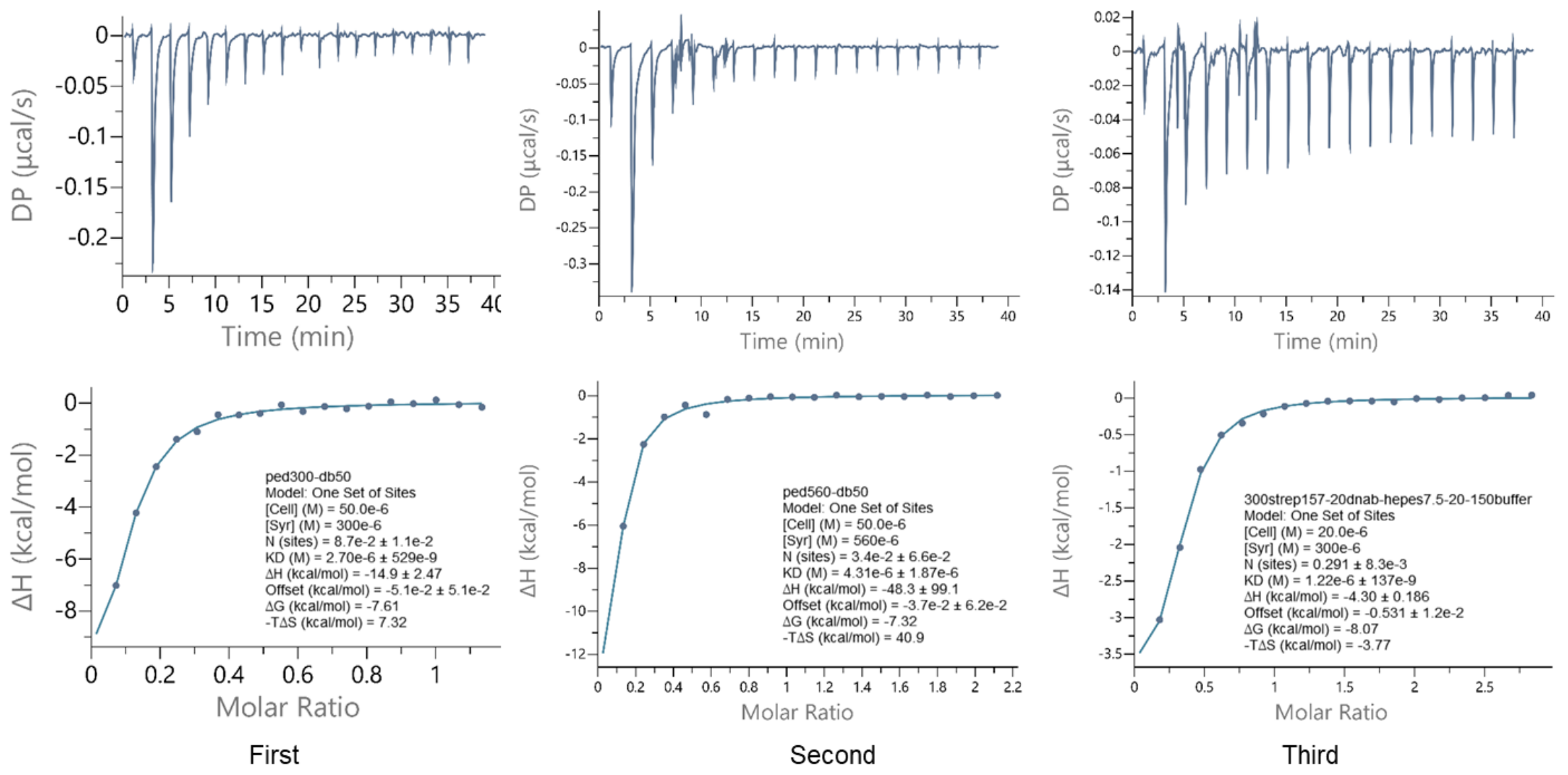

<u>Fig SS3.</u> ITC analysis of the DnaB-Ped1 interaction. Thermogram shows raw heat signals (upper) and integrated binding isotherm (lower) from titrating DnaB into Ped1. Data were fitted using a one-site binding model.

15. Figure 3e - DnaB unwinding assay. It is possible that the variant Ped1 used here can still bind to ssDNA and dsDNA. This could result in inhibition without requiring an interaction with DnaB. Can the authors please test this possibility?

Response: We thank you for your valuable concern. To test whether the Ped1 variant used in the unwinding assay could still bind DNA and thereby cause inhibition indirectly, we performed EMSA by incubating Ped1$^{S32L/W58L}$ with forked DNA in the unwinding assay (**Fig. SS4**). The results demonstrated that Ped1$^{S32L/W58L}$ does not bind to either ssDNA or dsDNA. Therefore, the inhibition of DnaB helicase activity is indeed achieved through direct binding of Ped1 to DnaB, rather than through competition for DNA substrates.

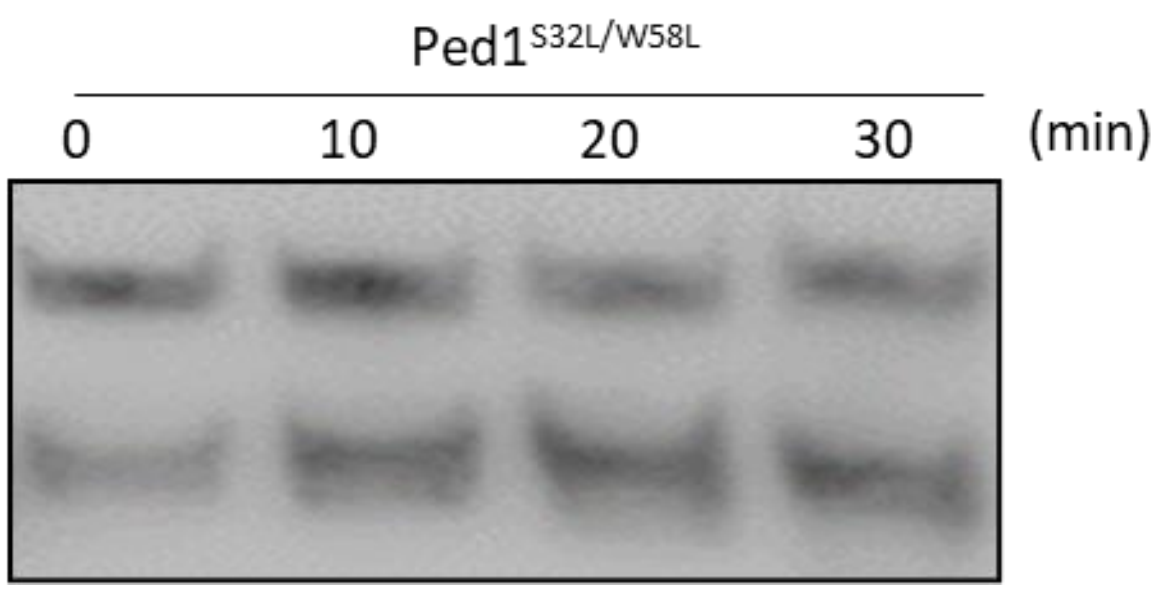

<u>Fig SS4</u>. Electrophoretic mobility shift assays (EMSA) were performed by incubating Ped1$^{S32L/W58L}$ with single-stranded and double-stranded DNA. Over the time course examined, no binding of Ped1$^{S32L/W58L}$ to either dsDNA or ssDNA was observed, indicating that the mutant lacks nonspecific DNA-binding activity.

16. The methods section suggests that the wt Ped1 protein was also tested with DnaB in the unwinding assay, but this data is not shown. Can the authors clarify please?

Response: We have performed the helicase assay using wild-type Ped1 before the last manuscript was completed. However, the results showed that wild-type Ped1 degraded the DNA substrate due to its intrinsic DNase activity, which complicated the interpretation of the unwinding assay (**Fig. SS5**). We therefore preferred to use the catalytically inactive mutant for this experiment.

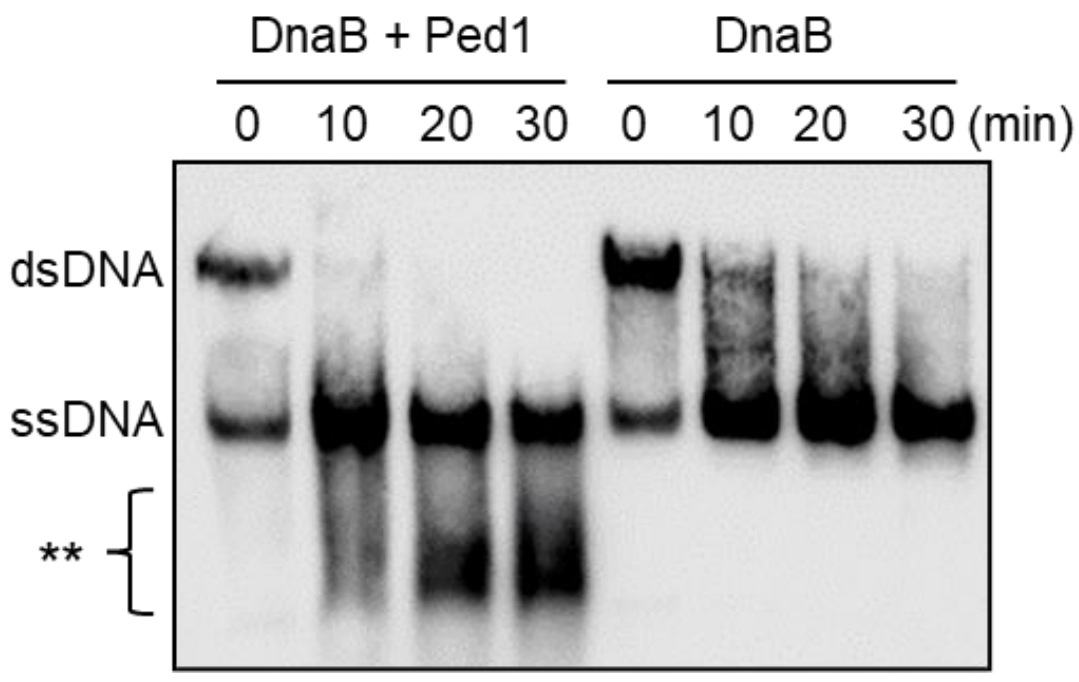

Fig SS5. Unwinding of biotin-labelled forked-duplex DNA substrate in the presence of DnaB or DnaB with Ped1. DnaB proteins were incubated with the fork DNA substrates at 37℃ for 30 minutes, then forked (dsDNA) and unwound (ssDNA) DNA were analyzed by polyacrylamide gel electrophoresis. The double asterisk represents the degraded fragments of fork DNA.

17. For the unwinding assay, why were purified Ped1-DnaB complexes assayed rather than adding increasing concentrations of Ped1 to DnaB in the assay? These experiments would provide important further confirmation of this inhibition.

Response: As suggested, we have now titrated increasing amounts of Ped1$^{S32L/W58L}$ into the equal amounts of the DnaB helicase activity assay reaction. The results revealed a clear dose-dependent inhibition of DnaB activity (**Fig. EV4G-H**), and we have incorporated this data into the revised manuscript.

18. Lines 350-355. These comments are very speculative and founded on rather sparse evidence, so should be toned down or removed.

Response: We appreciate your suggestions. We have removed the corresponding descriptions from the Discussion section to tone down the speculative interpretations.

19. the model in figure 6 requires that Ped1 and DnaB are expressed at similar levels in infected cells. Is there any evidence for this? If not, this issue needs to be discussed and

caveated.

Response: Thanks. To determine whether Ped1 and DnaB are expressed at comparable levels in bacterial cells, we performed qRT-PCR analysis during PaoP5 infection. Results showed that at most of the infection cycle, the mRNA level of *ped1* was approximately two-fold higher than that of *dnaB* (**Appendix Figure S3**). Subsequently, Ped1 expression decreased to a level similar to that of *dnaB* within 8-15 minutes post-infection. These findings support the model that Ped1 and DnaB are co-expressed at physiologically relevant levels throughout phage infection, allowing for their interaction.

20. Figure 6. What does "evolutionary decoy" mean? Where is evolution coming into this mechanism, other than the fact that all the players co-evolve, which is true for everything in this context. Likewise, the term "strategic highjacker" seems unnecessary and anthropomorphic.

Response: We thank the reviewer for this thoughtful comment. We agree that the terms "evolutionary decoy" and "strategic hijacker" may overinterpret our findings and introduce unnecessary anthropomorphic language. Our intention was to illustrate a conceptual model of how Ped1 exploits the host DNA replication machinery rather than to imply that we have directly demonstrated an evolutionary process or adaptive strategy. To avoid overstatement, we have revised the corresponding Discussion (Lines 413-416). Specifically, "evolutionary decoy" has been replaced with "functional mimic", and "strategic hijacker" has been replaced with "phage-encoded effector" We have also revised the accompanying text to present the model in a more neutral and mechanistic manner.

21. Many phage express DNA nucleases that degrade host DNA to provide nucleotides for phage DNA synthesis. This should be discussed in a paragraph in the discussion to place the work in context.

Response: As suggested, we have added a new paragraph in the Discussion section to compare Ped1 with other well-characterized phage nucleases (Lines 393-400). We compared the substrate specificity and mode of action among these nucleases. Notably, Ped1 additionally inhibits host DNA replication through DnaB interaction, a feature is absent from the other nucleases.

Referee #1:

"The authors improved the manuscript significantly. However, I find that still the story tries to put unnecessarily all in. I am still not convinced by the interaction between Ped1 and DnaB, a simple shift in an EMSA is not enough validation for direct interaction, it could be also an indirect effect. If the authors cannot provide evidence for direct interaction, the text must be revised. The claim about the direct interaction between Ped1 and DnaB is not required for the story and does not add much."

We thank this reviewer for the positive evaluation of our revisions and for giving us the opportunity to clarify our findings regarding the Ped1-DnaB interaction. We respectfully acknowledge the reviewer's perspective, but we would like to clarify a methodological distinction, as well as highlight the comprehensive orthogonal evidence already present in our manuscript:

1) Clarification on Methodology: EMSA (Electrophoretic Mobility Shift Assay) is primarily designed to characterize protein–nucleic acid interactions.

2) In our study, we evaluated the protein-protein interaction (PPI) between Ped1 and DnaB including the following data:

a) *In Vitro* using Size-Exclusion Chromatography (SEC) (Appendix Fig. S2B), where a distinct peak shift of the co-incubated purified proteins directly reflects stable physical complex formation in solution. To provide quantitative, direct proof of physical binding without potential cellular or nucleic acid intermediaries, we conducted ITC using independently purified Ped1 and DnaB. Our ITC results yielded a dissociation constant (Kd) of 2.7±0.35μM (Fig. 3B), demonstrating a robust and direct physical interaction between Ped1 and DnaB *In Vitro*.

b) In addition to SEC and ITC, the direct physical and functional interaction between Ped1 and DnaB is supported by a comprehensive array of complementary biochemical, genetic, and physiological assays: GST pull-down assays confirmed the direct binding between purified GST-Ped1 and DnaB (Appendix Fig. S2C). Bacterial Two-Hybrid (BACTH) demonstrated direct interaction within a live bacterial cell environment (Appendix Fig. S2D).

c) *In Vitro* Functional Reciprocation: Purified Ped1 directly inhibits DnaB helicase unwinding activity in a dose-dependent manner (Fig. 3E and Fig. EV4F–H). Reciprocally, purified DnaB directly neutralizes Ped1's DNase digestion activity *In Vitro* (Fig. 4A).

d) Expression of Ped1's nuclease-dead variant ($Ped^{S32L/W58L}$) still inhibits bacterial growth, whereas nuclease-dead/binding-deficient mutants ($Ped1^{Y69A/Y71A}$) fail to cause toxicity (Fig. 3C-D). Furthermore, co-expressing DnaB completely rescues the Ped1-induced growth inhibition, cell filamentation, and genomic DNA depletion (Fig. 4B–F).

Taken together, these multi-layered biochemical and physiological lines of evidence conclusively establish that Ped1 and DnaB directly interact and act as mutual functional antagonists.

Referee #2:

"The authors have responded constructively to my comments and concerns. They have provided significant new data, revised analysis in places and revised the text where needed. I agree with referee 1 that this ia an unusual nuclease and the evidence for a conserved active for metal-dependent nuclease activity is weak. SDM of highly conserved putative active site residues (likely charged ones) would still significantly strengthen the paper. I support publication with this

reservation; however, if referee 1 is unsatisfied with the attempts to conclusively prove nuclease activaty for Ped1 then my comments could be relevant.”

We deeply appreciate Referee #2’s positive evaluation, recognition of our efforts, and support for publication. Regarding the comment on the nuclease active site and Site-Directed Mutagenesis (SDM): We note that Referee #1’s primary comment in this round focused on the direct interaction between Ped1 and DnaB (which were addressed in detail using our ITC, SEC, and pull-down data and etc) rather than questioning the intrinsic nuclease activity or metal dependency of Ped1.

As demonstrated in our manuscript, Ped1 represents a novel, orphan α/β-nuclease fold with no previously characterized structural homologs (Data Set EV1). Mutating key active-site pocket residues (S32L, W58L, and the double mutant S32L/W58L) almost abolished Ped1's Mg^{2+}-dependent DNase activity *in vitro* (Fig. 2D) and abolished its ability to deplete intracellular DNA and induce host filamentation *in vivo* (Fig. 2E–G). In addition, the nuclease activity was confirmed using *in vitro* synthesized protein (PURExpress system) to exclude bacterial host contamination (Fig. EV1E–F), and showed strict EDTA-inhibitable, Mg^{2+}-dependent cleavage (Fig.1E). Therefore, we believe these comprehensive data fully support our conclusions regarding Ped1’s dual mechanism of action, and we thank the referee again for supporting the publication of our work.

1st Revision - Editorial Decision **1st Sep 2026**

Dear Haihua,

Thank you for submitting a revised version of your manuscript to The EMBO journal. Your study has now been seen by two of the original referees, who find that most of their previous concerns have been sufficiently addressed, while indicating that additional evidence would be needed to support the direct interaction of Ped1 with DnaB and for the identification of the metal-dependent nuclease site in Ped1. Please address these concerns in the final version with appropriate caveating in the manuscript text.

In addition to the remaining reviewer requests, there are a few editorial points that need to be addressed before I can extend the official acceptance of the manuscript:

1. Please note that it is The EMBO Journal policy for the transcript of the editorial process (containing referee reports and your response letters) to be published as an online supplement to each paper. If you should prefer removal of any referee-only figures included in the point-by-point response(s), e.g. because they may still be used for future publication or because they have been reproduced from published work by others, please do let us know immediately via response email.
More information is available here: https://www.embopress.org/transparent-process#Review_Process
2. Please update the order and the headings of the manuscript sections as follows: Abstract / Introduction / Results / Discussion / Methods / Data Availability / Acknowledgements / Disclosure and Competing Interests Statement / References / Figure Legends / Expanded View Figure Legends.
3. CRediT has replaced the traditional author contributions section because it offers a systematic, machine-readable author contributions format that allows for more effective research assessment. Please remove the Authors Contributions from the manuscript and use the free text boxes beneath each contributing author's name in our online submission system to add specific details on the author's contribution. More information is available in our guide to authors.
4. In the Data Availability section, please replace the current statement with "This study includes no data deposited in external repositories". Further information on the format of this section can be found at https://link.springer.com/partners/embo-press/editorial-policies#Data%20availability%20statement.
5. Please update references according to The EMBO Journal style - where there are more than 10 authors on a paper, the first 10 should be listed, followed by 'et al.'
6. Figure panels 2A,B are not mentioned in the manuscript text, please check and correct.
7. In the Reagent Table, please update citations to "Dataset EV5" etc.
8. Please correct the typo in Figure EV1B ("Bright field").
9. In the Appendix, please add page numbers to table of contents and upload final version as a PDF labeled "Appendix".
10. During our standard image check, we noticed possible reuse between the figure panels in Figure 1B and Figure EV1 C. Please check - if this was intentional, please clearly state this in the corresponding figure legends.
11. There are several issues with source data that require your attention:
- Please submit the raw, unprocessed images for all microscopy images.
- Source data for Figure 3A do not appear to fit to the main figure panel.
- Source data for Fig 2D contain pixellation artifacts that are not present in the figure itself.
- Source data for Figure 3B are missing.
12. Our data editors have flagged the following issues in figure legends that need correcting:
- Please provide the exact p values in the legends of 1A,C; 2F,G; 3D; 4C,F; 5A; EV-1D; EV-4A; EV-5F; Appendix Figure S1-B.
- Please indicate the statistical test used for data analysis in the legends of figures 5C,D; Appendix Figure S1-B,D; S2-D; S4-B,C.
- Please define the error bars in the legends of figures 2F,G; 3D,F.
- Please note that scale bar and its definition are missing for figure 4B.

Please let me know if you have any questions regarding any of these points. You can use the link below to upload the revised files.

Thank you again for giving us the chance to consider your manuscript for The EMBO Journal. I look forward to receiving the final version.

With best wishes,

Ieva

Ieva Gailite, PhD
Senior Scientific Editor
The EMBO Journal
Meyerhofstrasse 1
D-69117 Heidelberg

i.gailite@embojournal.org

We realize that it is difficult to revise to a specific deadline. In the interest of protecting the conceptual advance provided by the work, we recommend a revision within 3 months (30th Nov 2026). Please discuss the revision progress ahead of this time with the editor if you require more time to complete the revisions. Use the link below to submit your revision:

https://emboj.msubmit.net/cgi-bin/main.plex

Referee #1:

The authors improved the manuscript significantly. However, I find that still the story tries to put unnecessarily all in. I am still not convinced by the interaction between Ped1 and DnaB, a simple shift in an EMSA is not enough validation for direct interaction, it could be also an indirect effect. If the authors cannot provide evidence for direct interaction, the text must be revised. The claim about the direct interaction between Ped1 and DnaB is not required for the story and does not add much.

Referee #2:

The authors have responded constructively to my comments and concerns. They have provided significant new data, revised analysis in places and revised the text where needed.
I agree with referee 1 that this ia an unusual nuclease and the evidence for a conserved active for metal-dependent nuclease activity is weak. SDM of highly conserved putative active site residues (likely charged ones) would still significantly strengthen the paper.
I support publication with this reservation; however if referee 1 is unsatisfied with the attempts to conclusively prove nuclease activaty for Ped1 then my comments could be relevant

Dear editor and reviewers:

We are deeply grateful to the editor and the reviewers for their constructive comments and careful evaluation of our manuscript "**A dual-action bacteriophage protein Ped1 degrades DNA and blocks replication to subvert *Pseudomonas*" (ID: EMBOJ-2026-124104).** All concerns have been carefully addressed, and the manuscript has been revised according to the reviewers' suggestions. Our point-by-point responses follow below.

Response to editor:

1. Please note that it is The EMBO Journal policy for the transcript of the editorial process (containing referee reports and your response letters) to be published as an online supplement to each paper. If you should prefer removal of any referee-only figures included in the point-by-point response(s), e.g. because they may still be used for future publication or because they have been reproduced from published work by others, please do let us know immediately via response email. More information is available here:https://www.embopress.org/transparent-process#Review_Process

Response: We confirm that publication as an online supplement is suitable and raises no concerns.

2. Please update the order and the headings of the manuscript sections as follows: Abstract / Introduction / Results / Discussion / Methods / Data Availability / Acknowledgements / Disclosure and Competing Interests Statement / References / Figure Legends / Expanded View Figure Legends.

Response: Revised as requested.

3. CRediT has replaced the traditional author contributions section because it offers a systematic, machine-readable author contributions format that allows for more effective research assessment. Please remove the Authors Contributions from the manuscript and use the free text boxes beneath each contributing author's name in our online submission system to add specific details on the author's contribution. More information is available in our guide to authors.

Response: We have moved the Authors Contributions from the manuscript.

4. In the Data Availability section, please replace the current statement with "This study includes no data deposited in external repositories". Further information on the format of this section can be found at https://link.springer.com/partners/embo-press/editorial-policies#Data%20availability%20statement.

Response: Revised the Data Availability as: "All sequencing data have been uploaded to BioProject under accession number PRJNA1256806. This study includes no data deposited in external repositories."

5. Please update references according to The EMBO Journal style - where there are more than 10

authors on a paper, the first 10 should be listed, followed by 'et al.'

Response: Revised as requested.

6. Figure panels 2A, B are not mentioned in the manuscript text, please check and correct.

Response: We have verified that Fig. 2A and 2B are indeed mentioned in the text (**Page 7, Line 170**).

7. In the Reagent Table, please update citations to "Dataset EV5" etc.

Response: Revised as requested.

8. Please correct the typo in Figure EV1B ("Bright field").

Response: We have corrected it.

9. In the Appendix, please add page numbers to table of contents and upload final version as a PDF labeled "Appendix".

Response: Revised as requested.

10. During our standard image check, we noticed possible reuse between the figure panels in Figure 1B and Figure EV1 C. Please check - if this was intentional, please clearly state this in the corresponding figure legends.

Response: We confirm that the images in Fig. 1B (0 h and 5 h) are indeed part of the same experimental series as those in Fig. EV1C. The rationale for this arrangement is that Fig. 1B serves to highlight the representative morphological change at the two most informative time points (before and after induction), whereas Fig. EV1C provides the complete temporal dynamics (0–5 h) as supplementary information.

To avoid any ambiguity, we have revised the legends of both figures to explicitly state the relationship as “**Fig 1B:** (B) Representative micrographs of PAO1 cells expressing Ped1 at 0- and 5-hours post induction with IPTG. Scale bar, 2.5 μm. The complete time-course (0-5 hours) is shown in Fig. EV1C” and “**Fig. EV1C:** (C) Time-course microscopy of cell filamentation. Micrographs of PAO1 harboring empty vector (Control) or expressing Ped1 taken at 1-hour intervals (0-5 hours) post-induction with IPTG. Scale bar, 2.5 μm. Images at 0 hour and 5 hours correspond to those presented in Fig. 1B.”

11. There are several issues with source data that require your attention:

- Please submit the raw, unprocessed images for all microscopy images.

Response: We have provided the raw, unprocessed images for all microscopy data as requested. These images are provided without strain/sample labeling or selection of regions, and the magnification has not been altered.

- Source data for Figure 3A do not appear to fit to the main figure panel.

Response: Sorry about for this confusion. The overall brightness and contrast of the images have

been adjusted and have now been made consistent.

- Source data for Fig 2D contain pixellation artifacts that are not present in the figure itself.

Response: We have now replaced the images with corrected versions.

- Source data for Figure 3B are missing.

Response: We have now included the original source data for ITC as requested.

12. Our data editors have flagged the following issues in figure legends that need correcting:

- Please provide the exact p values in the legends of 1A,C; 2F,G; 3D; 4C,F; 5A; EV-1D; EV-4A; EV-5F; Appendix Figure S1-B.

Response: We have reported the P values as $P < 0.0001$ for comparisons where the software output was below this threshold. The figure legends have been revised accordingly.

- Please indicate the statistical test used for data analysis in the legends of figures 5C,D; Appendix Figure S1-B,D; S2-D; S4-B,C.

Response: The statistical analysis methods applied are specified in the respective figure legends. For transcriptomic assay, the statistical analysis methods applied are specified in the methods section (lines 682-688).

- Please define the error bars in the legends of figures 2F,G; 3D,F.

Response: We have revised the manuscript accordingly, as requested.

- Please note that scale bar and its definition are missing for figure 4B.

Response: We have added the corresponding scale bars.

Response to Reviewers:

Referee #1:

"The authors improved the manuscript significantly. However, I find that still the story tries to put unnecessarily all in. I am still not convinced by the interaction between Ped1 and DnaB, a simple shift in an EMSA is not enough validation for direct interaction, it could be also an indirect effect. If the authors cannot provide evidence for direct interaction, the text must be revised. The claim about the direct interaction between Ped1 and DnaB is not required for the story and does not add much."

Response: We thank Referee #1 for the positive evaluation of our revisions and for giving us the opportunity to clarify our findings regarding the Ped1-DnaB interaction. We respectfully acknowledge the reviewer's perspective, but we would like to highlight the comprehensive orthogonal evidence already present in our manuscript, meanwhile, clarify a methodological distinction:

1) In our study, we evaluated the protein-protein interaction (PPI) between Ped1 and DnaB *In Vitro* using Size-Exclusion Chromatography (SEC) (Appendix Fig. S2B), where a distinct peak shift of the co-incubated purified proteins directly reflects stable physical complex formation in solution. To provide quantitative, direct proof of physical binding without potential cellular or nucleic acid intermediaries, we conducted ITC using independently purified Ped1 and DnaB. Our ITC results yielded a dissociation constant (Kd) of 2.7±0.35μM (Fig. 3B), demonstrating a robust and direct physical interaction between Ped1 and DnaB *In Vitro*. In addition to SEC and ITC, the direct physical and functional interaction between Ped1 and DnaB is supported by a comprehensive array of complementary biochemical, genetic, and physiological assays: *In Vitro* Pull-down Assays: GST pull-down assays confirmed the direct binding between purified GST-Ped1 and DnaB (Appendix Fig. S2C). *In Vitro* Functional Reciprocation: Purified Ped1 directly inhibits DnaB helicase unwinding activity in a dose-dependent manner (Fig. 3E and Fig. EV4F-H). Reciprocally, purified DnaB directly neutralizes Ped1's DNase digestion activity *In Vitro* (Fig. 4A).

In Vivo: Bacterial Two-Hybrid (BACTH): Demonstrated direct interaction within a live bacterial cell environment (Appendix Fig. S2D). Co-IP assays also clearly demonstrated that DnaB is a strong interacting partner of Ped1 (Fig. 3A).

In Vivo Phenotypic & Growth Recovery: Expression of Ped1's nuclease-dead variant ($Ped^{S32L/W58L}$) still inhibits bacterial growth, whereas nuclease-dead/binding-deficient mutants ($Ped1^{Y69A/Y71A}$) fail to cause toxicity (Fig. 3C-D). Furthermore, co-expressing DnaB completely rescues the Ped1-induced growth inhibition, cell filamentation, and genomic DNA depletion (Fig. 4B-F). Together, these multi-layered biochemical and physiological lines of evidence conclusively establish that Ped1 and DnaB directly interact and act as mutual functional antagonists.

2) EMSA (Electrophoretic Mobility Shift Assay) is primarily used to characterize protein–nucleic acid interactions. We did not use EMSA to analyze protein-protein interactions in our manuscript.

Referee #2:

"The authors have responded constructively to my comments and concerns. They have provided significant new data, revised analysis in places and revised the text where needed. I agree with referee 1 that this ia an unusual nuclease and the evidence for a conserved active for metal-dependent nuclease activity is weak. SDM of highly conserved putative active site residues (likely charged ones) would still significantly strengthen the paper. I support publication with this reservation; however, if referee 1 is unsatisfied with the attempts to conclusively prove nuclease activaty for Ped1 then my comments could be relevant."

Response: We deeply appreciate Referee #2's positive evaluation, recognition of our efforts, and support for publication. Regarding the comment on the nuclease active site and Site-Directed Mutagenesis (SDM): We note that Referee #1's primary comment in this round focused on the

direct interaction between Ped1 and DnaB (which were addressed in detail using our ITC, SEC, and pull-down data and *etc*) rather than questioning the intrinsic nuclease activity or metal dependency of Ped1.

As demonstrated in our manuscript, Ped1 represents a novel, orphan α/β-nuclease fold with no previously characterized structural homologs (Data Set EV1). Mutating key active-site pocket residues (S32L, W58L, and the double mutant S32L/W58L) almost abolished Ped1's DNase activity *in vitro* (Fig. 2D) and abolished its ability to inhibit bacterial growth, deplete intracellular DNA, and induce host filamentation *in vivo* (Fig. 2E-G). Biochemical & Ion-Dependency Assays: The nuclease activity was confirmed using *in vitro* synthesized protein (PURExpress system) to exclude bacterial host contamination (Fig. EV1E–F), and showed strict EDTA-inhibitable, Mg^{2+}-dependent cleavage (Fig.1E). We believe these comprehensive data fully support our conclusions regarding Ped1's dual mechanism of action, and we thank the referee again for supporting the publication of our work.

In addition, we have expanded the Discussion section to include a more detailed discussion of the unusual nuclease properties of Ped1, covering its incomplete digestion pattern, its nicking-only activity on supercoiled DNA, its novel α/β fold distinct from the HNH and PD-(D/E)XK superfamilies, and its strict conservation among *Pseudomonas* phages (lines 326-350).

2nd Revision - Editorial Decision 20th Sep 2026

Dear Haihua,

Thank you for incorporating the final editorial and formatting requests in the manuscript. I am now pleased to inform you that your manuscript has been accepted for publication. Congratulations on a nice study!

Before we forward your manuscript to our publishers, we would like to propose some edits in the manuscript title, abstract and synopsis. We have also selected a short blurb that will accompany the title of your manuscript in our online table of contents. Please take a look at the proposed textual changes in the attached text file - any corrections or edits from your side are very welcome.

Your manuscript will be processed for publication by EMBO Press. It will be copy edited and you will receive page proofs prior to publication. Please note that you will be contacted by Springer Nature Author Services to complete licensing and payment information.

You may qualify for financial assistance for your publication charges - either via a Springer Nature fully open access agreement or an EMBO initiative. Check your eligibility: https://link.springer.com/journal/44318/how-to-publish-with-us

Should you be planning a Press Release on your article, please notify the production team via this form in order to coordinate publication and release dates: https://forms.cloud.microsoft/e/eH4A8JVP6g

Once I have received your input on the final textual edits, I will be happy to forward your manuscript to our publishers for typesetting.

Thank you for this interesting contribution to The EMBO Journal!

Best wishes,

Ieva

Ieva Gailite, PhD
Senior Scientific Editor
The EMBO Journal
Meyerhofstrasse 1
69117 Heidelberg
i.gailite@embojournal.org

** Click here to be directed to your login page: https://emboj.msubmit.net

EMBO Press Author Checklist

Corresponding Author Name: Haihua Liang	
Journal Submitted to: The EMBO Journal	
Manuscript Number: EMBOJ-2026-124104	

USEFUL LINKS FOR COMPLETING THIS FORM
The EMBO Journal - Author Guidelines
EMBO Reports - Author Guidelines
Molecular Systems Biology - Author Guidelines
EMBO Molecular Medicine - Author Guidelines

Reporting Checklist for Life Science Articles (updated January

This checklist is adapted from Materials Design Analysis Reporting (MDAR) Checklist for Authors. MDAR establishes a minimum set of requirements in transparent reporting in the life sciences (see Statement of Task: 10.31222/osf.io/9sm4x). Please follow the journal's guidelines in preparing your manuscript.

Please note that a copy of this checklist will be published alongside your article.

Abridged guidelines for figures

1. Data

The data shown in figures should satisfy the following conditions:

- the data were obtained and processed according to the field's best practice and are presented to reflect the results of the experiments in an accurate and unbiased manner.
- ideally, figure panels should include only measurements that are directly comparable to each other and obtained with the same assay.
- plots include clearly labeled error bars for independent experiments and sample sizes. Unless justified, error bars should not be shown for technical
- if n<5, the individual data points from each experiment should be plotted. Any statistical test employed should be justified.
- Source Data should be included to report the data underlying figures according to the guidelines set out in the authorship guidelines on Data

2. Captions

Each figure caption should contain the following information, for each panel where they are relevant:

- a specification of the experimental system investigated (eg cell line, species name).
- the assay(s) and method(s) used to carry out the reported observations and measurements.
- an explicit mention of the biological and chemical entity(ies) that are being measured.
- an explicit mention of the biological and chemical entity(ies) that are altered/varied/perturbed in a controlled manner.
- the exact sample size (n) for each experimental group/condition, given as a number, not a range;
- a description of the sample collection allowing the reader to understand whether the samples represent technical or biological replicates (including how many animals, litters, cultures, etc.).
- a statement of how many times the experiment shown was independently replicated in the laboratory.
- definitions of statistical methods and measures:
  - common tests, such as t-test (please specify whether paired vs. unpaired), simple χ2 tests, Wilcoxon and Mann-Whitney tests, can be unambiguously identified by name only, but more complex techniques should be described in the methods section;
  - are tests one-sided or two-sided?
  - are there adjustments for multiple comparisons?
  - exact statistical test results, e.g., P values = x but not P values < x;
  - definition of 'center values' as median or average;
  - definition of error bars as s.d. or s.e.m.

Please complete ALL of the questions below.
Select "Not Applicable" only when the requested information is not relevant for your study.

Materials

Newly Created Materials	Information included in the manuscript?	In which section is the information available? (Reagents and Tools Table, Materials and Methods, Figures, Data Availability Section)
New materials and reagents need to be available; do any restrictions apply?	Yes	pME6032-ped1, pGEX-6p-1-ped1, PaoP5Δped1) are available upon request

Antibodies	Information included in the manuscript?	In which section is the information available? (Reagents and Tools Table, Materials and Methods, Figures, Data Availability Section)
For **antibodies** provide the following information:<br>- Commercial antibodies: RRID (if possible) or supplier name, catalogue number and or/clone number<br>- Non-commercial: RRID or citation	Yes	The following antibodies were used: anti-Flag M2 (Sigma), anti-His,

DNA and RNA sequences	Information included in the manuscript?	In which section is the information available? (Reagents and Tools Table, Materials and Methods, Figures, Data Availability Section)
**Short novel DNA or RNA including primers, probes**: provide the sequences.	Yes	All primer sequences are listed in Dataset EV5.

Cell materials	Information included in the manuscript?	In which section is the information available? (Reagents and Tools Table, Materials and Methods, Figures, Data Availability Section)
**Cell lines:** Provide species information, strain. Provide accession number in repository **OR** supplier name, catalog number, clone number, and/**OR** RRID.	Yes	Methods (Plasmids construction, Quantitative real-time PCR, Knockout of
**Primary cultures:** Provide species, strain, sex of origin, genetic modification status.	Not Applicable	Primary cell culture was not used.
Report if the cell lines were recently **authenticated** (e.g., by STR profiling) and tested for mycoplasma contamination.	Not Applicable	This study used bacterial strains and did not involve mammalian cell line authentication or mycoplasma detection.

Experimental animals	Information included in the manuscript?	In which section is the information available? (Reagents and Tools Table, Materials and Methods, Figures, Data Availability Section)
**Laboratory animals or Model organisms:** Provide species, strain, sex, age, genetic modification status. Provide accession number in repository **OR** supplier name, catalog number, clone number, **OR** RRID.	Yes	Use Galleria mellonella larvae, at the final age, approximately 250-300 mg. See Materials and Methods (Galleria Mellonella Killing Assay)
**Animal observed in or captured from the field:** Provide species, sex, and age where possible**.**	Not Applicable	
Please detail **housing and husbandry conditions**.	Yes	The larvae of the large wax moth should be stored at room temperature away from light.

Plants and microbes	Information included in the manuscript?	In which section is the information available? (Reagents and Tools Table, Materials and Methods, Figures, Data Availability Section)
**Plants:** provide species and strain, ecotype and cultivar where relevant, unique accession number if available, and source (including location for collected wild specimens).	Not Applicable	
**Microbes:** provide species and strain, unique accession number if available, and source.	Yes	

Human research participants	Information included in the manuscript?	In which section is the information available? (Reagents and Tools Table, Materials and Methods, Figures, Data Availability Section)
If collected and within the bounds of privacy constraints report on age, sex and gender or ethnicity for all study participants.	Not Applicable	

Core facilities	Information included in the manuscript?	In which section is the information available? (Reagents and Tools Table, Materials and Methods, Figures, Data Availability Section)
If your work benefited from core facilities, was their service mentioned in the acknowledgments section?	Yes	Crystal diffraction data were collected from the BL19u1 line station of the Shanghai Synchrotron Radiation Facility (SSRF). It has been described in Materials and Methods (Crystallization, data collection, processing, model building and refinement).

Design

Study protocol	Information included in the manuscript?	In which section is the information available? (Reagents and Tools Table, Materials and Methods, Figures, Data Availability Section)

If study protocol has been **pre-registered, provide DOI in the manuscript**. For clinical trials, provide the trial registration number **OR** cite DOI.	Not Applicable	
Report the **clinical trial registration number** (at ClinicalTrials.gov or equivalent), where applicable.	Not Applicable	

Laboratory protocol	Information included in the manuscript?	In which section is the information available? (Reagents and Tools Table, Materials and Methods, Figures, Data Availability Section)
Provide DOI OR other citation details if **external detailed step-by-step protocols** are available.	Yes	CRISPR - Cas9 phage knockout method reference published scheme (https://doi.org/10.1038/s41564-024-01719-5), See Materials and Methods (Knockout of phage genes using the CRISPR-Cas9 system).

Experimental study design and statistics	Information included in the manuscript?	In which section is the information available? (Reagents and Tools Table, Materials and Methods, Figures, Data Availability Section)
Include a statement about **sample size** estimate even if no statistical methods were used.	Yes	The sample size (n value) has been clearly stated in the footnotes of each figure (e.g., n=100 for cell length analysis, n=20 for Galleria assay). See each Figure legends for details.
Were any steps taken to minimize the effects of subjective bias when allocating animals/samples to treatment (e.g. **randomization procedure**)? If yes, have they been described?	Yes	The sample size (n value) has been clearly stated in the footnotes of each figure (e.g., n=100 for cell length analysis, n=15-20 for Galleria assay). See each Figure legends for details.
Include a statement about **blinding** even if no blinding was done.	Not Applicable	
Describe **inclusion/exclusion criteria** if samples or animals were excluded from the analysis. Were the criteria pre-established?<br><br>If sample or data points were omitted from analysis, report if this was due to attrition or intentional exclusion and provide justification.	Not Applicable	
For every figure, are **statistical tests** justified as appropriate? Do the data meet the assumptions of the tests (e.g., normal distribution)? Describe any methods used to assess it. Is there an estimate of variation within each group of data? Is the variance similar between the groups that are being statistically compared?	Yes	The statistical methods have been described in the notes to each figure (such as two-way ANOVA with Dunnett's test, one-way ANOVA with Dunnett's test, log-rank test). The error bar is defined as SD. See the corresponding statistical descriptions in each Figure legends and Materials and Methods.

Sample definition and in-laboratory replication	Information included in the manuscript?	In which section is the information available? (Reagents and Tools Table, Materials and Methods, Figures, Data Availability Section)
In the figure legends: state number of times the experiment was **replicated** in laboratory.	Yes	All the captions have been marked with "Data are from three independent experiments" or similar expressions. See each Figure legends for details.
In the figure legends: define whether data describe **technical or biological replicates**.	Yes	Biological repeats have been indicated in the figure caption, such as n=100 for cell length, n=3 independent experiments. See each Figure legends for details.

Ethics

Ethics	Information included in the manuscript?	In which section is the information available? (Reagents and Tools Table, Materials and Methods, Figures, Data Availability Section)
Studies involving **human participants**: State details of **authority granting ethics approval** (IRB or equivalent committee(s), provide reference number for approval.	Not Applicable	
Studies involving **human participants**: Include a statement confirming that **informed consent** was obtained from all subjects and that the experiments conformed to the principles set out in the WMA Declaration of Helsinki and the Department of Health and Human Services Belmont Report.	Not Applicable	
Studies involving **human participants:** For publication of **patient photos**, include a statement confirming that consent to publish was obtained.	Not Applicable	
Studies involving experimental **animals**: State details of **authority granting ethics approva**l (IRB or equivalent committee(s), provide reference number for approval. Include a statement of compliance with ethical regulations.	Yes	The giant wax moth, as an invertebrate model, usually does not require ethical approval.
Studies involving **specimen and field samples:** State if relevant **permits** obtained, provide details of authority approving study; if none were required, explain why.	Not Applicable	

Dual Use Research of Concern (DURC)	Information included in the manuscript?	In which section is the information available? (Reagents and Tools Table, Materials and Methods, Figures, Data Availability Section)
Could your study fall under dual use research restrictions? Please check biosecurity documents and list of **select agents and toxins** (CDC): https://www.selectagents.gov/sat/list.htm	Not Applicable	
If you used a select agent, is the security level of the lab appropriate and reported in the manuscript?	Not Applicable	
If a study is subject to dual use research of concern regulations, is the name of the **authority granting approval and reference number** for the regulatory approval provided in the manuscript?	Not Applicable	

Reporting

The MDAR framework recommends adoption of discipline-specific guidelines, established and endorsed through community initiatives. Journals have their own policy about requiring specific guidelines and recommendations to complement MDAR.

Adherence to community standards	Information included in the manuscript?	In which section is the information available? (Reagents and Tools Table, Materials and Methods, Figures, Data Availability Section)
State if relevant guidelines or checklists (e.g., **ICMJE, MIBBI, ARRIVE, PRISMA**) have been followed or provided.	Yes	This study followed the EMBO Press author guidelines and MDAR reporting standards.
For **tumor marker prognostic studies,** we recommend that you follow the **REMARK** reporting guidelines (see link list at top right). See author guidelines, under 'Reporting Guidelines'. Please confirm you have followed these guidelines.	Not Applicable	
For **phase II and III randomized controlled trials**, please refer to the **CONSORT** flow diagram (see link list at top right) and submit the CONSORT checklist (see link list at top right) with your submission. See author guidelines, under 'Reporting Guidelines'. Please confirm you have submitted this list.	Not Applicable	

Data Availability

Data availability	Information included in the manuscript?	In which section is the information available? (Reagents and Tools Table, Materials and Methods, Figures, Data Availability Section)
Have **primary datasets** been deposited according to the journal's guidelines (see 'Data Deposition' section) and the respective accession numbers provided in the Data Availability Section?	Yes	RNA-seq data have been uploaded to BioProject (accession: PRJNA1256806). For details, see Materials and Methods (RNA-seq and data analysis) and the Data availability statement.
Were **human clinical and genomic datasets** deposited in a public access-controlled repository in accordance to ethical obligations to the patients and to the applicable consent agreement?	Not Applicable	
Are **computational models** that are central and integral to a study available without restrictions in a machine-readable form? Were the relevant accession numbers or links provided?	Not Applicable	
If publicly available data were reused, provide the respective **data citations in the reference list**.	Not Applicable	
